# Non-Muscle-Invasive Bladder Carcinoma with Respect to Basal Versus Luminal Keratin Expression

**DOI:** 10.3390/ijms21207726

**Published:** 2020-10-19

**Authors:** Minsun Jung, Insoon Jang, Kwangsoo Kim, Kyung Chul Moon

**Affiliations:** 1 Department of Pathology, Seoul National University College of Medicine, Seoul 03080, Korea; jjunglammy@gmail.com; 2 Biomedical Research Institute, Seoul National University Hospital, Seoul 03080, Korea; isjang1324@gmail.com; 3 Transdisciplinary Department of Medicine & Advanced Technology, Seoul National University Hospital, Seoul 03080, Korea; kksoo716@gmail.com; 4 Kidney Research Institute, Medical Research Center, Seoul National University College of Medicine, Seoul 03080, Korea

**Keywords:** non-muscle-invasive bladder cancer, urinary bladder neoplasms, gene expression profiling, keratin-5/6, keratin-20, biomarkers

## Abstract

Non-muscle-invasive bladder cancer (NMIBC) consists of transcriptional subtypes that are distinguishable from those of muscle-invasive cancer. We aimed to identify genetic signatures of NMIBC related to basal (K5/6) and luminal (K20) keratin expression. Based on immunohistochemical staining, papillary high-grade NMIBC was classified into K5/6-only (K5/6^High^-K20^Low)^, K20-only (K5/6^Low^-K20^High^), double-high (K5/6^High^-K20^High^), and double-low (K5/6^Low^-K20^Low^) groups (*n* = 4 per group). Differentially expressed genes identified between each group using RNA sequencing were subjected to functional enrichment analyses. A public dataset was used for validation. Machine learning algorithms were implemented to predict our samples against UROMOL subtypes. Transcriptional investigation demonstrated that the K20-only group was enriched in the cell cycle, proliferation, and progression gene sets, and this result was also observed in the public dataset. The K5/6-only group was closely regulated by basal-type gene sets and showed activated invasive or adhesive functions. The double-high group was enriched in cell cycle arrest, macromolecule biosynthesis, and *FGFR3* signaling. The double-low group moderately expressed genes related to cell cycle and macromolecule biosynthesis. All K20-only group tumors were classified as UROMOL “class 2” by the machine learning algorithms. K5/6 and K20 expression levels indicate the transcriptional subtypes of NMIBC. The K5/6^Low^-K20^High^ expression is a marker of high-risk NMIBC.

## 1. Introduction

Bladder cancer represents the 9th most common malignancy, with approximately 430,000 new cases and 165,000 new cancer-related deaths worldwide in 2012 [1]. Influenced by tobacco usage, Europe and North America are among the regions with the highest incidence and mortality rates [1]. Approximately 75% of these cases present with non-muscle-invasive bladder cancer (NMIBC) or mucosa-confined or submucosa-invasive disease [2]. Despite its overall favorable survival, NMIBC recurs frequently and can eventually progress to muscle-invasive bladder cancer (MIBC), which necessitates repeated cystoscopic resection or even radial cystectomy [2,3]. Identification of genetic characteristics of NMIBC could be the first step to accurately predicting patient prognoses and to providing personalized treatment.

Accumulating transcriptional data suggest that NMIBC consists of intrinsic subtypes that are more than just underdeveloped counterparts of those of MIBC [4]. Although some transcriptional subtypes of bladder cancer are identified over pathological stages, the proportion of molecular subtypes varies by stage, and subtypes also can change along with stage progression [4,5]. Moreover, the clinical behavior of molecular subtypes differs between NMIBC and MIBC. For example, one of the intrinsic subtypes of NMIBC that has a poor prognosis showed molecular skewness to luminal cells rather than basal cells of the urothelium, and displayed high levels of K20/uroplakins but low levels of K5/K15, which was transcriptionally akin to but clinically different from the luminal type of MIBC [6]. The aggressiveness of luminal-like NMIBC is known to stem from the enrichment of the cell cycle, changes in junctional complexes, or high copy number alteration [6,7,8]. K5/6 and K20 have been widely used as surrogate markers of intrinsic subtypes of NMIBC, and their expression is significantly associated with the clinical outcomes of NMIBC [9,10,11]. In addition, it was demonstrated that immunohistochemical (IHC) expression of K5/6 and K20 was associated with the molecular biology of non-muscle-invasive upper tract urothelial carcinoma (NMIUTUC), including proliferation, cell adhesion, and the mitogen-activated protein kinase (MAPK) pathway [12,13]. The application of IHC staining for basal and luminal proteins to the management of patients with MIBC has been discussed [14,15,16]; furthermore, molecular characterization of intrinsic subtypes of NMIBC can aid in predicting tumor progression to muscle-invasive diseases [4]. The genetic implications of the basal-like phenotype (K5/6^High^-K20^Low^) and luminal-like phenotype (K5/6^Low^-K20^High^) were reported in previous studies, which included both NMIBC and MIBC [5,17,18]. However, the association between NMIBC gene expression profiles and K5/6 and K20 expression, especially double high or double low for K5/6 and K20 expression is still unclear. Moreover, immunophenotype ambiguity of basal and luminal proteins, which is often observed in papillary NMIBC as opposed to MIBC, remains a challenge, further underlining the importance of genetic characterization of NMIBC with different K5/6 and K20 expression profiles [12,19].

In this study, we aimed to uncover the gene expression profiles of NMIBC stratified by basal keratin (K5/6) and luminal keratin (K20) expression. Differentially expressed genes (DEGs) and functional enrichment were assessed between tumor groups defined by IHC staining for K5/6 and K20 using RNA sequencing.

## 2. Results

### 2.1. Patient Characteristics

In total, 4 groups consisting of 16 papillary high-grade NMIBC specimens (*n* = 4 each) were assessed using IHC staining for K5/6 and K20: a K5/6-only group (K5/6^High^-K20^Low^), a K20-only group (K5/6^Low^-K20^High^), a double-high group (K5/6^High^-K20^High^), and a double-low group (K5/6^Low^-K20^Low^) were assembled (Figure S1). The demographic, clinicopathological, and IHC details are presented in Table S1. The median age was 70 years (range, 58–83) and the male-to-female ratio was 4.3:1. Submucosal invasion was observed in 75% (12/16) of patients. One of the K20-only group specimens and one of the double-low group specimens showed concurrent urothelial carcinoma in situ. Except for K5/6 (*p* = 0.003) and K20 (*p* = 0.004) expression, the groups had even distributions of clinicopathological parameters and other IHC results. The overexpression pattern of *p*53 was observed in 0%, 75%, 50%, and 25% of K5/6-only, K20-only, double-high, and double-low group tumors, respectively. During the follow-up period, 1 K5/6-only, 3 K20-only, and 3 double-low group NMIBC patients experienced local recurrence (Figure S2).

### 2.2. Differentially Expressed Genes

**Figure 1:**
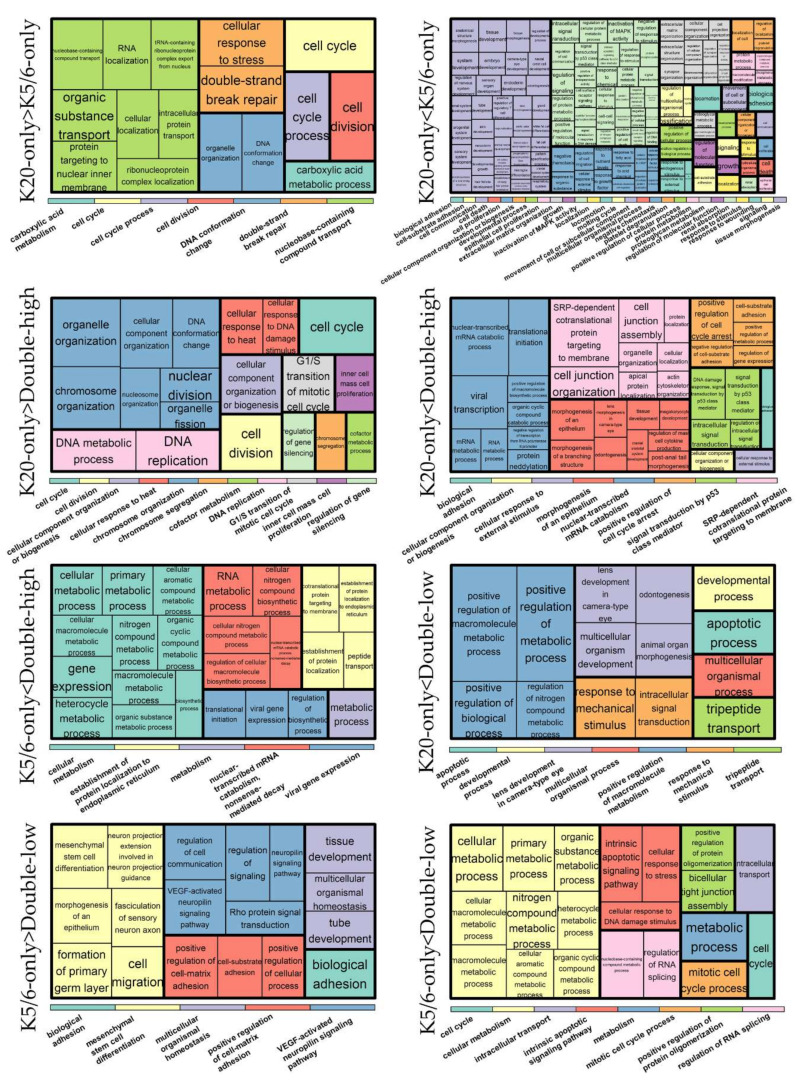
Gene ontology-biologic processes enriched in upregulated genes in each comparison. The relative size of the rectangles represents statistical significance levels. The overarching gene ontology categories are demonstrated with color bars.

We identified 1462 DEGs across the NMIBC groups (K20-only vs. K5/6-only, K20-only vs. double-high, K5/6-only vs. double-high, K20-only vs. double-low, and K5/6-only vs. double-low), as illustrated in Figure S3. Upregulated and downregulated genes were separately subjected to Gene ontology (GO) and Kyoto Encyclopedia of Genes and Genomes (KEGG) databases (Figure 1; Table S2).

As a result, cell cycle progression and DNA repair were enriched in genes that were highly expressed in the K20-only group compared to the K5/6-only or double-high groups (e.g., GO “cell cycle” and “double-strand break repair via homologous recombination”; KEGG “cell cycle” and “DNA replication”) and in those highly expressed in the double-low group compared to the K5/6-only group (e.g., GO “cyclin B1-CDK1 complex” and “G2/M transition of mitotic cell cycle”; KEGG “cell cycle” and “DNA replication”). Conversely, relative to the K20-only group, cell cycle arrest and/or cell death were enriched in DEGs upregulated in the K5/6-only group (e.g., GO “intrinsic apoptotic signaling pathway by p53 class mediator” and “positive regulation of cell death”; KEGG “apoptosis”) and in the double-high group (e.g., GO “positive regulation of cell cycle arrest” and “DNA damage response signal transduction by p53 class mediator”). Second, genes significantly overexpressed in the double-high group (vs. K20-only or K5/6-only) and the double-low group (vs. K20-only) were skewed toward functions related to protein synthesis/metabolism (e.g., GO “protein targeting to ER”, “cellular nitrogen compound biosynthetic process”, and “positive regulation of nitrogen compound metabolic process”; KEGG “ribosome’). In addition, DEGs found to be highly expressed in the K5/6-only or double-high groups compared to the K20-only or double-low groups were overrepresented by tissue morphogenesis and cell/substrate binding functions (e.g., GO “tissue morphogenesis” and “cell-substrate adhesion”; KEGG “focal adhesion” and “tight junction”). Signaling pathways, such as the PI3K-Akt, MAPK, and HIF pathways, were related to the upregulated genes of the K5/6-only group and, to a lesser extent, to those of the double-high group compared to the K20-only or double-low groups. Additionally, the K5/6-only group overexpressed genes in the membership of cell migration (e.g., GO “regulation of cell migration”; KEGG “regulation of actin cytoskeleton”) compared to the K20-only group.

### 2.3. Ingenuity Pathway Analysis and Gene Set Enrichment Analysis

**Figure 2:**
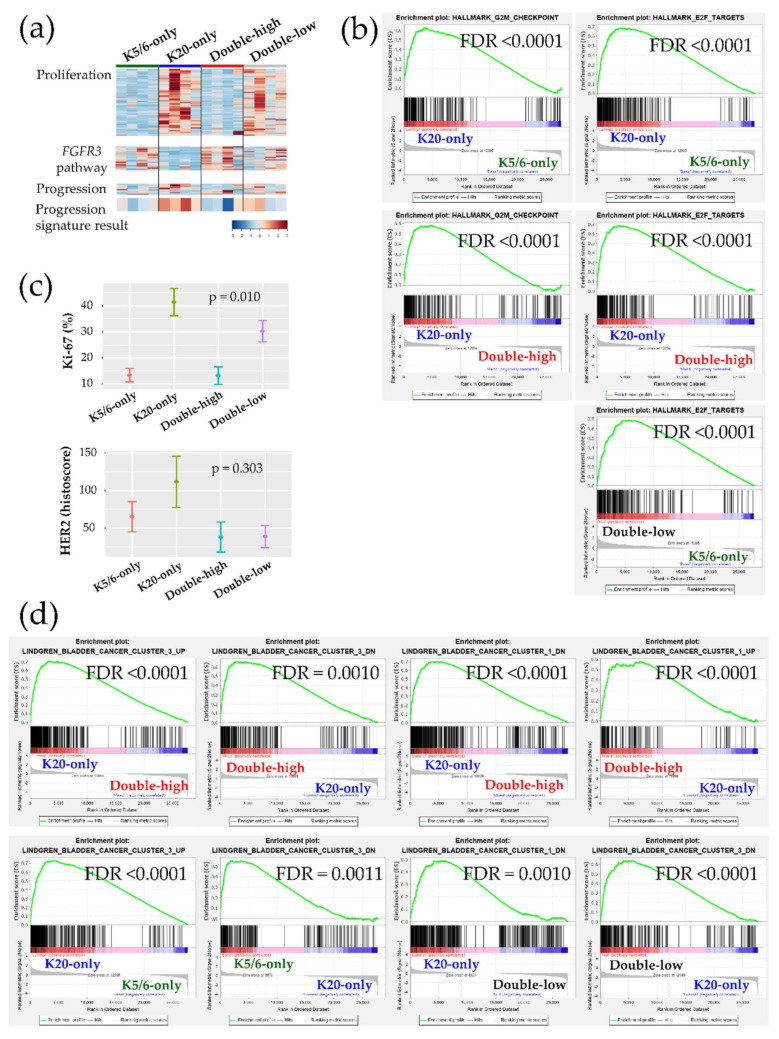
Biologic and functional signatures of the non-muscle-invasive bladder cancer (NMIBC) groups. (**a**) Heatmaps of genes included in the proliferation signature (top), the *FGFR3* pathway (middle), and the progression signature (bottom). The progression result was obtained by subtracting the low-expression genes from the high-expression genes included in the NMIBC progression gene set. (**b**) Hallmark gene sets related to cell proliferation from the GSEA. (**c**) Immunohistochemical (IHC) staining results of Ki-67 (%) and HER2 (histoscore) for each group are illustrated as the mean (dot) and standard deviation (bar). (**d**) Curated gene sets from the GSEA (“Lindgren_bladder_cancer”) that were significantly enriched in the groups.

Ingenuity Pathway Analysis (IPA) was conducted using each DEG set (Table 1). Transcription factors promoting the cell cycle were activated in the K20-only group (e.g., *RABL6*, *ID2*, *ID3*, *MYC*, and *E2F3* of the E2F family) vs. the K5/6-only, double-high, or double-low groups; while, those participating in cell cycle checkpoints and cell death were activated in the K5/6-only and double-high groups (e.g., *TP53*, *TP63*, *CDKN2A*, *CDKN1A*, *RB1,* and *NURP1*) vs. the K20-only or double-low groups. These enhanced cell cycle and cell survival themes indicate that many cancer-progressive functions converge in the K20-only group compared to the double-high or double-low groups; in contrast, the double-high group was especially enriched for various cytostatic terms. Likewise, Gene Set Enrichment Analysis (GSEA) demonstrated enrichment of cell proliferation and related machinery in the K20-only group and also in the double-low group relative to the K5/6-only or double-high groups (Figure 2a,b, Figure S4). In addition, *ERBB2* and *ERBB3* were suggested to positively regulate the K20-only group (Table 1). Consistent with these transcriptional findings, the Ki-67 proliferative index was high in the K20-only group (*p* = 0.010) and HER2 expression was relatively high in the K20-only group as well as the K5/6-only group (*p* = 0.303) in IHC staining (Kruskal-Wallis; Figure 2c). Furthermore, lipid and glucose metabolism was enriched in the K20-only group vs. the double-high or K5/6-only groups according to IPA (e.g., *PPARGC1A* and *SREBF2*) and GSEA Figure S5. On the other hand, the K5/6-only group was conjectured to more strongly harbor invasive or adhesive properties than the K20-only group, which was accompanied by activated MAPK pathway components (e.g., *EGF*, *IGF1*, *RAF1*, and *ERK1*/*ERK2*) and TGF-β/*CTNNB1* (Table 1). Finally, compared to the K20-only group, the K5/6-only group was enriched with activated genes involved in chromatin modification, including *KDM5B* and *SMARCA4* (Table 1).

Next, we further evaluated our cohort against the previously established genetic signatures. The signature of the *FGFR3* signaling pathway was relatively decreased in the K20-only group Figure 2a. Conversely, a panel of 12 genes implicated in NMIBC progression indicated that K20-only group tumors were more prone to advance to MIBC and/or to a life-threatening state (Figure 2a). Interestingly, an investigation of GSEA “curated gene sets” revealed that NMIBC “cluster 1” and “cluster 3” defined by Lindgren et al. substantially overlapped with our NMIBC groups [8]: The K20-only group was biased toward Lindgren’s cluster 3 but away from Lindgren’s cluster 1; however, the double-high group showed the opposite trend (Figure 2d). In comparison to the K20-only group, the K5/6-only group and the double-low group overexpressed the genetic signatures downregulated in Lindgren’s cluster 3 (Figure 2d). Furthermore, the upregulated signals of breast basal cells vs. luminal cells were significantly downregulated in the K20-only group (Figure S6). Finally, machine learning prediction tools were used to classify the present specimens to the UROMOL NMIBC classes (“class 1”,”class 2”, or “class 3”) Table S3 [6]. All K20-only group tumors were classified as UROMOL class 2, the double-low group was classified as either the UROMOL class 2 or UROMOL class 3, and the K5/6-only and double-high groups were mostly annotated as UROMOL class 3 with high accuracy.

### 2.4. Validation Using the Public Gene-Expression Dataset

Upon the premise of the IHC-guided DEG analyses, 23 patients of the Lund NMIBC cohort met the clinicopathological and IHC criteria (Table S4) [5,17]. The median age was 75 (range, 52–87) years and the male-to-female sex ratio was 3.6:1. Seven (30.4%) and 4 (17.4%) tumors were stage T1 and grade 3, respectively.

**Figure 3:**
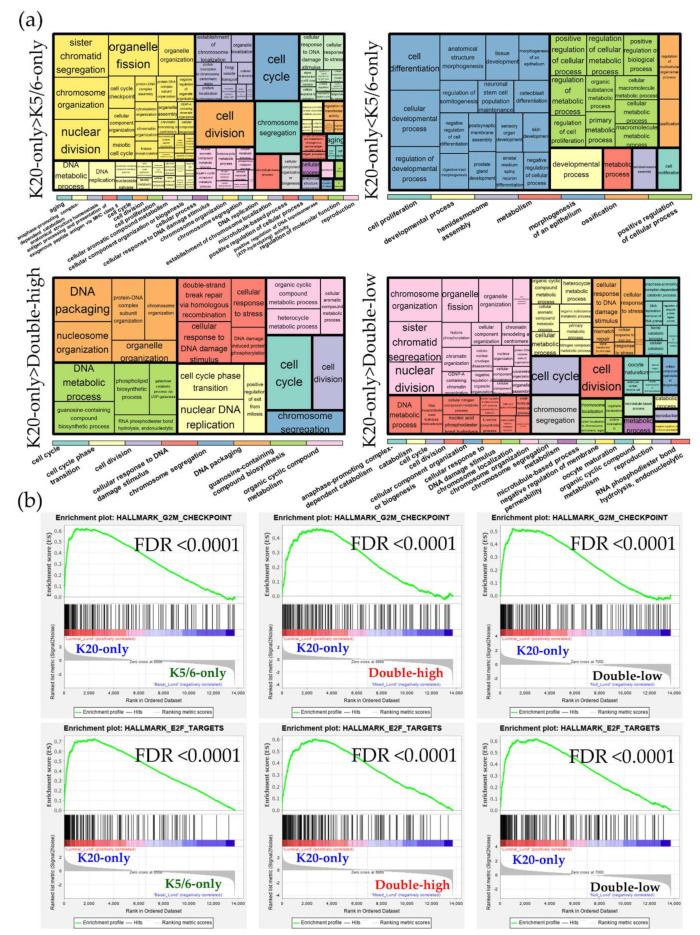
Functional enrichment analyses of the differentially expressed genes (DEGs) identified in the Lund groups. (**a**) Gene ontology-biologic processes enriched in the upregulated genes from each comparison. The overarching gene ontology categories are demonstrated with color bars (**b**) Hallmark gene sets related to cell proliferation from the Gene Set Enrichment Analysis (GSEA).

DEG sets identified across the Lund cohort groups were analyzed using the GO and KEGG databases Figure S7. Similar to the results of our NMIBC cohorts, the K20-only_Lund group was overrepresented by cell cycle and E2F functions compared to the other groups; however, compared to the K20-only_Lund group, the K5/6-only_Lund group had increased levels of genes involved in epithelium morphogenesis and cellular biologic processes (Figure 3; Table S5). According to the IPA study (Table 2), enrichment of cell cycle progression and cell proliferation were predicted in the K20-only_Lund group compared to the other groups, as indicated by altered upstream regulators (e.g., *RABL6*, *FOXM1*, *MITF*, and *E2F3*) and pathological terms (e.g., “M phase”, “segregation of chromosomes”, and “cell survival”). In addition, consistent with the foregoing findings for the K5/6-only group, activation of *KDM5B* was predicted in the K5/6-only_Lund group compared to the K20-only_Lund or double-high_Lund groups (Table 2).

## 3. Discussion

It has become clear that NMIBC has discrete transcriptional subtypes that are distinguishable from those of MIBC [6,8]. We performed IHC staining for K5/6 and K20 as subtype-defining markers in papillary high-grade NMIBC and investigated the functional implications through RNA sequencing. In addition to their correlation to molecular profiles of bladder cancer [9,17,20], immunostains for K5/6 and K20 have an advantage in that they are widely used in real-world practice. As a result, the highest signals related to cell proliferation, survival, and advanced malignancy and brisk Ki-67 proliferative index were found in the K20-only group, which was successfully validated in the independent K20-only_Lund group. Enrichment of DNA repair functions, one of the characteristics of high-risk aggressive members of NMIBC, was also observed in the K20-only group and K20-only_Lund group [7]. On the other hand, the transcriptional characteristics of the double-high group included cell cycle arrest, apoptosis, tissue morphogenesis, and protein synthesis/metabolism. The K5/6-only group was characterized by enrichment of basal MIBC markers (e.g., *TP63, IKBKB, HIF1A, EGF*, *STAT3*, and PI3K-Akt, MAPK, and HIF pathways), while the K20-only group had similar molecular profiles with luminal subtype MIBC (e.g., *PPARGC1A*, *SREBF2*, *ERBB2*, *ERBB3*, and *ESR1*) [20]. Notably, other IHC expression related to MIBC subtypes in this study, including GATA3 and FOXA1, did not differ among our groups [14]. In NMIBC, therefore, IHC staining for K5/6 and K20 has demonstrated its value in reflecting the conventional luminal/basal subtypes of MIBC.

Several biologic characteristics shared in both the K20-only group and the K20-only_Lund group underscore the high-risk characteristics of NMIBC with a luminal phenotype, concurring other studies where aggressive subtypes of NMIBC have high K20 and low K5/6 expression [5,6,11,17]. High levels of genes related to cell proliferation and DNA repair have been commonly found in high-risk subtypes of early urothelial carcinoma [6,7,13,21]. Various machine learning classifiers stably annotated the K20-only group as UROMOL class 2, a luminal-like subtype of NMIBC that showed high proliferation signatures, aggressive clinical features, and poor survival outcomes [6]. Moreover, it is worth noting that the present groups considerably reproduced the NMIBC clusters defined by Lindgren et al. [8]: GSEA clearly connected our K20-only and double-high groups to Lindgren’s cluster 3 and cluster 1, respectively. It was reported that Lindgren’s cluster 3, consisting of high-grade NMIBC accompanied by concurrent carcinoma in situ, highly expressed genes coding for cell cycle GO processes, but Lindgren’s cluster 1, which showed low grade and long recurrence-free survival, had low expression of cell cycle-related genes and high expression of protein synthesis/ribosome-related genes [8]. Through the 12-gene signature, we also showed that the K20-only group and the double-high group were associated with high and low risks of disease progression, respectively [22]. Thus, IHC staining for K5/6 and K20 manifests both molecular and clinical relevance, and it is feasible to maintain the usefulness of these proteins as markers of the NMIBC subtypes and prognostic factors of patients with NMIBC. Based on the IPA and GSEA findings, we hypothesize that active *E2F3*, which is coordinated with deactivated *Rb*, is the main contributor to K20-only group progression [23]. E2Fs are a group of transcription factors orchestrating the cell cycle, apoptosis, DNA synthesis, and repair; these proteins have been known to be responsible for carcinogenesis, invasion, and progression of urothelial carcinoma [23,24,25]. In mice, activation of *E2F3* driven by deregulated *Rb*-induced high-grade papillary bladder cancer [23,24]. *E2F3*, as a contributor to early cell cycle progression, may regulate early cell cycle genes in the K20-only group (e.g., *ID2* and *ID3*). On the contrary, early cell cycle genes were mostly enriched in low-risk transcriptional subtypes of NMIBC in previous studies [6,7]. Considering that there are architectural and grade disparities between our study (papillary high-grade) and previous studies (various growth patterns and grades) and that basal/luminal proteins might be expressed discretely by tumor grade [6,7,11], we speculate that regulation of the tumor cell cycle might be affected by different pathological traits. In addition, enrichment of the *ERBB2* signature and relative overexpression of IHC staining for HER2 in the K20-only group coincides with the “HER2-like” subtype, another major high-risk subset of NMIBC, which suggests that HER2 blockade could be explored as a treatment option for K20-only group tumors [21]. The enrichment of mTORC1 and cholesterol/fatty acid/glucose metabolism, which was previously shown to be related to high tumor grade and short overall survival in NMIBC, further supports the high-risk phenotypes of the K20-only group [7].

*FGFR3* alteration is one of the molecular hallmarks of early carcinogenesis of the urothelium and is associated with increased survival in NMIBC [25]. We demonstrated that both the *FGFR3* gene (|fold change| = 4.7, *p* = 0.04, double-high vs. K20-only) and its associated signatures were downregulated in the K20-only group but enriched in the double-high group. Notably, Lindgren’s cluster 1 is more closely related to *FGFR3* alteration, such as a higher mutation rate and expression level, than Lindgren’s cluster 3 [8]. Mutation-driven hyperactivation of *FGFR3* is one of the major triggers of low-risk NMIBC that frequently co-occurs with *PIK3CA* mutation [25]. Consistent with this, we found that the double-high group was enriched in PI3K-Akt signaling. A pan-FGFR inhibitor, erdafitinib, displayed a meaningful tumor response rate in patients with urothelial carcinoma, indicating that it could be especially beneficial to those with *FGFR3*-activated tumors, suggesting a promising targetable axis of the double-high group [21,26]. In addition, previous studies showed that K5/6 and K20 dual positivity marked well-differentiated tumors that maintained tissue architectural hierarchy, which was demonstrated to be a favorable prognostic factor for early urothelial carcinoma [11,12,13]. We also found that upregulated genes in the double-high group, consistent with Lindgren’s cluster 1, were involved in numerous themes of protein synthesis and metabolism [8]. Similarly, cytoskeletal, junctional, and cell interaction pathways supporting architecture integrity and metabolic pathways supporting homeostasis were found to be subtype-specific functions of an indolent NMIBC subtype discovered by proteome recently [27]. Thus, it is reasonable to speculate that the differentiation and tissue organization of the double-high group is maintained by active intercellular interactions that are enriched for epithelium morphogenesis, and cell-cell and cell-substrate binding functions.

Despite the high expression of cell cycle regulatory molecules and low expression of the cell cycle progression signature, the K5/6-only group was predicted to have activated functions of tumor cell adhesion, migration, and invasion and enhanced TGF-β cascades [20,28]. High levels of K14 in the K5/6-only group indicate its connection to the basal-like state and migratory function, in accordance with previous reports that K14 was mainly expressed in tumors with high K5/6 and low K20 expression and K14 expression identified stemness and basal/squamous-like characteristics, including activated cellular movement, in urothelial carcinoma [13,28]. Parallel to this, we also found a trend of tumors showing *p*53-wildtype staining clustered in the K5/6-only group compared to the K20-only group, which frequently displayed *p*53 overexpression. Aberrant expression of *p*53 differs among NMIBC subtypes and can indicate impending stage progression of the “genomically unstable” or “urothelial-like C” subtypes that are genetically similar to our K20-only group [4]. Instead, the lack of p53 overexpression in the K5/6-only group, consistent with the “urothelial-like B” or “basal/squamous-like” subtypes, implies a distinct regulatory pathway of tumor progression in the K5/6-only group, which warrants further investigation [4]. In addition, chromatin-modifying genes, such as *KDM5B* and *SMARCA4*, were activated in both the K5/6-only and K5/6-only_Lund groups more than would be expected randomly. Together with recent mutational data [29], findings regarding these molecules reveal an opportunity for targeted therapy in K5/6^High^-K20^Low^ NMIBC.

The double-low phenotype, or negative expression of basal and luminal markers, was reported only in a handful of urinary bladder carcinomas. The double-low phenotype was once characterized by low expression of claudin and high expression of genes targeted by *TP53* in a previous study [30]. However, we failed to find such trends in the double-low group among claudin-related genes (e.g., *CLDN*, *CDH1*, *VIM*, and *SNAI2*) and *TP53*-related signatures. Instead, we revealed that the double-low group had a moderate expression of cell cycle progression-relate genes, in between that of the K20-only group and that of the K5/6-only group, and had a higher level of a protein synthetic/metabolic signature genes than the K20-only group. IHC staining for K5/6 was reactive in some of the basal cells in the double-low group tumors. This staining is reminiscent of the loss of the normal expression pattern of basal-type proteins, such as K5/6 and CD44, which was significantly associated with poor outcomes of NMIBC and NMIUTUC [12,19]. In view of these findings, we hypothesize that the double-low group may indicate an advanced state of the K5/6-only group that is not yet as progressed as the K20-only group. At present, the features of the double-low group remain to be elucidated.

There are some limitations to the present study. The shortage of specimens subjected to RNA sequencing may have hindered robust statistical analysis and identification of a small but important difference in gene expression. In addition, although we attempted to apply subgroup-defining methodology similar to that in our cohort to the validation cohort, there were differences in detailed IHC staining conditions, cutoffs, and gene-expression test platforms. These differences may have induced discrepancies in the enrichment results of the Lund group, so the validation results must be evaluated carefully. Finally, the overall strategy of this study without the use of a data-driven classification framework was not adequate for discovering new intrinsic subtypes of NMIBC. Nevertheless, the molecular insights gained from this study could provide an efficient way to apply the vast genetic information of NMIBC to the real-world practice using the most common IHC markers, K5/6 and K20 immunostains.

## 4. Materials and Methods

### 4.1. Specimen Selection for RNA Sequencing Using Immunohistochemical Staining for K5/6 and K20

Transurethral resection formalin-fixed paraffin-embedded specimens of papillary high-grade NMIBC that were archived in the pathology department of Seoul National University Hospital were screened using IHC staining for K5/6 (1:100; D5/16 B4; RRID:AB_2281083; Dako, Glostrup, Denmark) and K20 (1:50; Ks 20.8; RRID:AB_2133718; Dako) based on the extent of moderate-to-strong staining in tumor cells as follows: score 0 = 0%, score 1 = 0–1%, score 2 = 1–10%, score 3 = 10–25%, score 4 = 25–50%, score 5 = 50–75% and score 6 = 75–100%. Specimens that met all of the following criteria were included (*n* = 16): (i) high expression ≥ IHC score 4, (ii) low expression < IHC score 4, and (iii) when one protein was predominantly expressed, the score difference was ≥ 2. In addition, the expression levels of K14 (1:300; LL002; RRID:AB_1159418; Cell Marque, Rocklin, CA, USA), GATA3 (1:500; L50-823; Cell Marque), and FOXA1 (1:500; PA5-27157; RRID:AB_2544633; Thermo Fisher, Waltham, MA) were evaluated in the same manner. Aberrant expression of *p*53 (1:1000, DO-7, Dako) was defined as homogenous overexpression, complete absence, or cytoplasmic staining following a previous study [4]. Quantitative measurement of nuclear Ki-67 (1:100; MIB-1; RRID:AB_2631211; Agilent, Santa Clara, CA, USA) fraction (%) and membranous HER2 (ready-to-use, 4B5, Ventana, AZ, USA) histoscore [(weak staining proportion (%) multiplied by 1) + (moderate staining proportion (%) multiplied by 2) + (strong staining proportion (%) multiplied by 3)] was carried out on virtually scanned slides (Aperio AT2, Leica Biosystem, Wetzlar, Germany) using QuPath (version 0.1.2) [31]. IHC staining was conducted using the Benchmark autostainer (Ventana, Tucson, AZ, USA). The study has been approved by the institutional research ethics committee of the Seoul National University Hospital (IRB No. H-1810-148-983, 7 November 2018) and has been performed in accordance with the ethical standards as laid down in the 1964 Declaration of Helsinki and its later amendments or comparable ethical standards. A waiver of informed consent was approved by the review board because this research contains no more than minimal risk.

### 4.2. RNA Sequencing

cDNA libraries of formalin-fixed paraffin-embedded blocks cut in 10-μm-thick sections were prepared with the SureSelect^XT^ RNA Direct Reagent Kit (Agilent) [32]. Briefly, total RNA was isolated from each sample. After DNA contamination was removed using DNase, mRNA with poly-A tail was selectively enriched using an mRNA purification kit and was followed by random fragmentation. cDNA was synthetized from mRNA through reverse transcription [33]. DV200, defined as the percentage of RNA fragments > 200 nucleotides [32], was > 50% in most and > 30% in all samples, with no significant difference among the groups (Table S1). Paired-end mRNA was sequenced on the NovaSeq 6000 Sequencing System (RRID:SCR_016387; Illumina, San Diego, CA, USA) using the NovaSeq 6000 S4 Reagent Kit (Illumina). The sequencing data were preprocessed using the Trimmomatic tool [34] and mapped to the human genome (UCSC hg19) using HISAT2 [35]. The transcriptome was assembled using the StringTie tool [36]. As a result, 63,714 transcripts, corresponding to 27,680 genes, were identified across all specimens. The expression levels of *KRT5*, *KRT6A*, *KRT6B*, *KRT6C*, and *KRT20* matched the IHC profiles well (Figure S8). After the exclusion of the genes that were not expressed in any sample, a total of 9015 unique genes were used for subsequent functional studies.

In addition, the publicly available Lund NMIBC cohort (GSE32894) was investigated in a similar fashion [5,17]. To that end, MIBC and grade 1 or non-urothelial-like NMIBC were excluded. Afterward, those with upper 25% (high) and lower 25% (low) K5/6 and K20 tumor expression scores were grouped as follows: a K5/6-only_Lund group (K5/6^High^-K20^Low^), a K20-only_Lund group (K5/6^Low^-K20^High^), a double-high_Lund group (K5/6^High^-K20^High^), and a double-low_Lund group (K5/6^Low^-K20^Low^).

### 4.3. Differentially Expressed Genes and Functional Analyses

DEGs were identified between each group with *p*-value < 0.05 and absolute fold change > 2 as cutoffs. Enrichment of gene signatures was investigated using the formal “hallmark gene sets” and “curated gene sets” of GSEA [37]. GO and KEGG enrichment analyses were performed using DEGs. Significant GO terms and corresponding false discovery rate (FDR) values were submitted to ReViGO and visualized using treemaps [38]. Moreover, data were analyzed through the use of IPA [39]. The IPA functions, “upstream regulators” and “diseases and functions”, predicted regulatory molecules and pathological alteration related to the DEG sets based on the knowledge database. FDR < 0.05 was considered significant in the functional enrichment studies. Gene signatures related to cell proliferation, the *FGFR3* signaling pathway, and NMIBC progression were obtained from previous reports [5,21,22]. The progression signature was compiled by using the expression levels of genes related to NMIBC progression; the levels of low-expression genes were subtracted from those of the high-expression genes to obtain a final score of the progression signature [22].

### 4.4. Statistical Analysis

Clinicopathological and follow-up details were obtained from medical records. All tumors were treatment-naïve primary cases, except for one in the K5/6-only group that the patient received BCG treatment 3 years ago due to bladder cancer. Tumor grading followed the latest definition [2]. All patients were followed-up regularly with the cystoscopic examination. The median follow-up duration was 59 months (range, 9–90). Recurrence-free survival was calculated using the date of same-site recurrence or the last urologic follow-up visit. Clinicopathological comparison was conducted using nonparametric tests in R version 3.2.1 with a two-tailed *p*-value < 0.05 considered as significant.

To accurately assign our samples to the intrinsic subtypes of NMIBC published by Hedegaard and coworkers (UROMOL study), we decided to build a distance-based gene-expression classifier of UROMOL subtypes and employ machine learning techniques [6]. Several computational models were tested through repeated cross-validation (×10) using 110 commonly expressed genes (Figure S9). The algorithms showing high mean accuracy (>0.94) were selected to assign our samples to the UROMOL intrinsic subtypes: sparse partial least squares [40], regularized logistic regression [41], and GLMnet [42].

## 5. Conclusions

**Figure 4:**
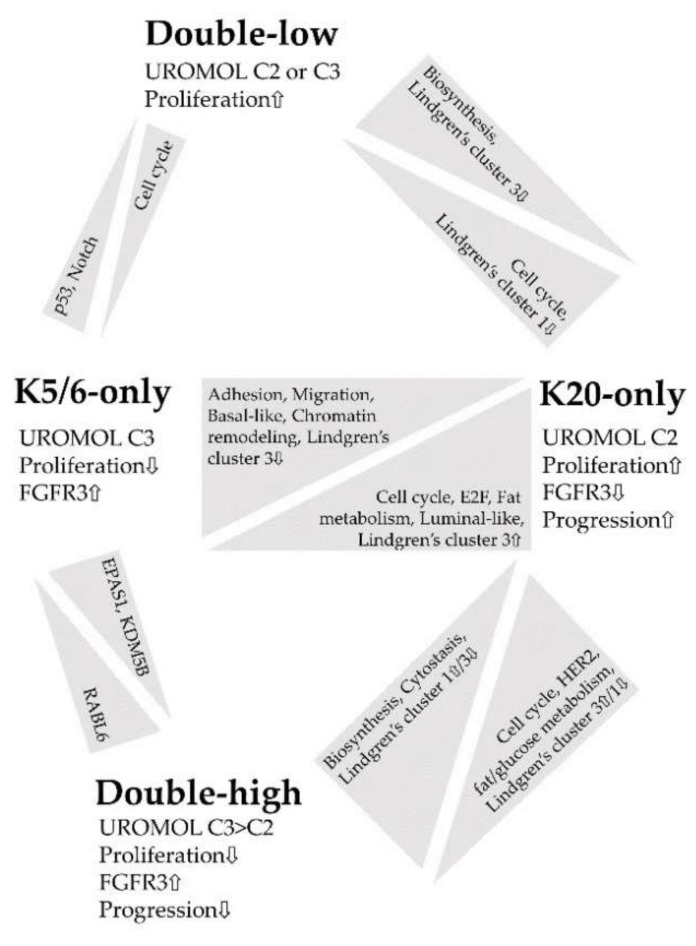
Summary of the functional enrichment of each comparison.

Figure 4 summarizes the major findings of this study. IHC staining for K5/6 and K20 is an indicator of molecular traits profoundly affected in NMIBC and closely associated with NMIBC subtypes [6,8]; thus, we propose K5/6 and K20 as promising biomarkers in the management of patients with NMIBC [16,28,43]. In particular, the K20-only group was significantly enriched in genes related to cell cycle, proliferation, and progression, which indicates a need for close observation of patients with NMIBC with luminal-like expression.

## Figures and Tables

**Table 1:** Significant functional enrichment predicted by Ingenuity Pathway Analysis.

Category	Predicted Result	Upregulated Group	z-Score ^1^	FDR ^2^
K20-only vs. K5/6-only group				
Upstream	RABL6	K20-only	2.530	5.9 × 10^−4^
Upstream	ID2	K20-only	2.208	5.5 × 10^−3^
Upstream	EFNA4	K20-only	2.449	1.9 × 10^−2^
Upstream	PPARGC1A	K20-only	3.125	1.9 × 10^−2^
Upstream	EFNA3	K20-only	2.449	1.9 × 10^−2^
Upstream	EFNA5	K20-only	2.449	2.2 × 10^−2^
Upstream	TP53	K5/6-only	2.752	1.0 × 10^−7^
Upstream	TP63	K5/6-only	2.213	3.3 × 10^−6^
Upstream	TGFB1	K5/6-only	4.169	2.3 × 10^−5^
Upstream	PGR	K5/6-only	2.288	8.8 × 10^−5^
Upstream	CDKN2A	K5/6-only	2.701	2.8 × 10^−4^
Upstream	KDM5B	K5/6-only	3.143	3.3 × 10^−4^
Upstream	BRCA1	K5/6-only	2.063	3.7 × 10^−4^
Upstream	RAF1	K5/6-only	3.385	3.7 × 10^−4^
Upstream	CDKN1A	K5/6-only	2.408	4.6 × 10^−4^
Upstream	RB1	K5/6-only	2.134	6.8 × 10^−4^
Upstream	SMARCA4	K5/6-only	2.959	7.6 × 10^−4^
Upstream	NUPR1	K5/6-only	3.413	8.5 × 10^−4^
Upstream	IKBKB	K5/6-only	2.016	1.7 × 10^−3^
Upstream	EGF	K5/6-only	2.089	2.0 × 10^−3^
Upstream	HIF1A	K5/6-only	2.059	3.2 × 10^−3^
Upstream	TGF beta	K5/6-only	2.288	3.4 × 10^−3^
Upstream	FSHB	K5/6-only	2.190	6.4 × 10^−3^
Upstream	FGF2	K5/6-only	3.083	8.0 × 10^−3^
Upstream	JAG1	K5/6-only	2.646	8.1 × 10^−3^
Upstream	PDGF BB	K5/6-only	2.652	8.9 × 10^−3^
Upstream	IGF1	K5/6-only	2.083	9.8 × 10^−3^
Upstream	CG	K5/6-only	2.332	1.2 × 10^−2^
Upstream	JUN	K5/6-only	2.502	1.3 × 10^−2^
Upstream	BNIP3L	K5/6-only	2.630	1.9 × 10^−2^
Upstream	CTNNB1	K5/6-only	2.630	2.3 × 10^−2^
Upstream	Calcineurin A	K5/6-only	2.200	2.9 × 10^−2^
Upstream	EDN1	K5/6-only	2.439	3.6 × 10^−2^
Upstream	ERK1/2	K5/6-only	2.163	3.7 × 10^−2^
Upstream	TGFB3	K5/6-only	2.926	3.8 × 10^−2^
Upstream	STAT3	K5/6-only	2.209	3.8 × 10^−2^
Disease/function	Endocrine gland tumor	K20-only	2.213	3.1 × 10^−8^
Disease/function	Congenital anomaly of digit	K20-only	2.000	7.3 × 10^−3^
Disease/function	Invasion of tumor cell lines	K5/6-only	2.069	1.3 × 10^−5^
Disease/function	Migration of cells	K5/6-only	2.102	3.4 × 10^−5^
Disease/function	Cell movement	K5/6-only	2.591	4.5 × 10^−5^
Disease/function	Adhesion of tumor cell lines	K5/6-only	2.481	7.4 × 10^−4^
Disease/function	Binding of tumor cell lines	K5/6-only	2.444	8.7 × 10^−4^
Disease/function	Attachment of cells	K5/6-only	2.040	1.5 × 10^−3^
Disease/function	Invasion of breast cancer cell lines	K5/6-only	2.015	1.5 × 10^−3^
Disease/function	Cell movement of breast cancer cell lines	K5/6-only	2.014	4.1 × 10^−3^
Disease/function	Apoptosis of prostate cancer cell lines	K5/6-only	3.467	5.9 × 10^−3^
Disease/function	Formation of gamma H2AX nuclear focus	K5/6-only	2.345	6.1 × 10^−3^
Disease/function	Cell movement of endothelial cells	K5/6-only	2.190	6.3 × 10^−3^
Disease/function	Apoptosis of cancer cells	K5/6-only	2.420	7.0 × 10^−3^
Disease/function	Necrosis of tumor	K5/6-only	2.495	7.7 × 10^−3^
Disease/function	Necrosis of prostate cancer cell lines	K5/6-only	3.223	8.2 × 10^−3^
K20-only vs. double-high group				
Upstream	ERBB2	K20-only	3.284	7.2 × 10^−4^
Upstream	EP400	K20-only	2.449	1.8 × 10^−3^
Upstream	E2f	K20-only	2.199	2.8 × 10^−3^
Upstream	RABL6	K20-only	3.000	5.6 × 10^−3^
Upstream	ID2	K20-only	2.563	5.6 × 10^−3^
Upstream	ID3	K20-only	2.157	2.1 × 10^−2^
Upstream	E2F3	K20-only	2.534	2.1 × 10^−2^
Upstream	MITF	K20-only	2.575	3.0 × 10^−2^
Upstream	SREBF2	K20-only	2.557	4.7 × 10^−2^
Upstream	TP53	Double-high	3.069	2.8 × 10^−8^
Upstream	CDKN2A	Double-high	4.097	3.7 × 10^−4^
Upstream	CDKN1A	Double-high	2.729	4.5 × 10^−4^
Upstream	MLXIPL	Double-high	3.592	1.4 × 10^−3^
Upstream	OGA	Double-high	3.272	2.5 × 10^−3^
Upstream	MYCN	Double-high	2.385	8.1 × 10^−3^
Disease/function	DNA replication	K20-only	2.159	8.8 × 10^−5^
Disease/function	Genitourinary adenocarcinoma	K20-only	2.177	1.0 × 10^−4^
Disease/function	Proliferation of connective tissue cells	K20-only	3.115	4.2 × 10^−4^
Disease/function	Growth of connective tissue	K20-only	2.790	5.5 × 10^−4^
Disease/function	Cell proliferation of breast cancer cell lines	K20-only	2.032	2.6 × 10^−3^
Disease/function	Advanced malignant solid tumor	K20-only	2.228	3.6 × 10^−3^
Disease/function	Growth of organism	K20-only	2.299	6.0 × 10^−3^
Disease/function	Advanced lung cancer	K20-only	2.578	6.4 × 10^−3^
Disease/function	Cell cycle progression of tumor cell lines	K20-only	2.395	1.1 × 10^−2^
Disease/function	Visceral metastasis	K20-only	2.594	1.3 × 10^−2^
Disease/function	Metastatic solid tumor	K20-only	2.228	1.3 × 10^−2^
Disease/function	Advanced extracranial solid tumor	K20-only	2.576	1.8 × 10^−2^
Disease/function	Cell death of breast cancer cell lines	Double-high	2.359	2.7 × 10^−5^
Disease/function	Cell death of tumor cell lines	Double-high	3.092	4.4 × 10^−5^
Disease/function	Apoptosis	Double-high	2.128	2.1 × 10^−4^
Disease/function	Gastrointestinal tract cancer	Double-high	2.000	1.1 × 10^−3^
Disease/function	Senescence of cells	Double-high	2.104	1.1 × 10^−3^
Disease/function	Cell death of lung cancer cell lines	Double-high	2.444	3.7× 10^−3^
Disease/function	Colon tumor	Double-high	2.364	7.1 × 10^−3^
Disease/function	Proliferation of hematopoietic progenitor cells	Double-high	2.005	1.6 × 10^−2^
Disease/function	Colorectal tumor	Double-high	2.078	1.6 × 10^−2^
Disease/function	Cytostasis of tumor cell lines	Double-high	2.145	1.8 × 10^−2^
K5/6-only vs. double-high group				
Upstream	EPAS1	K5/6-only	2.000	3.9 × 10^−2^
Disease/function	NA	NA	NA	NA
K20-only vs. double-low group				
Upstream	ID3	K20-only	2.213	1.2 × 10^−2^
Upstream	MYC	K20-only	2.299	1.3 × 10^−2^
Upstream	LLGL2	K20-only	2.000	4.4 × 10^−2^
Upstream	ERBB3	K20-only	2.588	4.7 × 10^−2^
Disease/function	Genitourinary tumor	K20-only	2.017	2.1 × 10^−3^
Disease/function	Malignant genitourinary solid tumor	K20-only	2.139	2.1 × 10^−3^
Disease/function	Adenocarcinoma	K20-only	2.204	3.2 × 10^−3^
Disease/function	Anogenital cancer	K20-only	2.144	3.9 × 10^−3^
Disease/function	Incidence of tumor	K20-only	2.257	7.0 × 10^−3^
Disease/function	Carcinoma	K20-only	2.292	7.8 × 10^−3^
Disease/function	Extracranial solid tumor	K20-only	2.697	1.4 × 10^−2^
Disease/function	Malignant solid tumor	K20-only	2.026	2.3 × 10^−2^
Disease/function	Epithelial–mesenchymal transition of breast cell lines	K20-only	2.108	2.8 × 10^−2^
K5/6-only vs. double-low group				
Upstream	TP53	K5/6-only	2.086	1.8 × 10^−4^
Disease/function	NA	NA	NA	NA

^1^ z-score indicates an activation level of the predicted result based on the DEG fold change and its agreement with the result. ^2^ FDR, false discovery rate (Benjamini Hochberg).

**Table 2:** Significant functional enrichment of the Lund groups predicted by Ingenuity Pathway Analysis.

Category	Predicted Result	Upregulated Group	z-Score ^1^	FDR ^2^
K20-only_Lund vs. K5/6-only_Lund group				
Upstream	RABL6	K20-only	5.014	2.4 × 10^−27^
Upstream	ERBB2	K20-only	4.092	5.9 × 10^−24^
Upstream	FOXM1	K20-only	3.818	1.7 × 10^−16^
Upstream	MITF	K20-only	3.962	7.6 × 10^−15^
Upstream	FOXO1	K20-only	2.493	6.7 × 10^−12^
Upstream	LIN9	K20-only	3.130	3.6 × 10^−11^
Upstream	AREG	K20-only	3.195	1.3 × 10^−9^
Upstream	E2F3	K20-only	3.592	6.1 × 10^−8^
Upstream	E2f	K20-only	2.449	1.4 × 10^−7^
Upstream	MYBL2	K20-only	2.607	3.1 × 10^−7^
Upstream	ELAVL1	K20-only	3.278	2.3 × 10^−5^
Upstream	HSPB1	K20-only	2.429	3.1 × 10^−5^
Upstream	RARA	K20-only	4.000	5.4 × 10^−5^
Upstream	TAL1	K20-only	3.000	5.6 × 10^−5^
Upstream	KDM1A	K20-only	3.434	7.1 × 10^−5^
Upstream	ESR1	K20-only	3.601	1.0 × 10^−4^
Upstream	26s Proteasome	K20-only	2.357	3.5 × 10^−4^
Upstream	BRD4	K20-only	2.603	3.9 × 10^−4^
Upstream	TRAF2	K20-only	2.224	6.7 × 10^−4^
Upstream	NSUN6	K20-only	2.449	4.3 × 10^−3^
Upstream	S100A6	K20-only	2.236	6.9 × 10^−3^
Upstream	CREB1	K20-only	2.219	1.5 × 10^−2^
Upstream	TP53	K5/6-only	5.972	1.4 × 10^−14^
Upstream	TRPS1	K5/6-only	3.742	3.4 × 10^−14^
Upstream	NUPR1	K5/6-only	2.592	6.7 × 10^−12^
Upstream	CDKN1A	K5/6-only	2.783	1.4 × 10^−7^
Upstream	KDM5B	K5/6-only	3.487	1.5 × 10^−7^
Upstream	E2F6	K5/6-only	2.236	4.3 × 10^−4^
Upstream	ATF3	K5/6-only	2.369	6.0 × 10^−4^
Upstream	CTLA4	K5/6-only	2.236	5.1 × 10^−3^
Upstream	CDKN2A	K5/6-only	2.433	3.4 × 10^−2^
Disease/function	M phase	K20-only	2.142	1.2x10^−12^
Disease/function	Alignment of chromosomes	K20-only	2.324	7.2 × 10^−12^
Disease/function	M phase of tumor cell lines	K20-only	2.613	5.2 × 10^−10^
Disease/function	Cytokinesis	K20-only	2.278	2.8 × 10^−8^
Disease/function	M phase of cervical cancer cell lines	K20-only	2.019	8.5 × 10^−8^
Disease/function	Cytokinesis of tumor cell lines	K20-only	2.249	1.0 × 10^−7^
Disease/function	Interphase	K20-only	2.744	2.4 × 10^−7^
Disease/function	Cell survival	K20-only	2.348	6.7 × 10^−6^
Disease/function	Cell viability of tumor cell lines	K20-only	2.470	1.3 × 10^−5^
Disease/function	Cell proliferation of tumor cell lines	K20-only	2.796	5.4 × 10^−5^
Disease/function	Cell viability	K20-only	2.328	9.8 × 1^−5^
Disease/function	G1 phase	K20-only	2.111	1.1 × 10^−4^
Disease/function	Mitotic index	K20-only	2.214	5.8 × 10^−4^
Disease/function	Cell viability of myeloma cell lines	K20-only	2.601	3.7 × 10^−3^
Disease/function	Interphase of tumor cell lines	K20-only	2.017	6.0 × 10^−3^
Disease/function	Interphase of cervical cancer cell lines	K20-only	2.392	1.8 × 10^−2^
Disease/function	Cell viability of lung cancer cell lines	K20-only	2.100	4.7 × 10^−2^
Disease/function	Missegregation of chromosomes	K5/6-only	2.392	1.4 × 10^−3^
K20-only_Lund vs. double-high_Lund group				
Upstream	RABL6	K20-only	2.828	4.2 × 10^−4^
Upstream	ERBB2	K20-only	2.219	1.2 × 10^−2^
Upstream	TGFB1	K20-only	2.186	4.3 × 10^−2^
Disease/function	NA	NA	NA	NA
K5/6-only_Lund vs. double-high_Lund group				
Upstream	KDM5B	K5/6-only	2.000	8.6 × 10^−3^
Upstream	RABL6	Double-high	2.236	2.2 × 10^−4^
Disease/function	NA	NA	NA	NA
K20-only_Lund vs. double-low_Lund group				
Upstream	RABL6	K20-only	3.207	6.4 × 10^−10^
Upstream	FOXM1	K20-only	3.382	7.1 × 10^−8^
Upstream	ERBB2	K20-only	2.538	7.1 × 10^−7^
Upstream	MYBL2	K20-only	2.412	2.3 × 10^−6^
Upstream	LIN9	K20-only	2.438	7.0 × 10^−6^
Upstream	AREG	K20-only	2.132	2.9 × 10^−5^
Upstream	HIF1A-AS1	K20-only	2.236	3.6 × 10^−4^
Upstream	26s Proteasome	K20-only	2.607	2.9 × 10^−3^
Upstream	E2F3	K20-only	2.646	7.3 × 10^−3^
Upstream	RARA	K20-only	3.000	2.1 × 10^−2^
Upstream	MITF	K20-only	2.646	2.4 × 10^−2^
Upstream	ESR1	K20-only	2.718	4.8 × 10^−2^
Upstream	TRPS1	Double-low	2.828	7.1 × 10^−7^
Upstream	TP53	Double-low	3.827	3.2 × 10^−6^
Upstream	CDKN1A	Double-low	2.848	2.9 × 10^−5^
Upstream	CTLA4	Double-low	2.236	1.3x10^−3^
Upstream	KDM5B	Double-low	2.823	5.3 × 10^−3^
Disease/function	Segregation of chromosomes	K20-only	2.000	4.9 × 10^−4^
K5/6-only_Lund vs. double-low_Lund group				
Upstream	NA	NA	NA	NA
Disease/function	NA	NA	NA	NA

^1^ z-score indicates an activation level of the predicted result based on the DEG fold change and its agreement with the result. ^2^ FDR, false discovery rate (Benjamini Hochberg).

## Supplementary Materials

Supplementary Materials can be found at https://www.mdpi.com/1422-0067/21/20/7726/s1. Figure S1: Representative microscopic images of IHC staining for K5/6 and K20; Figure S2: Kaplan-Meier and log-rank tests of relapse-free survival of the groups; Figure S3: Identification of DEGs between the groups; Figure S4: Cell proliferation functions significantly enriched in the groups identified by the GSEA; Figure S5: Metabolism functions significantly enriched in the groups identified by the GSEA; Figure S6: Gene expression characteristics of basal vs. luminal cells of the breast significantly enriched in the groups identified by the GSEA; Figure S7: Identification of DEGs between the Lund cohort groups; Figure S8: *KRT* gene expression levels of each group; Figure S9: Accuracy of prediction models validated using gene-expression classifier of UROMOL NMIBC subtypes; Table S1: Clinicopathological details of the groups; Table S2: GO and KEGG analyses of the groups; Table S3: Class annotation to the UROMOL intrinsic subtypes by machine learning; Table S4: Clinicopathological details of the Lund cohort groups; Table S5: GO and KEGG analyses of the Lund cohort.

## Abbreviations

NMIBC	Non-muscle-invasive bladder cancer
MIBC	Muscle-invasive bladder cancer
IHC	Immunohistochemical
NMIUTUC	Non-muscle-invasive upper tract urothelial carcinoma
MAPK	Mitogen-activated protein kinase
DEG	Differentially expressed gene
GO	Gene ontology
KEGG	Kyoto Encyclopedia of Genes and Genomes
IPA	Ingenuity Pathway Analysis
GSEA	Gene Set Enrichment Analysis
FDR	False discovery rate
