# Supplementary material for: Non-Muscle-Invasive Bladder Carcinoma with Respect to Basal Versus Luminal Keratin Expression
Source: Int J Mol Sci. 2020 Oct 19;21(20):7726. doi: 10.3390/ijms21207726 (PMC7589917; doi:10.3390/ijms21207726)
Supplement: Supplementary file 1 [file ijms-21-07726-s001.pdf]

International Journal of *Molecular Sciences*

Supplementary Information

Non-Muscle-Invasive Bladder Carcinoma with Respect to Basal Versus Luminal Keratin Expression

Minsun Jung [1], Insoon Jang [2], Kwangsoo Kim [3], and Kyung Chul Moon [1,4,*]

[1] Department of Pathology, Seoul National University College of Medicine, Seoul 03080, Korea; jjunglammy@gmail.com
[2] Biomedical Research Institute, Seoul National University Hospital, Seoul 03080, Korea; isjang1324@gmail.com
[3] Transdisciplinary Department of Medicine & Advanced Technology, Seoul National University Hospital, Seoul 03080, Korea; kksoo716@gmail.com
[4] Kidney Research Institute, Medical Research Center, Seoul National University College of Medicine, Seoul 03080, Korea
* Correspondence: blue7270@gmail.com; Tel.: +82-2-740-8380

Received: 22 September 2020; Accepted: 13 October 2020; Published: date

1. Figures S1–S9
2. Tables S1–S5

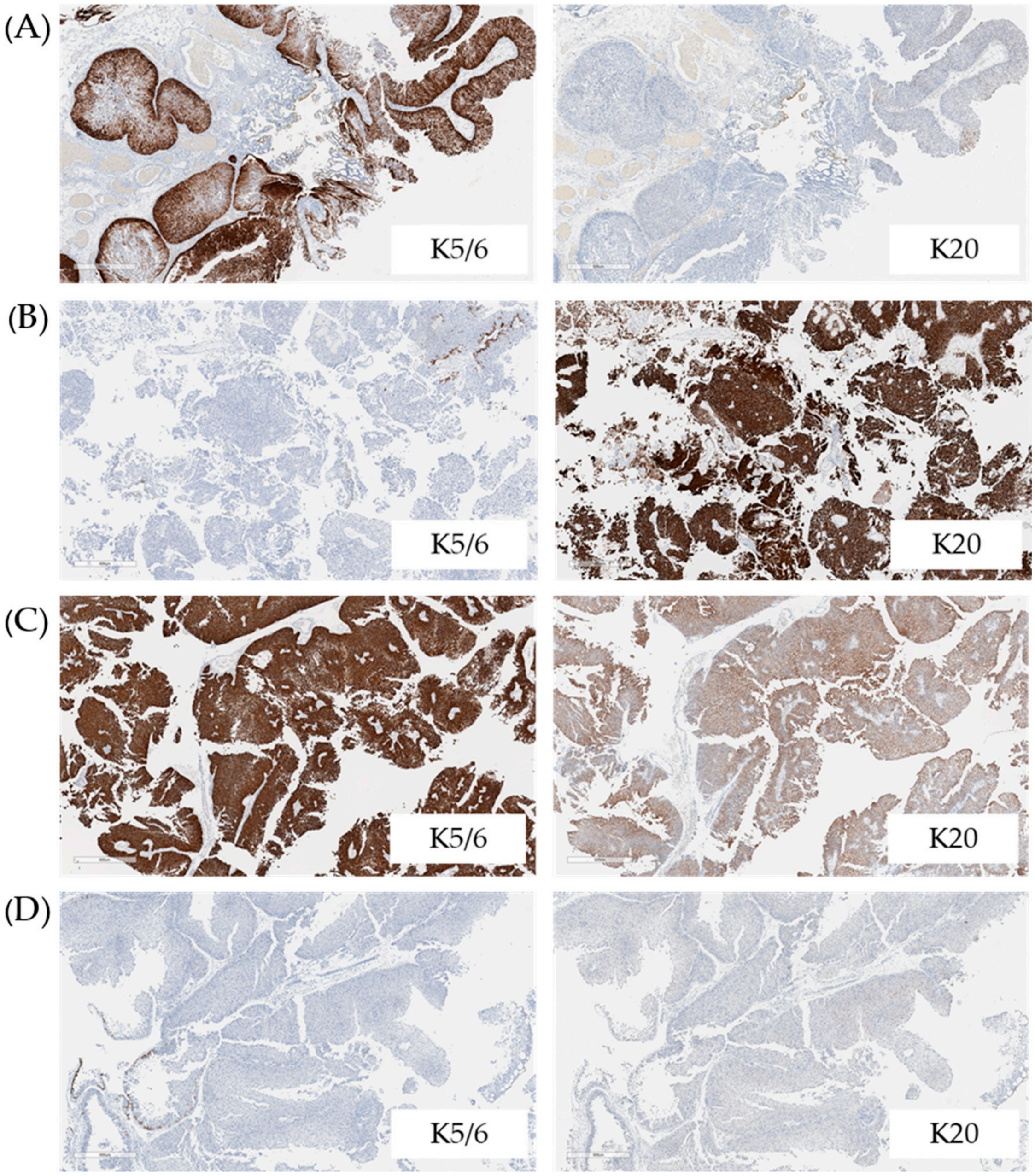

Figure S1. Representative microscopic images of IHC staining for K5/6 and K20. (**a**) K5/6-only group. (**b**) K20-only group. (**c**) Double-high group. (**d**) Double-low group.

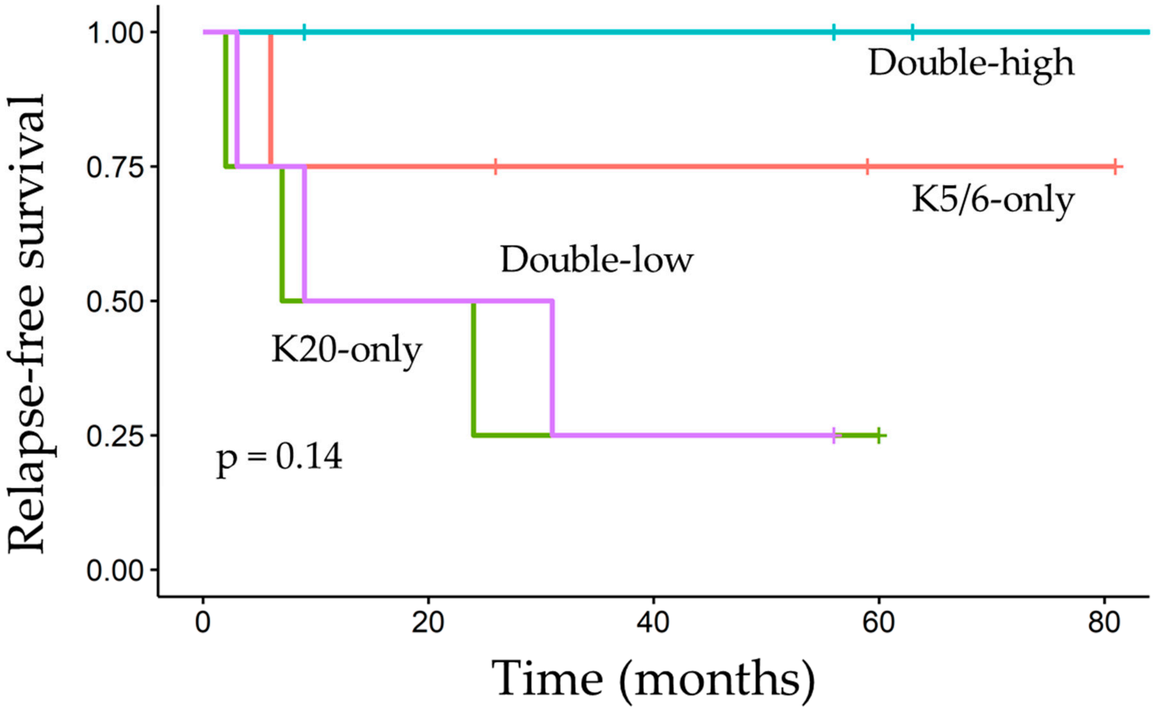

Figure S2. Kaplan-Meier and log-rank tests of relapse-free survival of the groups.

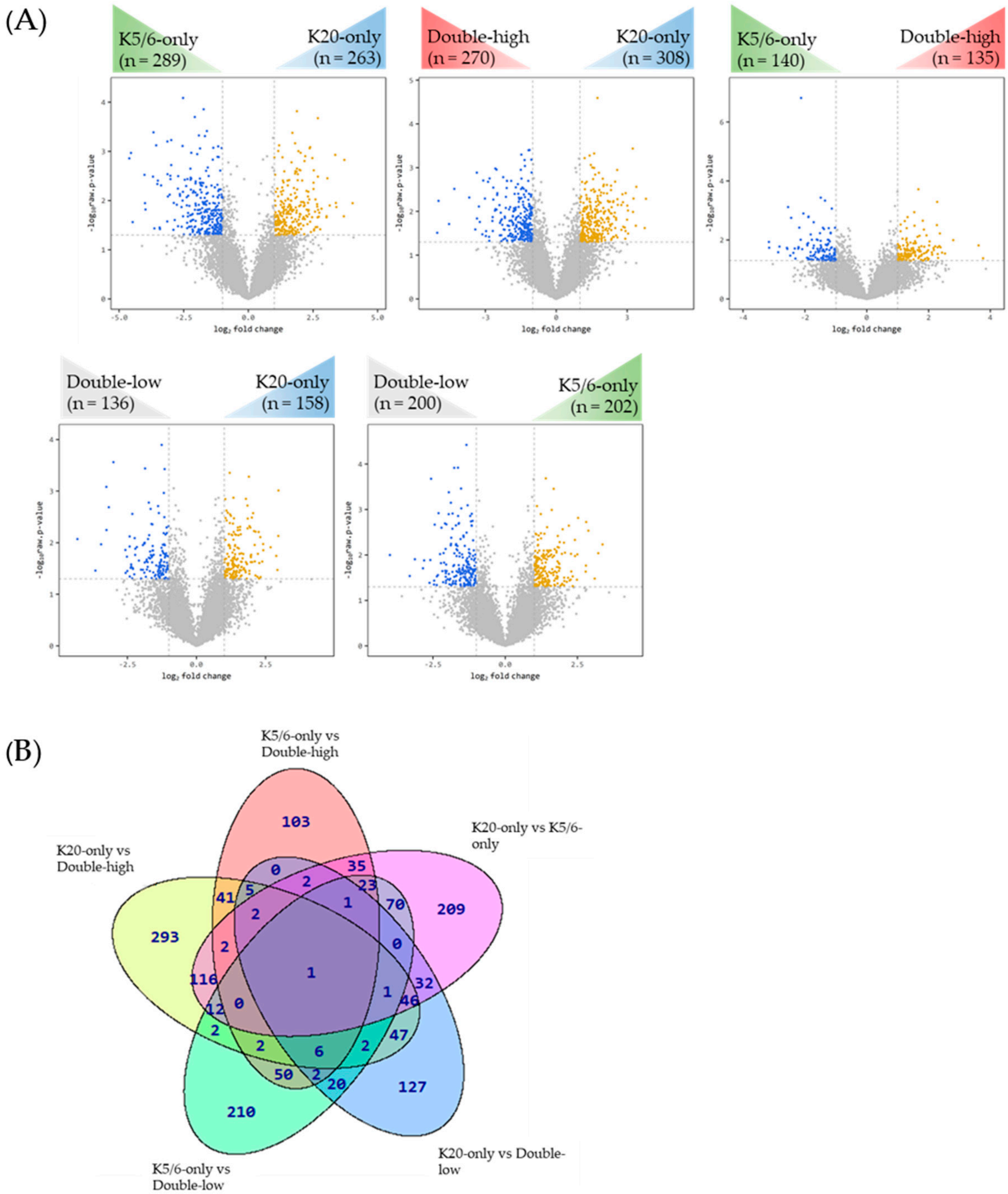

Figure S3. Identification of DEGs between the groups. (**a**) Numbers of DEGs. (**b**) Venn diagram of DEGs.

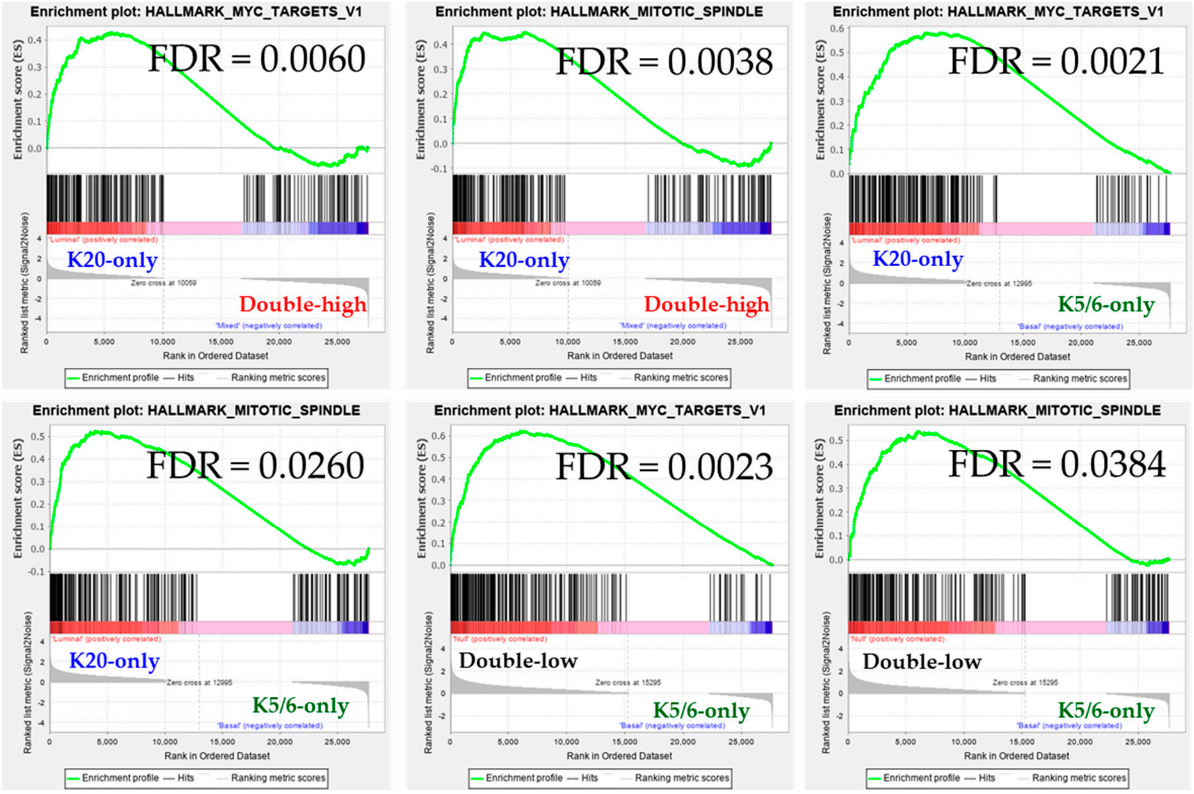

Figure S4. Cell proliferation functions significantly enriched in the groups identified by the GSEA.

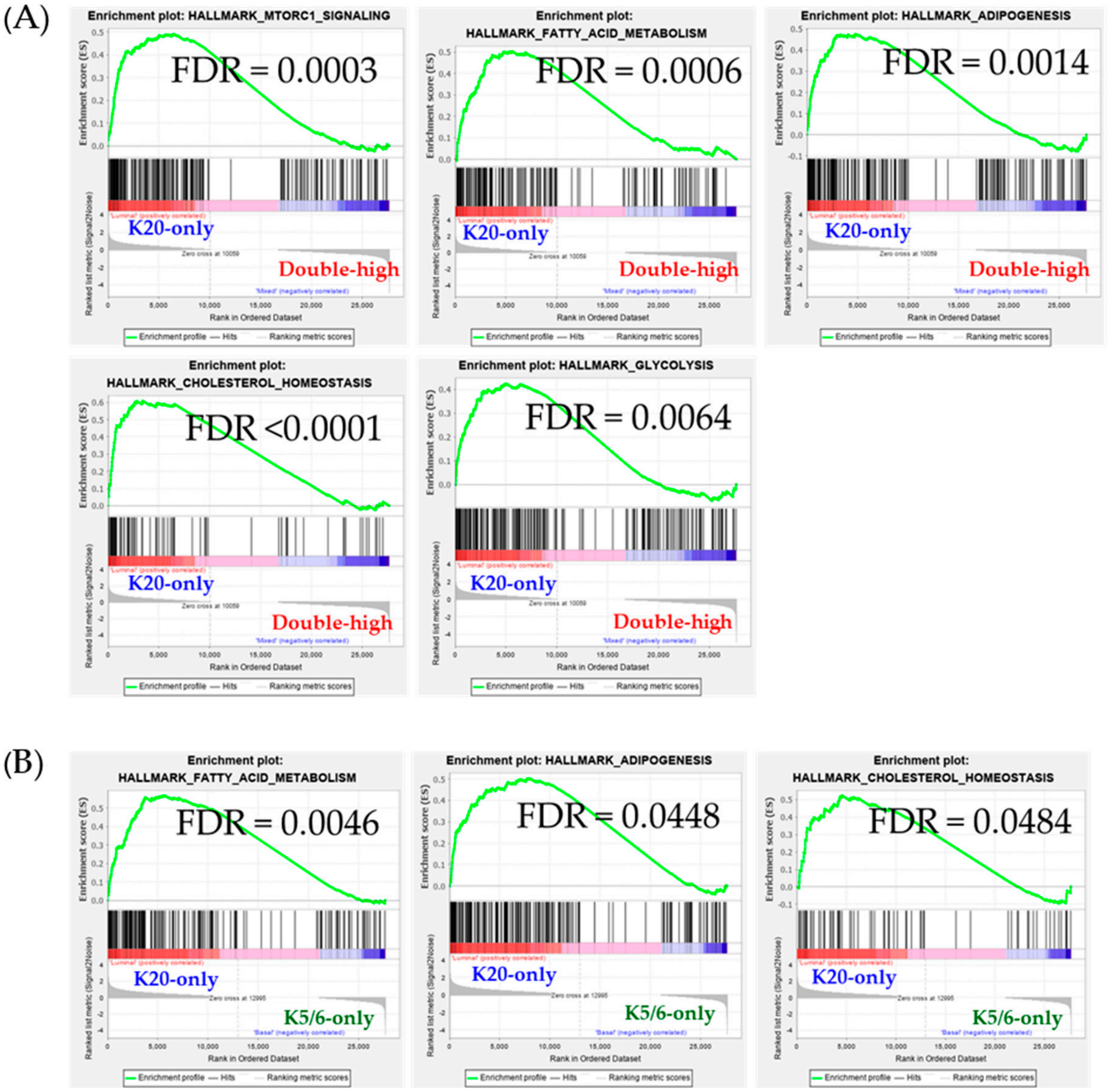

Figure S5. Metabolism functions significantly enriched in the groups identified by the GSEA. (**a**) Comparison between the K20-only and double-high groups. (**b**) Comparison between the K20-only and K5/6-only groups.

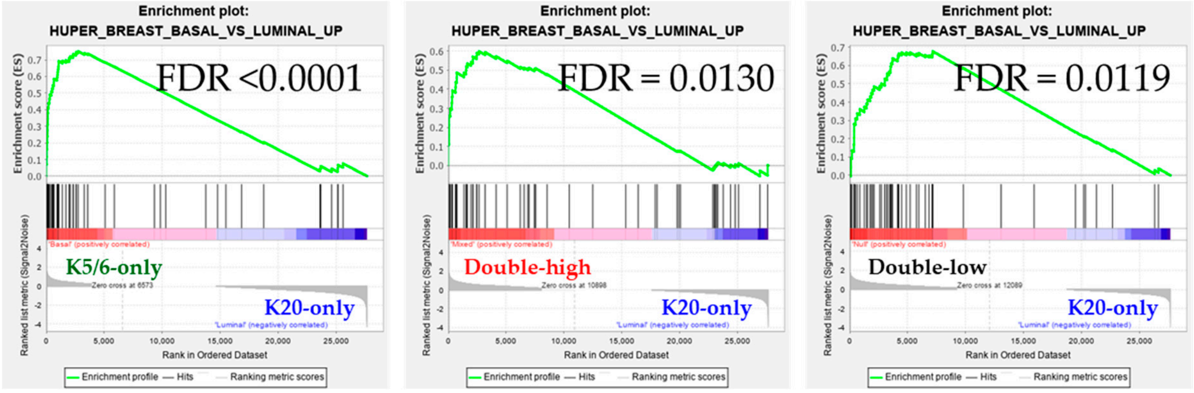

Figure S6. Gene expression characteristics of basal vs. luminal cells of the breast significantly enriched in the groups identified by the GSEA.

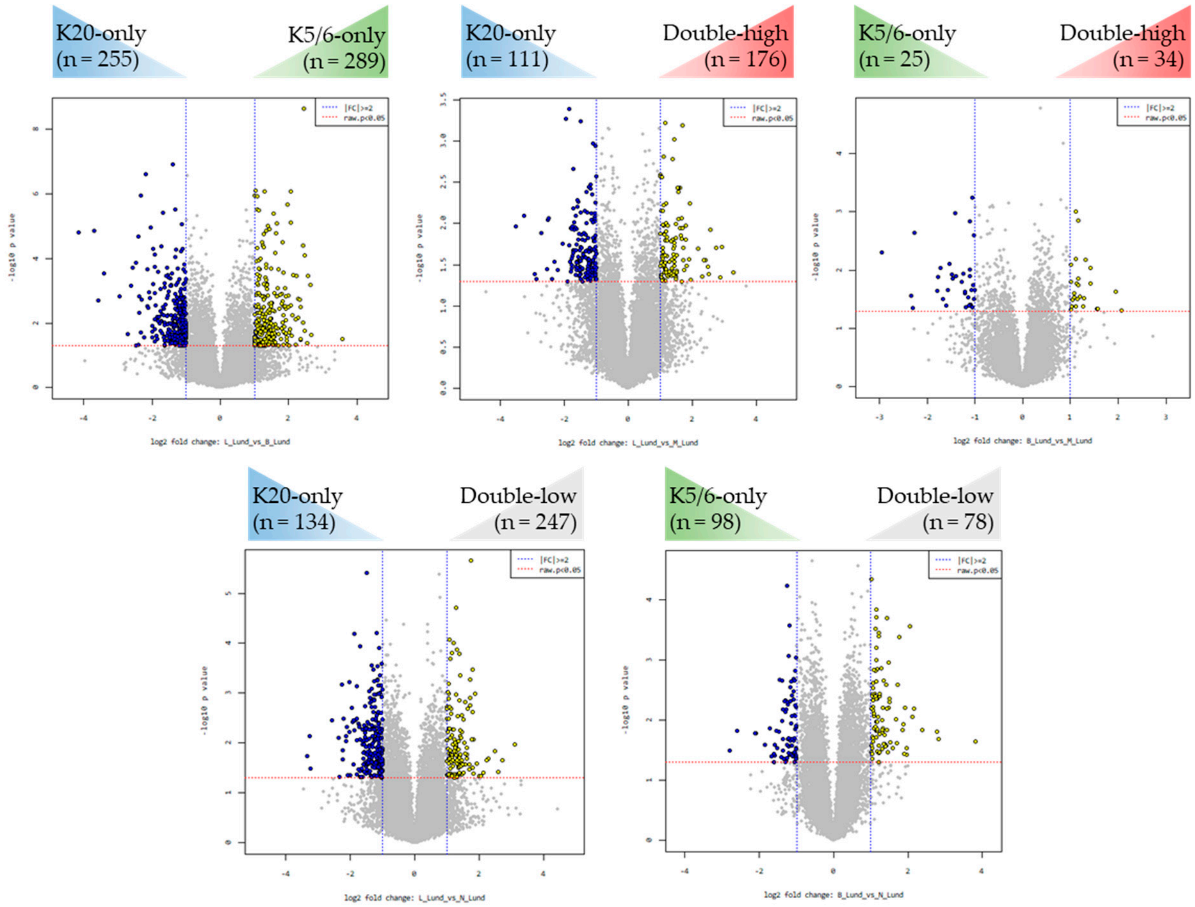

Figure S7. Identification of DEGs between the Lund cohort groups.

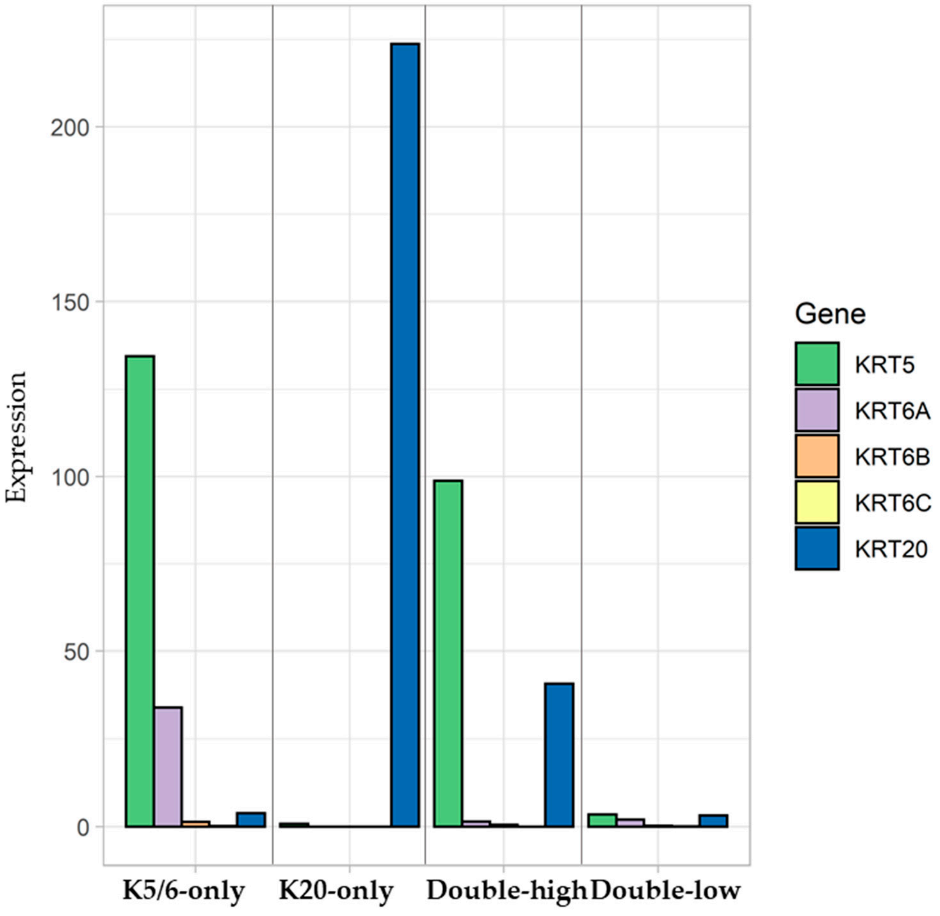

Figure S8. *KRT* gene expression levels of each group.

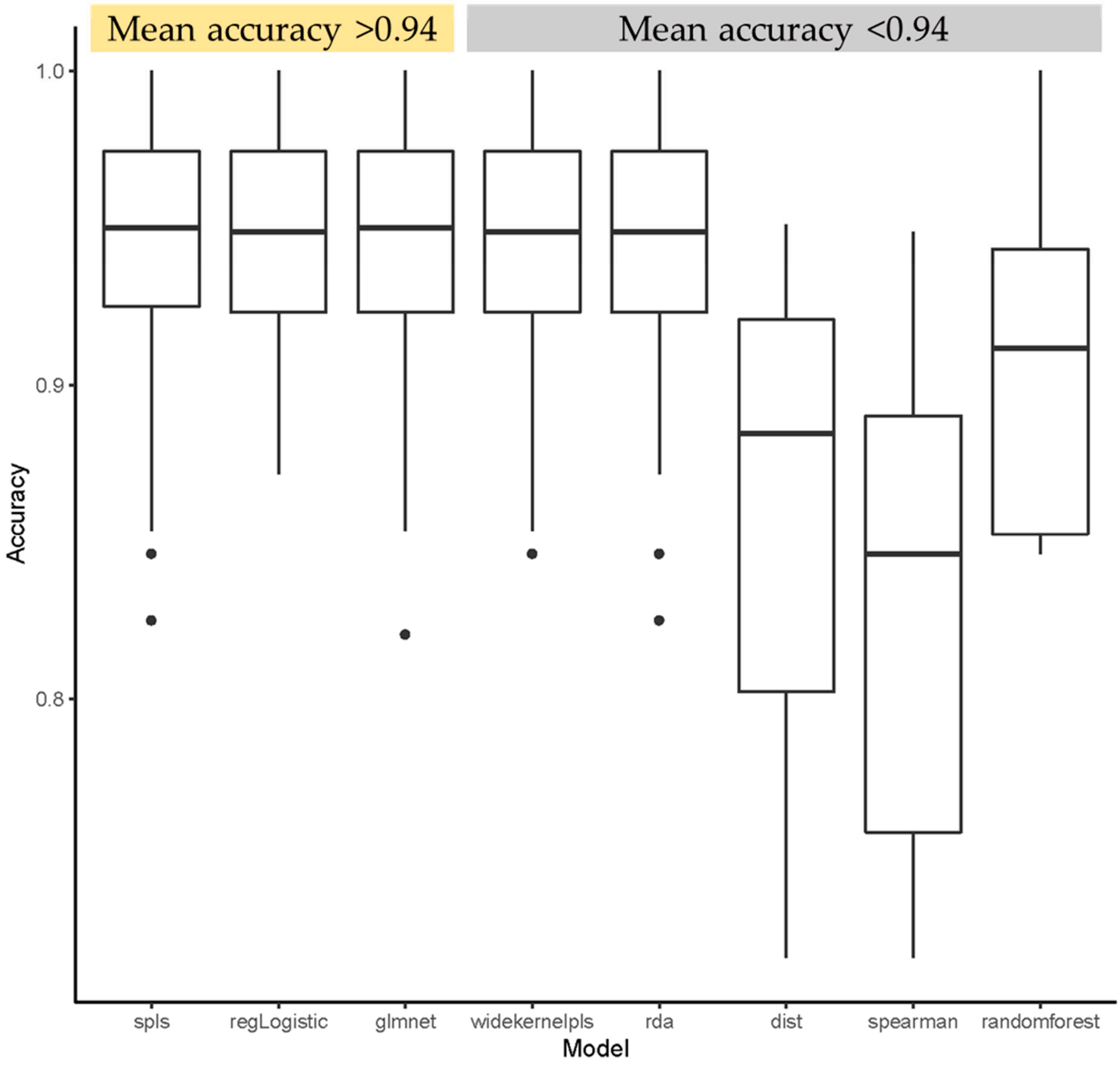

Figure S9. Accuracy of prediction models validated using gene-expression classifier of UROMOL NMIBC subtypes.

Table S1. Clinicopathological details of the groups

Group	K5/6-only (n = 4)	K20-only (n = 4)	Double-high (n = 4)	Double-low (n = 4)	p[a]
Age, median (range)	65 years (63-76)	72 years (67-83)	68 years (58-73)	72 years (63-83)	0.486
Sex					0.543
Female	0 (0.0%)	1 (25.0%)	2 (50.0%)	0 (0.0%)	
Male	4 (100.0%)	3 (75.0%)	2 (50.0%)	4 (100.0%)	
Stromal invasion (T stage)					0.859
Present (T1)	4 (100.0%)	3 (75.0%)	2 (50.0%)	3 (75.0%)	
Absent (Ta)	0 (0.0%)	1 (25.0%)	2 (50.0%)	1 (25.0%)	
WHO grade					1.000
High	4 (100.0%)	4 (100.0%)	4 (100.0%)	4 (100.0%)	
Low	0 (0.0%)	0 (0.0%)	0 (0.0%)	0 (0.0%)	
Carcinoma *in situ*					1.000
Present	0 (0.0%)	1 (25.0%)	0 (0.0%)	1 (25.0%)	
Absent	4 (100.0%)	3 (75.0%)	4 (100.0%)	3 (75.0%)	
BCG after resection					1.000
Yes	2 (50.0%)	3 (75.0%)	1 (25.0%)	1 (25.0%)	
No	2 (50.0%)	1 (25.0%)	3 (75.0%)	3 (75.0%)	
DV200 (mean ± S.D.)	48.0 ± 18.6%	68.2 ± 6.0%	59.6 ± 9.0%	71.5 ± 15.4%	0.100
Immunohistochemistry score					
K5/6 (mean ± S.D.)	5.8 ± 0.5	0.0 ± 0.0	4.5 ± 1.0	1.2 ± 0.5	0.003
K20 (mean ± S.D.)	2.5 ± 0.6	5.8 ± 0.5	4.8 ± 1.0	1.5 ± 0.6	0.004
K14 (mean ± S.D.)	1.8 ± 1.3	0.0 ± 0.0	0.8 ± 1.0	0.2 ± 0.5	0.099
GATA3 (mean ± S.D.)	6.0 ± 0.0	6.0 ± 0.0	6.0 ± 0.0	5.8 ± 0.5	0.426
FOXA1 (mean ± S.D.)	5.8 ± 0.5	5.8 ± 0.5	6.0 ± 0.0	6.0 ± 0.0	0.646
p53					0.333
Overexpression	0 (0.0%)	3 (75.0%)	2 (50.0%)	1 (25.0%)	
No overexpression	4 (100.0%)	1 (25.0%)	2 (50.0%)	3 (75.0%)	

[a]: Pearson's chi-squared or Fisher's exact tests for categorical variables; Mann-Whitney U test for immunohistochemistry

Table S2. GO and KEGG analyses of the groups.

**K20-only vs K5/6-only**				
Upregulated in the K20-only group	Source	Terms	FDR	Term ID
	GO:CC	intracellular	1.19865E-08	GO:0005622
	GO:CC	organelle	1.19865E-08	GO:0043226
	GO:CC	intracellular organelle	1.04288E-07	GO:0043229
	GO:CC	intracellular membrane-bounded organelle	1.79728E-07	GO:0043231
	GO:CC	membrane-bounded organelle	2.00806E-07	GO:0043227
	GO:CC	nuclear outer membrane-endoplasmic reticulum membrane network	4.44075E-05	GO:0042175
	GO:CC	cytoplasm	4.44075E-05	GO:0005737
	GO:CC	endoplasmic reticulum membrane	6.08089E-05	GO:0005789
	GO:CC	condensed chromosome	8.66986E-05	GO:0000793
	GO:CC	organelle membrane	9.0727E-05	GO:0031090
	GO:CC	condensed chromosome, centromeric region	0.000238973	GO:0000779
	GO:CC	endomembrane system	0.000248882	GO:0012505
	GO:CC	condensed chromosome kinetochore	0.000484199	GO:0000777
	GO:CC	cytosol	0.000484199	GO:0005829
	GO:CC	kinetochore	0.00059818	GO:0000776
	GO:CC	chromosome	0.000916236	GO:0005694
	GO:CC	endoplasmic reticulum	0.002024112	GO:0005783
	GO:CC	chromosome, centromeric region	0.002189696	GO:0000775
	GO:CC	intracellular non-membrane-bounded organelle	0.0030623	GO:0043232
	GO:CC	spindle	0.0030623	GO:0005819
	GO:CC	non-membrane-bounded organelle	0.003152998	GO:0043228
	GO:CC	organelle envelope	0.004304358	GO:0031967
	GO:CC	envelope	0.004304358	GO:0031975
	GO:BP	cell cycle	0.004867688	GO:0007049
	GO:CC	chromosomal region	0.006471104	GO:0098687
	GO:CC	host cell	0.009640099	GO:0043657
	GO:CC	host cellular component	0.009640099	GO:0018995
	GO:BP	cellular response to stress	0.011353697	GO:0033554
	GO:BP	mitotic cell cycle	0.011353697	GO:0000278
	GO:BP	double-strand break repair via homologous recombination	0.011353697	GO:0000724
	GO:BP	recombinational repair	0.011353697	GO:0000725
	GO:BP	cell division	0.011353697	GO:0051301
	GO:BP	mitotic cell cycle process	0.011353697	GO:1903047
	GO:BP	double-strand break repair	0.011353697	GO:0006302
	GO:BP	cell cycle process	0.011353697	GO:0022402
	GO:CC	nuclear envelope	0.013402502	GO:0005635
	GO:CC	nuclear membrane	0.013780238	GO:0031965
	GO:CC	nucleus	0.014086337	GO:0005634
	GO:CC	dendrite cytoplasm	0.014086337	GO:0032839

	GO:CC	condensed nuclear chromosome outer kinetochore	0.016947953	GO:0000942
	GO:BP	mRNA transport	0.017404477	GO:0051028
	GO:BP	nucleobase-containing compound transport	0.017404477	GO:0015931
	GO:BP	regulation of cellular response to stress	0.019209402	GO:0080135
	GO:BP	DNA recombination	0.019209402	GO:0006310
	GO:CC	condensed nuclear chromosome kinetochore	0.021860164	GO:0000778
	GO:BP	organelle organization	0.022736335	GO:0006996
	GO:BP	establishment of RNA localization	0.023104496	GO:0051236
	GO:BP	nucleic acid transport	0.023104496	GO:0050657
	GO:BP	regulation of cellular response to heat	0.023104496	GO:1900034
	GO:BP	RNA localization	0.023104496	GO:0006403
	GO:BP	tRNA-containing ribonucleoprotein complex export from nucleus	0.023104496	GO:0071431
	GO:BP	RNA transport	0.023104496	GO:0050658
	GO:BP	tRNA export from nucleus	0.023104496	GO:0006409
	GO:BP	DNA repair	0.023104496	GO:0006281
	GO:BP	DNA replication	0.023445885	GO:0006260
	GO:BP	ncRNA export from nucleus	0.023709531	GO:0097064
	GO:BP	mRNA-containing ribonucleoprotein complex export from nucleus	0.023709531	GO:0071427
	GO:BP	organic substance transport	0.023709531	GO:0071702
	GO:BP	mRNA export from nucleus	0.023709531	GO:0006406
	GO:BP	DNA packaging	0.023709531	GO:0006323
	GO:BP	tRNA transport	0.023709531	GO:0051031
	GO:BP	DNA conformation change	0.023709531	GO:0071103
	GO:BP	protein localization to nuclear inner membrane	0.024480669	GO:0036228
	GO:CC	spindle pole	0.027215771	GO:0000922
	GO:MF	structural constituent of nuclear pore	0.0281312	GO:0017056
	GO:CC	microtubule cytoskeleton	0.035886019	GO:0015630
	GO:BP	oxoacid metabolic process	0.038722382	GO:0043436
	GO:BP	sister chromatid segregation	0.038722382	GO:0000819
	GO:BP	cellular localization	0.041189716	GO:0051641
	GO:BP	nuclear chromosome segregation	0.041189716	GO:0098813
	GO:BP	carboxylic acid metabolic process	0.043730223	GO:0019752
	GO:BP	organic acid metabolic process	0.046257357	GO:0006082
	GO:BP	ribonucleoprotein complex export from nucleus	0.046943103	GO:0071426
	GO:BP	ribonucleoprotein complex localization	0.046943103	GO:0071166
	GO:BP	cellular response to DNA damage stimulus	0.046943103	GO:0006974
	GO:BP	intracellular protein transport	0.046943103	GO:0006886
	KEGG	Metabolic pathways	1.60895E-07	01100
	KEGG	RNA transport	0.00783835	03013
	KEGG	Apoptosis	0.01418239	04210
	KEGG	Herpes simplex virus 1 infection	0.036386979	05168
	KEGG	Sphingolipid signaling pathway	0.036386979	04071

	KEGG	Platinum drug resistance	0.04013763	01524
	KEGG	DNA replication	0.048297864	03030
**Upregulated in the K5/6-only group**	**Source**	**Terms**	**FDR**	**Term ID**
	GO:BP	tissue development	5.33945E-06	GO:0009888
	GO:BP	anatomical structure morphogenesis	5.33945E-06	GO:0009653
	GO:BP	anatomical structure formation involved in morphogenesis	7.42966E-06	GO:0048646
	GO:BP	cell migration	1.39591E-05	GO:0016477
	GO:BP	tissue morphogenesis	3.49652E-05	GO:0048729
	GO:BP	gastrulation	4.84466E-05	GO:0007369
	GO:BP	locomotion	4.84466E-05	GO:0040011
	GO:BP	cell motility	4.84466E-05	GO:0048870
	GO:BP	localization of cell	4.84466E-05	GO:0051674
	GO:BP	regulation of multicellular organismal process	4.84466E-05	GO:0051239
	GO:BP	embryonic morphogenesis	5.90975E-05	GO:0048598
	GO:BP	formation of primary germ layer	9.22979E-05	GO:0001704
	GO:BP	movement of cell or subcellular component	9.22979E-05	GO:0006928
	GO:BP	cell adhesion	0.000100528	GO:0007155
	GO:BP	positive regulation of cellular protein metabolic process	0.000100808	GO:0032270
	GO:BP	biological adhesion	0.000100808	GO:0022610
	GO:BP	intracellular signal transduction	0.000113937	GO:0035556
	GO:BP	regulation of developmental process	0.000113937	GO:0050793
	GO:BP	regulation of cellular protein metabolic process	0.00013631	GO:0032268
	GO:CC	cell junction	0.000172843	GO:0030054
	GO:CC	anchoring junction	0.000172843	GO:0070161
	GO:BP	regulation of molecular function	0.000199508	GO:0065009
	GO:BP	system development	0.000230655	GO:0048731
	GO:MF	insulin-like growth factor I binding	0.000236269	GO:0031994
	GO:MF	MAP kinase tyrosine/serine/threonine phosphatase activity	0.000236269	GO:0017017
	GO:BP	epithelium development	0.000246989	GO:0060429
	GO:BP	regulation of intracellular signal transduction	0.000246989	GO:1902531
	GO:BP	cellular developmental process	0.000246989	GO:0048869
	GO:BP	negative regulation of response to stimulus	0.000268665	GO:0048585
	GO:BP	regulation of multicellular organismal development	0.000268665	GO:2000026
	GO:BP	anatomical structure development	0.000268665	GO:0048856
	GO:BP	inactivation of MAPK activity	0.000268665	GO:0000188
	GO:BP	regulation of cell communication	0.000268665	GO:0010646
	GO:BP	animal organ morphogenesis	0.0002695	GO:0009887
	GO:BP	positive regulation of protein metabolic process	0.000306966	GO:0051247
	GO:BP	cell differentiation	0.000306966	GO:0030154
	GO:BP	embryo development	0.00031511	GO:0009790
	GO:BP	regulation of signaling	0.00033812	GO:0023051
	GO:BP	regulation of protein metabolic process	0.00035891	GO:0051246

	GO:MF	MAP kinase phosphatase activity	0.000359081	GO:0033549
	GO:BP	negative chemotaxis	0.00036024	GO:0050919
	GO:BP	positive regulation of molecular function	0.00036024	GO:0044093
	GO:BP	cell-substrate adhesion	0.000369048	GO:0031589
	GO:BP	positive regulation of cellular process	0.000447366	GO:0048522
	GO:BP	multicellular organism development	0.000447366	GO:0007275
	GO:CC	lamellipodium	0.000577259	GO:0030027
	GO:BP	positive regulation of biological process	0.000588812	GO:0048518
	GO:BP	signal transduction by p53 class mediator	0.000676253	GO:0072331
	GO:BP	positive regulation of developmental process	0.000676253	GO:0051094
	GO:BP	extracellular structure organization	0.000676253	GO:0043062
	GO:BP	nephron development	0.000676253	GO:0072006
	GO:BP	extracellular matrix organization	0.000676253	GO:0030198
	GO:BP	cell morphogenesis	0.000688925	GO:0000902
	GO:BP	mesodermal cell differentiation	0.000809959	GO:0048333
	GO:BP	cellular component morphogenesis	0.000835326	GO:0032989
	GO:BP	animal organ development	0.000835326	GO:0048513
	GO:BP	positive regulation of multicellular organismal process	0.000923163	GO:0051240
	GO:BP	synapse organization	0.001034767	GO:0050808
	GO:BP	axon guidance	0.00110664	GO:0007411
	GO:BP	regulation of nervous system development	0.001127625	GO:0051960
	GO:BP	camera-type eye development	0.001127625	GO:0043010
	GO:BP	cellular component organization	0.001127625	GO:0016043
	GO:BP	regulation of signal transduction	0.001127625	GO:0009966
	GO:BP	intrinsic apoptotic signaling pathway by p53 class mediator	0.001127625	GO:0072332
	GO:BP	regulation of cell differentiation	0.001127625	GO:0045595
	GO:BP	neuron projection guidance	0.001127625	GO:0097485
	GO:BP	lens development in camera-type eye	0.001127625	GO:0002088
	GO:BP	neural crest cell development	0.001127625	GO:0014032
	GO:BP	developmental process	0.001143813	GO:0032502
	GO:BP	sensory organ development	0.001143813	GO:0007423
	GO:BP	generation of neurons	0.001237296	GO:0048699
	GO:BP	eye development	0.001237296	GO:0001654
	GO:BP	positive regulation of macromolecule metabolic process	0.001237296	GO:0010604
	GO:BP	endoderm development	0.001237296	GO:0007492
	GO:BP	cellular component organization or biogenesis	0.001342805	GO:0071840
	GO:BP	renal system development	0.001342805	GO:0072001
	GO:BP	visual system development	0.001342805	GO:0150063
	GO:BP	stem cell development	0.001342805	GO:0048864
	GO:BP	mesenchymal cell development	0.001342805	GO:0014031
	GO:BP	DNA damage response, signal transduction by p53 class mediator	0.001342805	GO:0030330
	GO:BP	odontogenesis	0.001342805	GO:0042476

	GO:BP	urogenital system development	0.001379132	GO:0001655
	GO:BP	cell-matrix adhesion	0.001432088	GO:0007160
	GO:BP	sensory system development	0.001452271	GO:0048880
	GO:CC	cytoplasm	0.001458499	GO:0005737
	GO:BP	regulation of protein modification process	0.001468084	GO:0031399
	GO:BP	morphogenesis of a branching structure	0.001524106	GO:0001763
	GO:BP	nephron epithelium development	0.001524106	GO:0072009
	GO:BP	negative regulation of signal transduction	0.001524106	GO:0009968
	GO:BP	response to organic substance	0.001524106	GO:0010033
	GO:BP	tube development	0.00157922	GO:0035295
	GO:BP	negative regulation of cell communication	0.001625312	GO:0010648
	GO:BP	regulation of response to stimulus	0.001674783	GO:0048583
	GO:BP	negative regulation of signaling	0.001689794	GO:0023057
	GO:BP	cell morphogenesis involved in differentiation	0.001754096	GO:0000904
	GO:BP	neural crest cell differentiation	0.001754096	GO:0014033
	GO:BP	positive regulation of regulatory T cell differentiation	0.001756579	GO:0045591
	GO:BP	cell communication	0.001772028	GO:0007154
	GO:BP	kidney epithelium development	0.001772028	GO:0072073
	GO:BP	cell-cell adhesion	0.001829698	GO:0098609
	GO:BP	intrinsic apoptotic signaling pathway in response to DNA damage by p53 class mediator	0.001845061	GO:0042771
	GO:CC	plasma membrane region	0.001849115	GO:0098590
	GO:BP	neurogenesis	0.001904196	GO:0022008
	GO:BP	cell projection organization	0.002039321	GO:0030030
	GO:BP	positive regulation of cellular metabolic process	0.002258015	GO:0031325
	GO:BP	regulation of cell migration	0.002387785	GO:0030334
	GO:BP	protein phosphorylation	0.002414716	GO:0006468
	GO:BP	nephron tubule development	0.002467337	GO:0072080
	GO:BP	positive regulation of nitrogen compound metabolic process	0.002467337	GO:0051173
	GO:BP	regulation of cellular component organization	0.002467337	GO:0051128
	GO:BP	kidney development	0.002545981	GO:0001822
	GO:BP	positive regulation of endopeptidase activity	0.002557145	GO:0010950
	GO:BP	positive regulation of metabolic process	0.002557145	GO:0009893
	GO:BP	kidney morphogenesis	0.002557145	GO:0060993
	GO:BP	morphogenesis of a branching epithelium	0.002639729	GO:0061138
	GO:BP	tube morphogenesis	0.002788673	GO:0035239
	GO:BP	regulation of catalytic activity	0.002788673	GO:0050790
	GO:BP	cell development	0.002788673	GO:0048468
	GO:BP	renal tubule development	0.002807325	GO:0061326
	GO:BP	regulation of anatomical structure morphogenesis	0.002827974	GO:0022603
	GO:BP	gland morphogenesis	0.003013165	GO:0022612
	GO:BP	positive regulation of intracellular signal transduction	0.003103801	GO:1902533

	GO:BP	gland development	0.003172101	GO:0048732
	GO:BP	signaling	0.003271767	GO:0023052
	GO:BP	regulation of cell adhesion	0.003447125	GO:0030155
	GO:BP	cellular response to chemical stimulus	0.003458605	GO:0070887
	GO:CC	cell leading edge	0.003533082	GO:0031252
	GO:CC	integrin complex	0.003533082	GO:0008305
	GO:CC	focal adhesion	0.003533082	GO:0005925
	GO:CC	cell-substrate junction	0.003533082	GO:0030055
	GO:BP	cellular response to external stimulus	0.003639237	GO:0071496
	GO:BP	skin development	0.003845341	GO:0043588
	GO:BP	growth	0.003945238	GO:0040007
	GO:BP	positive regulation of cell adhesion	0.003945238	GO:0045785
	GO:BP	mesoderm formation	0.003945238	GO:0001707
	GO:BP	sensory organ morphogenesis	0.003945238	GO:0090596
	GO:BP	regulation of MAP kinase activity	0.003945238	GO:0043405
	GO:BP	endoderm formation	0.003945238	GO:0001706
	GO:BP	cell surface receptor signaling pathway	0.003945238	GO:0007166
	GO:BP	peptidyl-tyrosine dephosphorylation	0.003987744	GO:0035335
	GO:BP	positive regulation of cell differentiation	0.003996443	GO:0045597
	GO:BP	response to endogenous stimulus	0.00404587	GO:0009719
	GO:BP	protein metabolic process	0.00404587	GO:0019538
	GO:BP	signal transduction in response to DNA damage	0.004084204	GO:0042770
	GO:BP	muscle cell migration	0.004084204	GO:0014812
	GO:BP	localization	0.004084204	GO:0051179
	GO:BP	response to nutrient levels	0.004098862	GO:0031667
	GO:BP	regulation of DNA damage response, signal transduction by p53 class mediator	0.004101738	GO:0043516
	GO:BP	response to chemical	0.004101738	GO:0042221
	GO:BP	morphogenesis of an epithelium	0.00410566	GO:0002009
	GO:BP	mesoderm morphogenesis	0.00410566	GO:0048332
	GO:BP	regulation of cellular component movement	0.00410566	GO:0051270
	GO:BP	cellular protein metabolic process	0.004113559	GO:0044267
	GO:BP	cellular response to nutrient levels	0.004212392	GO:0031669
	GO:BP	chemotaxis	0.004311865	GO:0006935
	GO:BP	signal transduction	0.004311865	GO:0007165
	GO:BP	response to growth factor	0.004378519	GO:0070848
	GO:BP	response to external stimulus	0.004378519	GO:0009605
	GO:BP	taxis	0.004378519	GO:0042330
	GO:BP	mesoderm development	0.004378519	GO:0007498
	GO:BP	developmental growth	0.004403917	GO:0048589
	GO:BP	cellular protein modification process	0.004403917	GO:0006464
	GO:BP	protein modification process	0.004403917	GO:0036211

	GO:BP	positive regulation of peptidase activity	0.004406771	GO:0010952
	GO:BP	negative regulation of phosphorylation	0.004406771	GO:0042326
	GO:BP	regulation of morphogenesis of a branching structure	0.004406771	GO:0060688
	GO:BP	negative regulation of protein phosphorylation	0.004641324	GO:0001933
	GO:BP	regulation of cell motility	0.004641324	GO:2000145
	GO:BP	neuron differentiation	0.004935012	GO:0030182
	GO:BP	regulation of localization	0.004935012	GO:0032879
	GO:BP	cellular response to stimulus	0.00496081	GO:0051716
	GO:BP	cellular response to fatty acid	0.005088012	GO:0071398
	GO:BP	positive regulation of catalytic activity	0.005099597	GO:0043085
	GO:BP	regulation of neurogenesis	0.005498566	GO:0050767
	GO:BP	orthogonal dichotomous subdivision of terminal units involved in lung branching morphogenesis	0.005508529	GO:0060488
	GO:BP	lateral sprouting involved in lung morphogenesis	0.005508529	GO:0060490
	GO:BP	planar dichotomous subdivision of terminal units involved in lung branching morphogenesis	0.005508529	GO:0060489
	GO:CC	intracellular	0.005564465	GO:0005622
	GO:CC	protein complex involved in cell adhesion	0.005564465	GO:0098636
	GO:BP	regulation of cell population proliferation	0.005715829	GO:0042127
	GO:BP	regulation of phosphate metabolic process	0.005749258	GO:0019220
	GO:BP	nervous system development	0.005750228	GO:0007399
	GO:BP	regulation of phosphorus metabolic process	0.005750228	GO:0051174
	GO:BP	mammary gland development	0.005852429	GO:0030879
	GO:BP	regulation of locomotion	0.00588032	GO:0040012
	GO:BP	cell-cell signaling	0.005882962	GO:0007267
	GO:BP	plasma membrane bounded cell projection organization	0.006146066	GO:0120036
	GO:MF	insulin-like growth factor binding	0.006158957	GO:0005520
	GO:MF	protein binding	0.006158957	GO:0005515
	GO:MF	protein tyrosine/serine/threonine phosphatase activity	0.006158957	GO:0008138
	GO:BP	proteoglycan biosynthetic process	0.006732436	GO:0030166
	GO:BP	hair follicle development	0.006762609	GO:0001942
	GO:BP	regulation of protein phosphorylation	0.006762609	GO:0001932
	GO:BP	proteoglycan metabolic process	0.006762609	GO:0006029
	GO:BP	response to extracellular stimulus	0.00689885	GO:0009991
	GO:BP	plasma membrane bounded cell projection morphogenesis	0.007421939	GO:0120039
	GO:BP	negative regulation of nitrogen compound metabolic process	0.007421939	GO:0051172
	GO:BP	hair cycle process	0.007421939	GO:0022405
	GO:BP	macromolecule modification	0.007421939	GO:0043412
	GO:BP	skin epidermis development	0.007421939	GO:0098773
	GO:BP	molting cycle process	0.007421939	GO:0022404
	GO:BP	response to fatty acid	0.007421939	GO:0070542
	GO:BP	desmosome organization	0.007609626	GO:0002934

	GO:BP	smooth muscle cell migration	0.007853358	GO:0014909
	GO:BP	cell projection morphogenesis	0.007894658	GO:0048858
	GO:BP	camera-type eye morphogenesis	0.008129456	GO:0048593
	GO:CC	synapse	0.00851409	GO:0045202
	GO:CC	intrinsic component of plasma membrane	0.00851409	GO:0031226
	GO:CC	basolateral plasma membrane	0.00851409	GO:0016323
	GO:BP	regulation of cell development	0.008644407	GO:0060284
	GO:BP	response to stimulus	0.008644989	GO:0050896
	GO:BP	regulation of synapse organization	0.009566781	GO:0050807
	GO:BP	positive regulation of protein modification process	0.009566781	GO:0031401
	GO:BP	epidermis development	0.009575339	GO:0008544
	GO:BP	cellular response to extracellular stimulus	0.009575339	GO:0031668
	GO:BP	skin morphogenesis	0.009826302	GO:0043589
	GO:BP	cell population proliferation	0.010161266	GO:0008283
	GO:BP	cellular response to endogenous stimulus	0.010249488	GO:0071495
	GO:MF	protein tyrosine phosphatase activity	0.010688876	GO:0004725
	GO:MF	cell adhesion molecule binding	0.010688876	GO:0050839
	GO:CC	integral component of plasma membrane	0.010881537	GO:0005887
	GO:CC	intracellular organelle	0.010881537	GO:0043229
	GO:CC	cell-cell junction	0.010881537	GO:0005911
	GO:BP	cell part morphogenesis	0.010981342	GO:0032990
	GO:BP	cellular response to organic substance	0.011236528	GO:0071310
	GO:BP	organonitrogen compound metabolic process	0.011315349	GO:1901564
	GO:BP	mammary gland morphogenesis	0.011458487	GO:0060443
	GO:BP	response to acid chemical	0.011717747	GO:0001101
	GO:BP	neuron projection development	0.012064579	GO:0031175
	GO:BP	regulation of phosphorylation	0.012064579	GO:0042325
	GO:BP	regionalization	0.012206893	GO:0003002
	GO:BP	axon development	0.012215775	GO:0061564
	GO:BP	axonogenesis	0.012462352	GO:0007409
	GO:BP	mammary gland epithelium development	0.01249355	GO:0061180
	GO:BP	neuron projection morphogenesis	0.012555446	GO:0048812
	GO:BP	dichotomous subdivision of terminal units involved in lung branching	0.012802554	GO:0060448
	GO:BP	regulation of synapse structure or activity	0.012926069	GO:0050803
	GO:BP	aortic valve morphogenesis	0.01300305	GO:0003180
	GO:BP	nephron tubule morphogenesis	0.013124158	GO:0072078
	GO:BP	phosphorylation	0.013235083	GO:0016310
	GO:BP	inner ear development	0.013268978	GO:0048839
	GO:BP	developmental growth involved in morphogenesis	0.01351172	GO:0060560
	GO:BP	mesenchyme development	0.01351172	GO:0060485
	GO:BP	positive regulation of signal transduction	0.01405536	GO:0009967
	GO:BP	substrate-dependent cell migration	0.014443451	GO:0006929

	GO:BP	negative regulation of ERK1 and ERK2 cascade	0.014469026	GO:0070373
	GO:BP	lateral sprouting from an epithelium	0.014469026	GO:0060601
	GO:BP	nephron epithelium morphogenesis	0.014469026	GO:0072088
	GO:BP	white fat cell differentiation	0.014469026	GO:0050872
	GO:BP	regulation of neuron differentiation	0.014469026	GO:0045664
	GO:BP	intrinsic apoptotic signaling pathway in response to DNA damage	0.014469026	GO:0008630
	GO:BP	cranial nerve development	0.014852713	GO:0021545
	GO:BP	positive regulation of proteolysis	0.014852713	GO:0045862
	GO:CC	laminin-5 complex	0.015496016	GO:0005610
	GO:MF	transcription corepressor activity	0.015592739	GO:0003714
	GO:MF	growth factor binding	0.015592739	GO:0019838
	GO:BP	cranial nerve morphogenesis	0.01576619	GO:0021602
	GO:BP	regulation of nitrogen compound metabolic process	0.01576619	GO:0051171
	GO:BP	renal tubule morphogenesis	0.016019656	GO:0061333
	GO:BP	nephron morphogenesis	0.016019656	GO:0072028
	GO:BP	multicellular organismal process	0.016484383	GO:0032501
	GO:BP	protein deacylation	0.016521529	GO:0035601
	GO:BP	negative regulation of protein metabolic process	0.016610686	GO:0051248
	GO:BP	negative regulation of phosphate metabolic process	0.01672936	GO:0045936
	GO:BP	negative regulation of intracellular signal transduction	0.01672936	GO:1902532
	GO:BP	response to organic cyclic compound	0.016876345	GO:0014070
	GO:BP	negative regulation of phosphorus metabolic process	0.016876345	GO:0010563
	GO:BP	cellular response to cAMP	0.016876345	GO:0071320
	GO:BP	integrin-mediated signaling pathway	0.016940696	GO:0007229
	GO:BP	positive regulation of cellular component movement	0.017377691	GO:0051272
	GO:BP	negative regulation of MAP kinase activity	0.017481227	GO:0043407
	GO:BP	macromolecule deacylation	0.017659191	GO:0098732
	GO:BP	neural crest cell migration	0.017982974	GO:0001755
	GO:BP	positive regulation of cell death	0.018267513	GO:0010942
	GO:BP	positive regulation of cell migration	0.018624538	GO:0030335
	GO:BP	regulation of cell-substrate adhesion	0.018624538	GO:0010810
	GO:BP	heparan sulfate proteoglycan metabolic process	0.018624538	GO:0030201
	GO:BP	aortic valve development	0.018624538	GO:0003176
	GO:BP	intrinsic apoptotic signaling pathway	0.018961582	GO:0097193
	GO:BP	epithelial cell development	0.018961582	GO:0002064
	GO:BP	regulation of MAPK cascade	0.019270698	GO:0043408
	GO:BP	negative regulation of cellular metabolic process	0.019270698	GO:0031324
	GO:BP	cell junction organization	0.01927526	GO:0034330
	GO:BP	regulation of signal transduction by p53 class mediator	0.019387307	GO:1901796
	GO:BP	regulation of animal organ morphogenesis	0.019387307	GO:2000027
	GO:BP	cell morphogenesis involved in neuron differentiation	0.019394273	GO:0048667
	GO:BP	hair cycle	0.019411319	GO:0042633

	GO:BP	molting cycle	0.019411319	GO:0042303
	GO:BP	mesenchymal cell differentiation	0.019547629	GO:0048762
	GO:BP	ameboidal-type cell migration	0.019547629	GO:0001667
	GO:BP	cell surface receptor signaling pathway involved in heart development	0.019768184	GO:0061311
	GO:BP	regulation of protein serine/threonine kinase activity	0.019768184	GO:0071900
	GO:BP	regulation of regulatory T cell differentiation	0.019768184	GO:0045589
	GO:BP	renal sodium ion absorption	0.019897553	GO:0070294
	GO:BP	establishment of body hair planar orientation	0.019897553	GO:0048105
	GO:BP	frontal suture morphogenesis	0.019897553	GO:0060364
	GO:BP	establishment of body hair or bristle planar orientation	0.019897553	GO:0048104
	GO:BP	positive regulation of phosphorus metabolic process	0.020001252	GO:0010562
	GO:BP	positive regulation of phosphate metabolic process	0.020001252	GO:0045937
	GO:BP	negative regulation of developmental process	0.020333567	GO:0051093
	GO:BP	cellular response to growth factor stimulus	0.020743676	GO:0071363
	GO:BP	regulation of endopeptidase activity	0.021353027	GO:0052548
	GO:BP	liver regeneration	0.021353027	GO:0097421
	GO:BP	regulation of hydrolase activity	0.022311377	GO:0051336
	GO:BP	positive regulation of DNA damage response, signal transduction by p53 class mediator	0.022311377	GO:0043517
	GO:BP	peptidyl-threonine dephosphorylation	0.022311377	GO:0035970
	GO:BP	renal absorption	0.022311377	GO:0070293
	GO:BP	negative regulation of MAPK cascade	0.022343131	GO:0043409
	GO:BP	negative regulation of cellular protein metabolic process	0.022497694	GO:0032269
	GO:BP	branching morphogenesis of an epithelial tube	0.023076063	GO:0048754
	GO:BP	phosphate-containing compound metabolic process	0.023076063	GO:0006796
	GO:BP	regulation of JUN kinase activity	0.023168174	GO:0043506
	GO:BP	response to nutrient	0.023432809	GO:0007584
	GO:BP	fat cell differentiation	0.023432809	GO:0045444
	GO:BP	eye morphogenesis	0.023704447	GO:0048592
	GO:BP	positive regulation of cysteine-type endopeptidase activity	0.024535078	GO:2001056
	GO:BP	morphogenesis of embryonic epithelium	0.024535078	GO:0016331
	GO:BP	regulation of cell death	0.024911814	GO:0010941
	GO:BP	positive regulation of cell motility	0.024938381	GO:2000147
	GO:BP	ear development	0.024938381	GO:0043583
	GO:BP	neuron development	0.024938381	GO:0048666
	GO:BP	regulation of primary metabolic process	0.024938381	GO:0080090
	GO:BP	cell death	0.024938381	GO:0008219
	GO:BP	regulatory T cell differentiation	0.024938381	GO:0045066
	GO:BP	preganglionic parasympathetic fiber development	0.024938381	GO:0021783
	GO:BP	DNA damage response, signal transduction by p53 class mediator resulting in transcription of p21 class mediator	0.024938381	GO:0006978
	GO:BP	regulation of mammary gland epithelial cell proliferation	0.024938381	GO:0033599

	GO:MF	kinase binding	0.025041354	GO:0019900
	GO:MF	enzyme binding	0.025341075	GO:0019899
	GO:BP	negative regulation of protein modification process	0.02597985	GO:0031400
	GO:BP	negative regulation of cellular process	0.026036753	GO:0048523
	GO:CC	insulin-like growth factor ternary complex	0.026325337	GO:0042567
	GO:CC	alphav-beta3 integrin-IGF-1-IGF1R complex	0.026325337	GO:0035867
	GO:CC	integral component of presynaptic active zone membrane	0.026325337	GO:0099059
	GO:BP	MAPK cascade	0.026328557	GO:0000165
	GO:BP	epithelial cell differentiation	0.026348063	GO:0030855
	GO:BP	phosphorus metabolic process	0.026348063	GO:0006793
	GO:BP	semi-lunar valve development	0.026355989	GO:1905314
	GO:BP	positive regulation of hemopoiesis	0.026355989	GO:1903708
	GO:BP	response to vitamin D	0.026355989	GO:0033280
	GO:BP	regulation of cell-matrix adhesion	0.026775326	GO:0001952
	GO:BP	glycosaminoglycan metabolic process	0.026940717	GO:0030203
	GO:BP	pattern specification process	0.026940717	GO:0007389
	GO:BP	ossification	0.027353027	GO:0001503
	GO:BP	microglia differentiation	0.027353027	GO:0014004
	GO:BP	positive regulation of transferase activity	0.027353027	GO:0051347
	GO:BP	semaphorin-plexin signaling pathway	0.027353027	GO:0071526
	GO:BP	apoptotic process	0.027353027	GO:0006915
	GO:BP	trigeminal ganglion development	0.027353027	GO:0061551
	GO:BP	regulation of branching involved in mammary gland duct morphogenesis	0.027353027	GO:0060762
	GO:BP	epithelial cell proliferation	0.027353027	GO:0050673
	GO:BP	regulation of epithelial tube formation	0.027353027	GO:1905276
	GO:BP	cellular response to glucose starvation	0.027353027	GO:0042149
	GO:BP	substrate-dependent cerebral cortex tangential migration	0.027353027	GO:0021825
	GO:BP	positive regulation of phosphorylation	0.027353027	GO:0042327
	GO:BP	cell migration involved in kidney development	0.027353027	GO:0035787
	GO:BP	odontogenesis of dentin-containing tooth	0.027353027	GO:0042475
	GO:BP	DNA damage response, signal transduction resulting in transcription	0.027353027	GO:0042772
	GO:BP	regulation of apoptotic process	0.027353027	GO:0042981
	GO:BP	positive regulation of apoptotic process	0.027353027	GO:0043065
	GO:BP	negative regulation of response to wounding	0.028240025	GO:1903035
	GO:CC	apical part of cell	0.028593876	GO:0045177
	GO:BP	platelet degranulation	0.02876038	GO:0002576
	GO:BP	positive regulation of cell communication	0.02897467	GO:0010647
	GO:BP	regulation of cellular protein localization	0.029202612	GO:1903827
	GO:BP	insulin-like growth factor receptor signaling pathway	0.029692355	GO:0048009
	GO:BP	positive regulation of programmed cell death	0.030057413	GO:0043068
	GO:MF	fibronectin binding	0.030135768	GO:0001968
	GO:CC	insulin-like growth factor binding protein complex	0.030436575	GO:0016942

	GO:CC	insulin receptor complex	0.030436575	GO:0005899
	GO:CC	filopodium membrane	0.030436575	GO:0031527
	GO:CC	growth factor complex	0.030436575	GO:0036454
	GO:CC	membrane-enclosed lumen	0.030436575	GO:0031974
	GO:CC	collagen-containing extracellular matrix	0.030436575	GO:0062023
	GO:CC	intracellular organelle lumen	0.030436575	GO:0070013
	GO:CC	organelle lumen	0.030436575	GO:0043233
	GO:BP	regulation of protein kinase activity	0.03058132	GO:0045859
	GO:BP	positive regulation of signaling	0.03058132	GO:0023056
	GO:BP	signal transduction by protein phosphorylation	0.03058132	GO:0023014
	GO:BP	regulation of DNA binding	0.030610563	GO:0051101
	GO:BP	response to vitamin	0.030610563	GO:0033273
	GO:BP	negative regulation of protein kinase activity	0.030612678	GO:0006469
	GO:BP	parasympathetic nervous system development	0.030612678	GO:0048486
	GO:BP	response to wounding	0.030837461	GO:0009611
	GO:BP	regulation of peptidase activity	0.031454655	GO:0052547
	GO:BP	regulation of cell projection organization	0.031493085	GO:0031344
	GO:BP	ureteric bud morphogenesis	0.031493085	GO:0060675
	GO:BP	cranial skeletal system development	0.031493085	GO:1904888
	GO:BP	response to ketone	0.031611128	GO:1901654
	GO:BP	respiratory system development	0.031611128	GO:0060541
	GO:BP	regulation of transferase activity	0.03186139	GO:0051338
	GO:BP	regulation of kinase activity	0.03186139	GO:0043549
	GO:BP	programmed cell death	0.032065744	GO:0012501
	GO:CC	intrinsic component of presynaptic active zone membrane	0.032688029	GO:0098945
	GO:CC	apical plasma membrane	0.032688029	GO:0016324
	GO:CC	membrane-bounded organelle	0.032874203	GO:0043227
	GO:BP	mesonephric tubule morphogenesis	0.032971937	GO:0072171
	GO:BP	protein dephosphorylation	0.032971937	GO:0006470
	GO:BP	regulation of cysteine-type endopeptidase activity	0.033218015	GO:2000116
	GO:BP	endothelium development	0.033307053	GO:0003158
	GO:CC	basement membrane	0.033367357	GO:0005604
	GO:BP	positive regulation of cellular protein localization	0.033402583	GO:1903829
	GO:BP	enzyme linked receptor protein signaling pathway	0.033402583	GO:0007167
	GO:BP	regulation of programmed cell death	0.033402583	GO:0043067
	GO:CC	organelle	0.033413348	GO:0043226
	GO:BP	protein deacetylation	0.033715243	GO:0006476
	GO:BP	regulation of ossification	0.033719072	GO:0030278
	GO:BP	cell-substrate junction organization	0.034701111	GO:0150115
	GO:BP	regulation of cellular response to stress	0.034701111	GO:0080135
	GO:BP	positive regulation of kinase activity	0.034701111	GO:0033674
	GO:BP	ureteric bud development	0.034701111	GO:0001657

	GO:BP	positive regulation of locomotion	0.034701111	GO:0040017
	GO:BP	substrate adhesion-dependent cell spreading	0.034701111	GO:0034446
	GO:BP	cell-substrate junction assembly	0.034701111	GO:0007044
	GO:BP	cranial ganglion development	0.035130889	GO:0061550
	GO:BP	regulation of microvillus assembly	0.035130889	GO:0032534
	GO:BP	extracellular matrix assembly	0.035130889	GO:0085029
	GO:BP	regulation of heart morphogenesis	0.035130889	GO:2000826
	GO:BP	aminoglycan metabolic process	0.035130889	GO:0006022
	GO:BP	negative regulation of cell differentiation	0.035130889	GO:0045596
	GO:BP	renal sodium ion transport	0.035130889	GO:0003096
	GO:BP	positive regulation of receptor clustering	0.035130889	GO:1903911
	GO:BP	response to dexamethasone	0.035130889	GO:0071548
	GO:BP	regeneration	0.035290815	GO:0031099
	GO:BP	response to organonitrogen compound	0.035290815	GO:0010243
	GO:BP	mesonephric tubule development	0.035290815	GO:0072164
	GO:BP	mesonephric epithelium development	0.035290815	GO:0072163
	GO:BP	positive regulation of protein phosphorylation	0.035646473	GO:0001934
	GO:BP	myeloid cell differentiation	0.035680871	GO:0030099
	GO:BP	negative regulation of transcription by RNA polymerase II	0.035680871	GO:0000122
	GO:CC	intracellular membrane-bounded organelle	0.036679203	GO:0043231
	GO:BP	myeloid cell development	0.036706295	GO:0061515
	GO:BP	positive regulation of protein acetylation	0.036865096	GO:1901985
	GO:BP	postsynapse organization	0.037907298	GO:0099173
	GO:BP	regulation of macromolecule metabolic process	0.037907298	GO:0060255
	GO:BP	artery development	0.038089842	GO:0060840
	GO:BP	cell adhesion mediated by integrin	0.038539152	GO:0033627
	GO:BP	cellular response to lipid	0.038903248	GO:0071396
	GO:BP	autonomic nervous system development	0.0394929	GO:0048483
	GO:BP	Wnt signaling pathway	0.039501241	GO:0016055
	GO:BP	negative regulation of growth	0.039723357	GO:0045926
	GO:BP	JNK cascade	0.039723357	GO:0007254
	GO:BP	positive regulation of JUN kinase activity	0.040325264	GO:0043507
	GO:BP	retina layer formation	0.040488532	GO:0010842
	GO:BP	cell-cell signaling by wnt	0.040488532	GO:0198738
	GO:BP	cellular response to acid chemical	0.040508403	GO:0071229
	GO:BP	mesonephros development	0.04057616	GO:0001823
	GO:BP	inner ear morphogenesis	0.04057616	GO:0042472
	GO:BP	negative regulation of macromolecule metabolic process	0.04057616	GO:0010605
	GO:BP	response to organophosphorus	0.04090829	GO:0046683
	GO:BP	endodermal cell differentiation	0.041388298	GO:0035987
	GO:BP	regulation of cellular metabolic process	0.041388298	GO:0031323
	GO:BP	establishment of endothelial barrier	0.041388298	GO:0061028

	GO:BP	lung development	0.041515148	GO:0030324
	GO:BP	blood vessel development	0.041828959	GO:0001568
	GO:CC	filopodium	0.043333421	GO:0030175
	GO:MF	integrin binding	0.043597122	GO:0005178
	GO:BP	wound healing	0.043779697	GO:0042060
	GO:BP	positive regulation of signal transduction by p53 class mediator	0.044292733	GO:1901798
	GO:BP	urea transmembrane transport	0.044292733	GO:0071918
	GO:BP	response to stress	0.044292733	GO:0006950
	GO:BP	branching involved in salivary gland morphogenesis	0.044292733	GO:0060445
	GO:BP	lens morphogenesis in camera-type eye	0.044292733	GO:0002089
	GO:BP	regulation of synapse assembly	0.045208554	GO:0051963
	GO:BP	negative regulation of kinase activity	0.045418326	GO:0033673
	GO:BP	response to nitrogen compound	0.045418326	GO:1901698
	GO:BP	positive regulation of MAP kinase activity	0.045418326	GO:0043406
	GO:BP	cellular response to nutrient	0.045431102	GO:0031670
	GO:BP	respiratory tube development	0.04602601	GO:0030323
	GO:BP	regulation of protein deacetylation	0.046628103	GO:0090311
	GO:BP	glycosaminoglycan biosynthetic process	0.046628103	GO:0006024
	GO:BP	transmembrane receptor protein tyrosine kinase signaling pathway	0.048003921	GO:0007169
	GO:BP	regulation of proteolysis	0.048577673	GO:0030162
	GO:BP	regulation of morphogenesis of an epithelium	0.048577673	GO:1905330
	GO:BP	osteoblast differentiation	0.049576537	GO:0001649
	GO:BP	regulation of JNK cascade	0.049831639	GO:0046328
	GO:BP	animal organ regeneration	0.049831639	GO:0031100
	KEGG	PI3K-Akt signaling pathway	6.71621E-07	04151
	KEGG	Proteoglycans in cancer	4.40632E-05	05205
	KEGG	Human papillomavirus infection	4.40632E-05	05165
	KEGG	ECM-receptor interaction	5.22374E-05	04512
	KEGG	FoxO signaling pathway	5.22374E-05	04068
	KEGG	MAPK signaling pathway	5.22374E-05	04010
	KEGG	MicroRNAs in cancer	7.11452E-05	05206
	KEGG	Pathways in cancer	7.27234E-05	05200
	KEGG	Focal adhesion	7.27234E-05	04510
	KEGG	Axon guidance	0.000276399	04360
	KEGG	Transcriptional misregulation in cancer	0.000303578	05202
	KEGG	Hypertrophic cardiomyopathy (HCM)	0.000326172	05410
	KEGG	Regulation of actin cytoskeleton	0.004282057	04810
	KEGG	Dilated cardiomyopathy (DCM)	0.004282057	05414
	KEGG	Hippo signaling pathway	0.00443507	04390
	KEGG	AMPK signaling pathway	0.009952162	04152
	KEGG	Arrhythmogenic right ventricular cardiomyopathy (ARVC)	0.014087021	05412
	KEGG	Apoptosis	0.014087021	04210

	KEGG	Autophagy - animal	0.014087021	04140
	KEGG	Apelin signaling pathway	0.014087021	04371
	KEGG	Fluid shear stress and atherosclerosis	0.014273056	05418
	KEGG	Rap1 signaling pathway	0.014494993	04015
	KEGG	mTOR signaling pathway	0.019579919	04150
	KEGG	Small cell lung cancer	0.019603048	05222
	KEGG	Wnt signaling pathway	0.021739003	04310
	KEGG	Prostate cancer	0.021760444	05215
	KEGG	Tight junction	0.025290339	04530
	KEGG	Toxoplasmosis	0.033195282	05145
**K20-only vs double-high**				
**Upregulated in the K20-only group**	**Source**	**Terms**	**FDR**	**Term ID**
	GO:CC	intracellular membrane-bounded organelle	4.1289E-08	GO:0043231
	GO:CC	membrane-bounded organelle	1.75618E-06	GO:0043227
	GO:CC	chromosome	3.14643E-06	GO:0005694
	GO:CC	organelle	7.13081E-06	GO:0043226
	GO:CC	cytoplasm	8.30967E-06	GO:0005737
	GO:CC	intracellular organelle	8.44585E-06	GO:0043229
	GO:CC	non-membrane-bounded organelle	1.20368E-05	GO:0043228
	GO:CC	intracellular	1.44797E-05	GO:0005622
	GO:CC	organelle lumen	1.44797E-05	GO:0043233
	GO:CC	intracellular non-membrane-bounded organelle	1.44797E-05	GO:0043232
	GO:CC	intracellular organelle lumen	1.44797E-05	GO:0070013
	GO:CC	membrane-enclosed lumen	1.44797E-05	GO:0031974
	GO:CC	nucleoplasm	4.83269E-05	GO:0005654
	GO:BP	organelle organization	6.49236E-05	GO:0006996
	GO:CC	nuclear lumen	0.000121879	GO:0031981
	GO:BP	cell cycle	0.000149184	GO:0007049
	GO:BP	chromosome organization	0.000149184	GO:0051276
	GO:BP	mitotic cell cycle	0.000149184	GO:0000278
	GO:CC	nucleus	0.000200963	GO:0005634
	GO:MF	nucleotide binding	0.000485574	GO:0000166
	GO:MF	nucleoside phosphate binding	0.000485574	GO:1901265
	GO:BP	cellular component organization or biogenesis	0.000555988	GO:0071840
	GO:BP	cellular component organization	0.000555988	GO:0016043
	GO:CC	DNA packaging complex	0.00061003	GO:0044815
	GO:CC	nuclear lamina	0.00061003	GO:0005652
	GO:CC	microtubule cytoskeleton	0.00061003	GO:0015630
	GO:BP	DNA packaging	0.000637039	GO:0006323
	GO:BP	cell division	0.000716599	GO:0051301
	GO:BP	mitotic cell cycle process	0.000718505	GO:1903047
	GO:BP	DNA replication	0.000743307	GO:0006260

	GO:BP	DNA metabolic process	0.000743307	GO:0006259
	GO:BP	nucleosome assembly	0.000818422	GO:0006334
	GO:BP	nuclear DNA replication	0.001124001	GO:0033260
	GO:BP	DNA conformation change	0.001124001	GO:0071103
	GO:CC	chromosomal region	0.001145384	GO:0098687
	GO:CC	condensed chromosome	0.001145384	GO:0000793
	GO:CC	nucleosome	0.001814486	GO:0000786
	GO:BP	cell cycle DNA replication	0.001957256	GO:0044786
	GO:BP	cell cycle process	0.002050262	GO:0022402
	GO:BP	G1/S transition of mitotic cell cycle	0.002057291	GO:0000082
	GO:BP	chromatin assembly	0.002180458	GO:0031497
	GO:BP	sister chromatid segregation	0.002180458	GO:0000819
	GO:CC	chromatin	0.002503053	GO:0000785
	GO:CC	protein-DNA complex	0.002644973	GO:0032993
	GO:CC	microtubule	0.003023902	GO:0005874
	GO:CC	cytosol	0.003703648	GO:0005829
	GO:CC	spindle	0.004007485	GO:0005819
	GO:BP	cell cycle G1/S phase transition	0.004705247	GO:0044843
	GO:MF	small molecule binding	0.00595397	GO:0036094
	GO:BP	regulation of cellular response to heat	0.006731341	GO:1900034
	GO:BP	nucleosome organization	0.006739739	GO:0034728
	GO:CC	endomembrane system	0.007191631	GO:0012505
	GO:BP	cellular response to heat	0.007254042	GO:0034605
	GO:BP	chromatin assembly or disassembly	0.00762799	GO:0006333
	GO:CC	cytoskeleton	0.00853424	GO:0005856
	GO:BP	inner cell mass cell proliferation	0.009064151	GO:0001833
	GO:CC	apical part of cell	0.010534337	GO:0045177
	GO:BP	mitotic cell cycle phase transition	0.011873487	GO:0044772
	GO:CC	clathrin-coated pit	0.012403801	GO:0005905
	GO:BP	response to heat	0.013181646	GO:0009408
	GO:BP	nuclear chromosome segregation	0.013181646	GO:0098813
	GO:BP	mitotic nuclear division	0.014903912	GO:0140014
	GO:BP	cellular response to DNA damage stimulus	0.015714112	GO:0006974
	GO:BP	nuclear division	0.015714112	GO:0000280
	GO:CC	cytoplasmic microtubule	0.016277144	GO:0005881
	GO:BP	DNA-dependent DNA replication	0.018285705	GO:0006261
	GO:BP	regulation of gene silencing	0.018285705	GO:0060968
	GO:BP	chromatin organization involved in regulation of transcription	0.018285705	GO:0034401
	GO:BP	regulation of mitotic cell cycle	0.018498469	GO:0007346
	GO:MF	anion binding	0.018707973	GO:0043168
	GO:MF	purine ribonucleotide binding	0.018707973	GO:0032555
	GO:MF	ribonucleotide binding	0.018707973	GO:0032553

	GO:MF	purine nucleotide binding	0.018707973	GO:0017076
	GO:MF	protein binding	0.018707973	GO:0005515
	GO:CC	spindle pole	0.019744383	GO:0000922
	GO:CC	chromosome, centromeric region	0.019744383	GO:0000775
	GO:CC	endolysosome membrane	0.019744383	GO:0036020
	GO:CC	envelope	0.019744383	GO:0031975
	GO:CC	organelle envelope	0.019744383	GO:0031967
	GO:CC	nuclear envelope	0.020040992	GO:0005635
	GO:CC	condensed chromosome kinetochore	0.021132865	GO:0000777
	GO:MF	leukotriene B4 receptor activity	0.02226105	GO:0001632
	GO:MF	adenyl ribonucleotide binding	0.02226105	GO:0032559
	GO:BP	mitotic sister chromatid segregation	0.022516454	GO:0000070
	GO:MF	adenyl nucleotide binding	0.022915177	GO:0030554
	GO:BP	cell cycle phase transition	0.023559223	GO:0044770
	GO:BP	DNA recombination	0.023559223	GO:0006310
	GO:BP	chromosome segregation	0.023559223	GO:0007059
	GO:BP	protein-DNA complex assembly	0.023559223	GO:0065004
	GO:CC	nuclear chromosome	0.024661921	GO:0000228
	GO:BP	blastocyst growth	0.024842795	GO:0001832
	GO:CC	kinesin complex	0.02509537	GO:0005871
	GO:CC	extracellular exosome	0.027237753	GO:0070062
	GO:MF	carbohydrate derivative binding	0.028668158	GO:0097367
	GO:MF	clathrin adaptor activity	0.028668158	GO:0035615
	GO:MF	purine ribonucleoside triphosphate binding	0.028668158	GO:0035639
	GO:CC	euchromatin	0.030451239	GO:0000791
	GO:CC	extracellular vesicle	0.030451239	GO:1903561
	GO:CC	extracellular organelle	0.030451239	GO:0043230
	GO:CC	lamin filament	0.030451239	GO:0005638
	GO:CC	basal plasma membrane	0.030451239	GO:0009925
	GO:CC	transcription export complex 2	0.030451239	GO:0070390
	GO:CC	rDNA heterochromatin	0.030451239	GO:0033553
	GO:BP	cofactor metabolic process	0.030691006	GO:0051186
	GO:CC	nuclear membrane	0.031338261	GO:0031965
	GO:CC	organelle membrane	0.031786381	GO:0031090
	GO:CC	condensed chromosome, centromeric region	0.031786381	GO:0000779
	GO:BP	negative regulation of mitotic cell cycle	0.032174074	GO:0045930
	GO:BP	chromatin organization involved in negative regulation of transcription	0.034150984	GO:0097549
	GO:BP	chromatin silencing	0.034150984	GO:0006342
	GO:BP	chromatin silencing at rDNA	0.034150984	GO:0000183
	GO:BP	regulation of gene silencing by miRNA	0.034150984	GO:0060964
	GO:BP	organelle fission	0.034150984	GO:0048285
	GO:BP	response to UV	0.034150984	GO:0009411

	GO:BP	regulation of gene silencing by RNA	0.036540115	GO:0060966
	GO:BP	regulation of posttranscriptional gene silencing	0.036540115	GO:0060147
	GO:CC	platelet dense granule membrane	0.038107295	GO:0031088
	GO:CC	endolysosome	0.038107295	GO:0036019
	GO:BP	glial cell activation	0.040890465	GO:0061900
	GO:MF	cargo adaptor activity	0.044119364	GO:0140312
	GO:MF	ATP binding	0.044119364	GO:0005524
	GO:MF	ATPase activity, coupled	0.044119364	GO:0042623
	GO:MF	drug binding	0.044119364	GO:0008144
	GO:BP	DNA repair	0.047063146	GO:0006281
	GO:BP	regulation of DNA metabolic process	0.047063146	GO:0051052
	GO:CC	lysosomal membrane	0.049272792	GO:0005765
	GO:CC	lytic vacuole membrane	0.049272792	GO:0098852
	GO:CC	chromatin silencing complex	0.049978784	GO:0005677
	KEGG	Metabolic pathways	8.55067E-07	01100
	KEGG	Cell cycle	1.12757E-05	04110
	KEGG	Systemic lupus erythematosus	0.000150756	05322
	KEGG	Endocytosis	0.000192217	04144
	KEGG	Alcoholism	0.000877696	05034
	KEGG	Tight junction	0.002944408	04530
	KEGG	DNA replication	0.002944408	03030
	KEGG	Peroxisome	0.005047773	04146
	KEGG	Viral carcinogenesis	0.006164176	05203
	KEGG	Huntington disease	0.030965791	05016
	KEGG	EGFR tyrosine kinase inhibitor resistance	0.030965791	01521
	KEGG	ErbB signaling pathway	0.036701344	04012
**Upregulated in the double-high group**	**Source**	**Terms**	**FDR**	**Term ID**
	GO:CC	anchoring junction	2.11958E-08	GO:0070161
	GO:CC	cytosolic ribosome	2.11958E-08	GO:0022626
	GO:BP	cotranslational protein targeting to membrane	5.6695E-08	GO:0006613
	GO:BP	SRP-dependent cotranslational protein targeting to membrane	5.6695E-08	GO:0006614
	GO:BP	nuclear-transcribed mRNA catabolic process, nonsense-mediated decay	5.6695E-08	GO:0000184
	GO:BP	protein targeting to ER	1.20044E-07	GO:0045047
	GO:BP	nuclear-transcribed mRNA catabolic process	1.49146E-07	GO:0000956
	GO:BP	establishment of protein localization to endoplasmic reticulum	1.49146E-07	GO:0072599
	GO:CC	focal adhesion	4.23362E-07	GO:0005925
	GO:CC	cell-substrate junction	4.38106E-07	GO:0030055
	GO:CC	cell junction	9.77692E-07	GO:0030054
	GO:BP	protein localization to endoplasmic reticulum	1.57983E-06	GO:0070972
	GO:CC	ribosomal subunit	1.84462E-06	GO:0044391
	GO:CC	cytosolic large ribosomal subunit	3.47112E-06	GO:0022625
	GO:BP	protein targeting to membrane	1.52897E-05	GO:0006612

	GO:BP	translational initiation	1.52897E-05	GO:0006413
	GO:BP	viral transcription	2.65144E-05	GO:0019083
	GO:MF	structural constituent of ribosome	2.70758E-05	GO:0003735
	GO:CC	ribosome	3.34825E-05	GO:0005840
	GO:BP	viral gene expression	7.48562E-05	GO:0019080
	GO:CC	large ribosomal subunit	0.000130265	GO:0015934
	GO:CC	intracellular	0.000130265	GO:0005622
	GO:BP	cell junction organization	0.000382445	GO:0034330
	GO:BP	establishment of protein localization to membrane	0.000382445	GO:0090150
	GO:BP	mRNA catabolic process	0.000382445	GO:0006402
	GO:BP	establishment of protein localization to organelle	0.000382445	GO:0072594
	GO:BP	cell junction assembly	0.000792605	GO:0034329
	GO:CC	ribonucleoprotein complex	0.001017745	GO:1990904
	GO:CC	stress fiber	0.001017745	GO:0001725
	GO:CC	contractile actin filament bundle	0.001017745	GO:0097517
	GO:CC	cytosol	0.00114302	GO:0005829
	GO:CC	cell-cell junction	0.001444864	GO:0005911
	GO:CC	actin filament bundle	0.001444864	GO:0032432
	GO:BP	RNA catabolic process	0.001461661	GO:0006401
	GO:BP	cytoplasmic translation	0.001461661	GO:0002181
	GO:CC	polysomal ribosome	0.001514409	GO:0042788
	GO:BP	signal transduction by p53 class mediator	0.001670279	GO:0072331
	GO:BP	positive regulation of cell cycle arrest	0.001670279	GO:0071158
	GO:BP	DNA damage response, signal transduction by p53 class mediator	0.001670279	GO:0030330
	GO:BP	morphogenesis of an epithelium	0.001670279	GO:0002009
	GO:BP	viral process	0.001670279	GO:0016032
	GO:CC	actomyosin	0.001936148	GO:0042641
	GO:CC	nucleus	0.003377454	GO:0005634
	GO:BP	protein localization to membrane	0.004059746	GO:0072657
	GO:BP	symbiotic process	0.004180378	GO:0044403
	GO:BP	regulation of cell junction assembly	0.004640343	GO:1901888
	GO:BP	cell-substrate junction assembly	0.005384431	GO:0007044
	GO:BP	cell-substrate junction organization	0.005384431	GO:0150115
	GO:BP	protein targeting	0.005992127	GO:0006605
	GO:CC	cytosolic small ribosomal subunit	0.006502547	GO:0022627
	GO:BP	morphogenesis of a branching structure	0.006726511	GO:0001763
	GO:BP	DNA damage response, signal transduction by p53 class mediator resulting in transcription of p21 class mediator	0.006726511	GO:0006978
	GO:BP	signal transduction in response to DNA damage	0.006729616	GO:0042770
	GO:CC	polysome	0.006747807	GO:0005844
	GO:CC	intracellular non-membrane-bounded organelle	0.006747807	GO:0043232
	GO:CC	intracellular organelle	0.006747807	GO:0043229

	GO:CC	non-membrane-bounded organelle	0.006941811	GO:0043228
	GO:BP	regulation of cell cycle arrest	0.007648848	GO:0071156
	GO:BP	tissue morphogenesis	0.007648848	GO:0048729
	GO:BP	DNA damage response, signal transduction resulting in transcription	0.007648848	GO:0042772
	GO:BP	interspecies interaction between organisms	0.007935545	GO:0044419
	GO:BP	DNA damage response, signal transduction by p53 class mediator resulting in cell cycle arrest	0.008021432	GO:0006977
	GO:BP	signal transduction involved in mitotic G1 DNA damage checkpoint	0.008415812	GO:0072431
	GO:BP	intracellular signal transduction involved in G1 DNA damage checkpoint	0.008415812	GO:1902400
	GO:BP	signal transduction involved in mitotic DNA integrity checkpoint	0.009499912	GO:1902403
	GO:BP	signal transduction involved in mitotic DNA damage checkpoint	0.009499912	GO:1902402
	GO:BP	signal transduction involved in mitotic cell cycle checkpoint	0.009499912	GO:0072413
	GO:BP	mRNA metabolic process	0.010817634	GO:0016071
	GO:BP	morphogenesis of a branching epithelium	0.011313054	GO:0061138
	GO:BP	mitotic G1 DNA damage checkpoint	0.012538087	GO:0031571
	GO:BP	mitotic G1/S transition checkpoint	0.012538087	GO:0044819
	GO:BP	G1 DNA damage checkpoint	0.012594474	GO:0044783
	GO:BP	lateral sprouting involved in lung morphogenesis	0.012594474	GO:0060490
	GO:BP	planar dichotomous subdivision of terminal units involved in lung branching morphogenesis	0.012594474	GO:0060489
	GO:BP	orthogonal dichotomous subdivision of terminal units involved in lung branching morphogenesis	0.012594474	GO:0060488
	GO:BP	lens morphogenesis in camera-type eye	0.013530386	GO:0002089
	GO:BP	positive regulation of signal transduction by p53 class mediator	0.013530386	GO:1901798
	GO:BP	macromolecule catabolic process	0.013530386	GO:0009057
	GO:BP	positive regulation of biosynthetic process	0.013530386	GO:0009891
	GO:BP	intracellular signal transduction	0.014809956	GO:0035556
	GO:CC	cytoplasm	0.015575713	GO:0005737
	GO:MF	transcription regulator activity	0.01563468	GO:0140110
	GO:MF	cation binding	0.01563468	GO:0043169
	GO:MF	ion binding	0.01563468	GO:0043167
	GO:MF	rRNA binding	0.01563468	GO:0019843
	GO:MF	metal ion binding	0.01563468	GO:0046872
	GO:BP	negative regulation of cell-substrate adhesion	0.01573706	GO:0010812
	GO:MF	nucleic acid binding	0.01603777	GO:0003676
	GO:MF	heterocyclic compound binding	0.01603777	GO:1901363
	GO:BP	protein localization to organelle	0.01710828	GO:0033365
	GO:BP	negative regulation of cell junction assembly	0.017341285	GO:1901889
	GO:BP	intrinsic apoptotic signaling pathway in response to DNA damage by p53 class mediator	0.017341285	GO:0042771
	GO:BP	protein localization	0.017404122	GO:0008104
	GO:CC	lamellipodium	0.017424103	GO:0030027

	GO:BP	nucleobase-containing compound catabolic process	0.017436379	GO:0034655
	GO:BP	RNA metabolic process	0.018120847	GO:0016070
	GO:BP	animal organ morphogenesis	0.019439466	GO:0009887
	GO:BP	positive regulation of macromolecule biosynthetic process	0.019439466	GO:0010557
	GO:BP	organelle organization	0.019439466	GO:0006996
	GO:BP	odontogenesis	0.019439466	GO:0042476
	GO:BP	cell adhesion	0.019439466	GO:0007155
	GO:BP	signal transduction involved in DNA integrity checkpoint	0.019439466	GO:0072401
	GO:BP	signal transduction involved in DNA damage checkpoint	0.019439466	GO:0072422
	GO:BP	signal transduction involved in cell cycle checkpoint	0.020000541	GO:0072395
	GO:BP	biological adhesion	0.020311338	GO:0022610
	GO:BP	intrinsic apoptotic signaling pathway in response to DNA damage	0.021189712	GO:0008630
	GO:CC	nucleoplasm	0.021883279	GO:0005654
	GO:MF	organic cyclic compound binding	0.022709457	GO:0097159
	GO:MF	DNA-binding transcription repressor activity, RNA polymerase II-specific	0.022709457	GO:0001227
	GO:MF	DNA-binding transcription repressor activity	0.022709457	GO:0001217
	GO:BP	regulation of DNA damage response, signal transduction by p53 class mediator resulting in transcription of p21 class mediator	0.024630154	GO:1902162
	GO:BP	positive regulation of DNA damage response, signal transduction by p53 class mediator resulting in transcription of p21 class mediator	0.024630154	GO:1902164
	GO:BP	intrinsic apoptotic signaling pathway by p53 class mediator	0.024630154	GO:0072332
	GO:BP	dichotomous subdivision of terminal units involved in lung branching	0.024630154	GO:0060448
	GO:BP	positive regulation of cellular biosynthetic process	0.024935254	GO:0031328
	GO:BP	lens development in camera-type eye	0.025549335	GO:0002088
	GO:BP	apical protein localization	0.02583715	GO:0045176
	GO:BP	lateral sprouting from an epithelium	0.02583715	GO:0060601
	GO:CC	adherens junction	0.026983411	GO:0005912
	GO:BP	positive regulation of cellular metabolic process	0.027525432	GO:0031325
	GO:BP	translation	0.027525432	GO:0006412
	GO:CC	nuclear lumen	0.029699566	GO:0031981
	GO:BP	cell-substrate adhesion	0.03050677	GO:0031589
	GO:BP	cellular component organization or biogenesis	0.03050677	GO:0071840
	GO:BP	focal adhesion assembly	0.030523143	GO:0048041
	GO:BP	epithelium development	0.030644858	GO:0060429
	GO:BP	organic cyclic compound catabolic process	0.030644858	GO:1901361
	GO:BP	tissue development	0.030727165	GO:0009888
	GO:BP	positive regulation of macromolecule metabolic process	0.030773697	GO:0010604
	GO:BP	heterocycle catabolic process	0.030773697	GO:0046700
	GO:BP	cellular nitrogen compound catabolic process	0.030773697	GO:0044270
	GO:BP	anatomical structure formation involved in morphogenesis	0.030773697	GO:0048646
	GO:BP	branching morphogenesis of an epithelial tube	0.031049805	GO:0048754
	GO:BP	cellular macromolecule catabolic process	0.031049805	GO:0044265

	GO:BP	morphogenesis of an epithelial sheet	0.032115062	GO:0002011
	GO:BP	negative regulation of transcription by RNA polymerase II	0.032195955	GO:0000122
	GO:BP	peptide biosynthetic process	0.034669286	GO:0043043
	GO:BP	positive regulation of metabolic process	0.034669286	GO:0009893
	GO:BP	aromatic compound catabolic process	0.035377654	GO:0019439
	GO:BP	establishment of body hair or bristle planar orientation	0.035377654	GO:0048104
	GO:BP	frontal suture morphogenesis	0.035377654	GO:0060364
	GO:BP	cell cycle arrest	0.035377654	GO:0007050
	GO:BP	establishment of body hair planar orientation	0.035377654	GO:0048105
	GO:BP	camera-type eye morphogenesis	0.035377654	GO:0048593
	GO:BP	regulation of cell adhesion	0.035377654	GO:0030155
	GO:BP	hair follicle development	0.035377654	GO:0001942
	GO:BP	positive regulation of nucleic acid-templated transcription	0.035613873	GO:1903508
	GO:BP	positive regulation of RNA biosynthetic process	0.035667706	GO:1902680
	GO:CC	CCR4-NOT core complex	0.035736658	GO:0030015
	GO:BP	positive regulation of DNA damage response, signal transduction by p53 class mediator	0.036097122	GO:0043517
	GO:BP	hair cycle process	0.037607372	GO:0022405
	GO:BP	skin epidermis development	0.037607372	GO:0098773
	GO:BP	molting cycle process	0.037607372	GO:0022404
	GO:BP	cellular localization	0.038406923	GO:0051641
	GO:BP	actin cytoskeleton organization	0.038746455	GO:0030036
	GO:BP	gland morphogenesis	0.038746455	GO:0022612
	GO:BP	negative regulation of gene expression	0.038746455	GO:0010629
	GO:BP	establishment of protein localization	0.038746455	GO:0045184
	GO:BP	megakaryocyte development	0.039147965	GO:0035855
	GO:BP	regulation of focal adhesion assembly	0.039147965	GO:0051893
	GO:BP	regulation of cell-substrate junction organization	0.039147965	GO:0150116
	GO:BP	protein neddylation	0.039147965	GO:0045116
	GO:BP	nucleic acid metabolic process	0.039147965	GO:0090304
	GO:BP	regulation of cell-substrate junction assembly	0.039147965	GO:0090109
	GO:CC	small ribosomal subunit	0.04158744	GO:0015935
	GO:BP	positive regulation of RNA metabolic process	0.041721054	GO:0051254
	GO:BP	kidney morphogenesis	0.042127538	GO:0060993
	GO:BP	cellular response to external stimulus	0.042152752	GO:0071496
	GO:BP	negative regulation of nucleic acid-templated transcription	0.042152752	GO:1903507
	GO:BP	regulation of intracellular signal transduction	0.042320682	GO:1902531
	GO:BP	negative regulation of RNA biosynthetic process	0.042320682	GO:1902679
	GO:BP	negative regulation of cell-substrate junction organization	0.042320682	GO:0150118
	GO:BP	negative regulation of focal adhesion assembly	0.042320682	GO:0051895
	GO:BP	regulation of gene expression	0.042320682	GO:0010468
	GO:BP	craniofacial suture morphogenesis	0.042320682	GO:0097094

	GO:BP	negative regulation of transcription, DNA-templated	0.043246248	GO:0045892
	GO:BP	cranial skeletal system development	0.043975282	GO:1904888
	GO:BP	regulation of mast cell cytokine production	0.044782657	GO:0032763
	GO:BP	cell migration involved in kidney development	0.044782657	GO:0035787
	GO:BP	post-anal tail morphogenesis	0.0479646	GO:0036342
	GO:BP	mitotic DNA damage checkpoint	0.049305797	GO:0044773
	KEGG	Ribosome	4.33392E-10	03010
	KEGG	Pathways in cancer	8.97096E-06	05200
	KEGG	Transcriptional misregulation in cancer	0.000365052	05202
	KEGG	MAPK signaling pathway	0.006498772	04010
	KEGG	MicroRNAs in cancer	0.007222518	05206
	KEGG	Human papillomavirus infection	0.009071854	05165
	KEGG	Axon guidance	0.009153114	04360
	KEGG	PI3K-Akt signaling pathway	0.010722825	04151
	KEGG	Insulin signaling pathway	0.014625987	04910
	KEGG	Herpes simplex virus 1 infection	0.016678246	05168
	KEGG	Small cell lung cancer	0.022100721	05222
	KEGG	C-type lectin receptor signaling pathway	0.031208175	04625
	KEGG	Insulin resistance	0.032876783	04931
**K5/6-only vs double-high**				
**Upregulated in the K5/6-only group**	**Source**	**Terms**	**FDR**	**Term ID**
	NA	NA	NA	NA
**Upregulated in the double-high group**	**Source**	**Terms**	**FDR**	**Term ID**
	GO:CC	intracellular	2.27263E-06	GO:0005622
	GO:CC	intracellular membrane-bounded organelle	1.39869E-05	GO:0043231
	GO:CC	intracellular organelle	1.62845E-05	GO:0043229
	GO:CC	membrane-bounded organelle	8.82335E-05	GO:0043227
	GO:CC	organelle	0.000176139	GO:0043226
	GO:CC	cytosolic ribosome	0.000458476	GO:0022626
	GO:CC	nucleus	0.000568222	GO:0005634
	GO:MF	DNA binding	0.002980091	GO:0003677
	GO:MF	nucleic acid binding	0.002980091	GO:0003676
	GO:CC	cytosolic small ribosomal subunit	0.010702776	GO:0022627
	GO:BP	cellular metabolic process	0.011395581	GO:0044237
	GO:BP	primary metabolic process	0.011395581	GO:0044238
	GO:BP	nucleobase-containing compound metabolic process	0.011395581	GO:0006139
	GO:BP	RNA metabolic process	0.011395581	GO:0016070
	GO:BP	nucleic acid metabolic process	0.011395581	GO:0090304
	GO:CC	ribosomal subunit	0.011798945	GO:0044391
	GO:BP	heterocycle metabolic process	0.012344657	GO:0046483
	GO:BP	organic cyclic compound metabolic process	0.012344657	GO:1901360
	GO:BP	cellular nitrogen compound metabolic process	0.012344657	GO:0034641

	GO:BP	cellular macromolecule metabolic process	0.012344657	GO:0044260
	GO:BP	nitrogen compound metabolic process	0.012344657	GO:0006807
	GO:BP	gene expression	0.012344657	GO:0010467
	GO:BP	cellular nitrogen compound biosynthetic process	0.012344657	GO:0044271
	GO:BP	cotranslational protein targeting to membrane	0.012344657	GO:0006613
	GO:BP	cellular aromatic compound metabolic process	0.012344657	GO:0006725
	GO:BP	SRP-dependent cotranslational protein targeting to membrane	0.012344657	GO:0006614
	GO:BP	protein targeting to ER	0.013654075	GO:0045047
	GO:BP	establishment of protein localization	0.014401259	GO:0045184
	GO:BP	regulation of cellular macromolecule biosynthetic process	0.014401259	GO:2000112
	GO:BP	establishment of protein localization to endoplasmic reticulum	0.014401259	GO:0072599
	GO:BP	regulation of macromolecule biosynthetic process	0.015691487	GO:0010556
	GO:BP	metabolic process	0.01696399	GO:0008152
	GO:BP	nuclear-transcribed mRNA catabolic process, nonsense-mediated decay	0.01696399	GO:0000184
	GO:BP	cellular macromolecule biosynthetic process	0.01696399	GO:0034645
	GO:CC	cell-substrate junction	0.016980193	GO:0030055
	GO:BP	macromolecule biosynthetic process	0.018628562	GO:0009059
	GO:BP	macromolecule metabolic process	0.021034342	GO:0043170
	GO:BP	cellular biosynthetic process	0.022212097	GO:0044249
	GO:BP	organic substance metabolic process	0.022887366	GO:0071704
	GO:CC	ribonucleoprotein complex	0.024727232	GO:1990904
	GO:BP	protein localization to endoplasmic reticulum	0.024854245	GO:0070972
	GO:BP	regulation of gene expression	0.024854245	GO:0010468
	GO:BP	viral gene expression	0.024854245	GO:0019080
	GO:BP	regulation of cellular biosynthetic process	0.024854245	GO:0031326
	GO:BP	translational initiation	0.024854245	GO:0006413
	GO:BP	establishment of protein localization to membrane	0.026317574	GO:0090150
	GO:BP	organic substance biosynthetic process	0.026425811	GO:1901576
	GO:BP	establishment of protein localization to organelle	0.032053263	GO:0072594
	GO:BP	regulation of biosynthetic process	0.032063061	GO:0009889
	GO:BP	nuclear-transcribed mRNA catabolic process	0.033457136	GO:0000956
	GO:BP	biosynthetic process	0.037110883	GO:0009058
	GO:BP	protein transport	0.037110883	GO:0015031
	GO:CC	ribosome	0.037356381	GO:0005840
	GO:BP	protein targeting	0.038369506	GO:0006605
	GO:BP	regulation of nucleic acid-templated transcription	0.041287999	GO:1903506
	GO:BP	regulation of RNA biosynthetic process	0.041455128	GO:2001141
	GO:BP	regulation of RNA metabolic process	0.042136142	GO:0051252
	GO:BP	regulation of nucleobase-containing compound metabolic process	0.042136142	GO:0019219
	GO:BP	peptide transport	0.042373576	GO:0015833
	GO:BP	nucleic acid-templated transcription	0.042373576	GO:0097659
	GO:BP	regulation of macromolecule metabolic process	0.042373576	GO:0060255

	GO:CC	focal adhesion	0.042839885	GO:0005925
	GO:CC	small ribosomal subunit	0.042839885	GO:0015935
	GO:CC	nuclear lumen	0.043041852	GO:0031981
	GO:CC	intracellular organelle lumen	0.043041852	GO:0070013
	GO:CC	organelle lumen	0.043041852	GO:0043233
	GO:CC	membrane-enclosed lumen	0.043041852	GO:0031974
	GO:BP	RNA biosynthetic process	0.044770599	GO:0032774
	GO:BP	regulation of transcription, DNA-templated	0.04698976	GO:0006355
	GO:BP	cellular macromolecule catabolic process	0.048193499	GO:0044265
	GO:BP	amide transport	0.048193499	GO:0042886
	KEGG	Herpes simplex virus 1 infection	5.54331E-08	05168
	KEGG	Ribosome	0.002999276	03010
**K20-only vs double-low**				
**Upregulated in the K20-only group**	**Source**	**Terms**	**FDR**	**Term ID**
	GO:BP	positive regulation of neuron differentiation	0.04060163	GO:0045666
	KEGG	Metabolic pathways	0.011294001	01100
	KEGG	Prostate cancer	0.029679874	05215
**Upregulated in the double-low group**	**Source**	**Terms**	**FDR**	**Term ID**
	GO:BP	positive regulation of macromolecule metabolic process	0.000519216	GO:0010604
	GO:BP	positive regulation of nitrogen compound metabolic process	0.000519216	GO:0051173
	GO:BP	positive regulation of metabolic process	0.001288851	GO:0009893
	GO:BP	positive regulation of cellular metabolic process	0.002914289	GO:0031325
	GO:BP	positive regulation of cellular process	0.018413638	GO:0048522
	GO:BP	positive regulation of macromolecule biosynthetic process	0.018413638	GO:0010557
	GO:BP	positive regulation of RNA metabolic process	0.018413638	GO:0051254
	GO:BP	positive regulation of biosynthetic process	0.025136145	GO:0009891
	GO:BP	positive regulation of nucleic acid-templated transcription	0.025136145	GO:1903508
	GO:BP	tripeptide transmembrane transport	0.025136145	GO:0035443
	GO:BP	positive regulation of biological process	0.025136145	GO:0048518
	GO:BP	developmental process	0.025136145	GO:0032502
	GO:BP	positive regulation of nucleobase-containing compound metabolic process	0.025136145	GO:0045935
	GO:BP	glutathione transmembrane transport	0.025136145	GO:0034775
	GO:BP	positive regulation of cellular biosynthetic process	0.025136145	GO:0031328
	GO:BP	response to mechanical stimulus	0.025136145	GO:0009612
	GO:BP	positive regulation of RNA biosynthetic process	0.025136145	GO:1902680
	GO:BP	intracellular signal transduction	0.030008141	GO:0035556
	GO:BP	apoptotic process	0.030008141	GO:0006915
	GO:BP	lens development in camera-type eye	0.030812786	GO:0002088
	GO:BP	multicellular organismal process	0.030812786	GO:0032501
	GO:MF	DNA-binding transcription activator activity	0.031427378	GO:0001216
	GO:MF	protein binding	0.031427378	GO:0005515
	GO:MF	cell adhesion molecule binding	0.031427378	GO:0050839

	GO:MF	DNA-binding transcription activator activity, RNA polymerase II-specific	0.031427378	GO:0001228
	GO:BP	tripeptide transport	0.035449183	GO:0042939
	GO:BP	positive regulation of gene expression	0.035449183	GO:0010628
	GO:BP	glutathione transport	0.035449183	GO:0034635
	GO:MF	transcription regulatory region DNA binding	0.036862541	GO:0044212
	GO:MF	regulatory region nucleic acid binding	0.036862541	GO:0001067
	GO:BP	odontogenesis	0.037122886	GO:0042476
	GO:BP	multicellular organism development	0.037122886	GO:0007275
	GO:BP	regulation of nitrogen compound metabolic process	0.037122886	GO:0051171
	GO:MF	chromatin binding	0.040320043	GO:0003682
	GO:BP	regulation of primary metabolic process	0.044532289	GO:0080090
	GO:BP	odontogenesis of dentin-containing tooth	0.045353004	GO:0042475
	GO:BP	animal organ morphogenesis	0.049500257	GO:0009887
**K5/6-only vs double-low**				
**Upregulated in the K5/6-only group**	**Source**	**Terms**	**FDR**	**Term ID**
	GO:BP	regulation of axon extension involved in axon guidance	0.016989629	GO:0048841
	GO:BP	tissue morphogenesis	0.016989629	GO:0048729
	GO:BP	positive regulation of axon extension involved in axon guidance	0.016989629	GO:0048842
	GO:BP	regulation of axon guidance	0.016989629	GO:1902667
	GO:BP	axon extension involved in axon guidance	0.016989629	GO:0048846
	GO:BP	neuron projection extension involved in neuron projection guidance	0.016989629	GO:1902284
	GO:BP	mesenchymal stem cell differentiation	0.016989629	GO:0072497
	GO:BP	positive regulation of axon guidance	0.016989629	GO:1902669
	GO:BP	biological adhesion	0.020902314	GO:0022610
	GO:BP	positive regulation of retinal ganglion cell axon guidance	0.020902314	GO:1902336
	GO:BP	VEGF-activated neuropilin signaling pathway	0.020902314	GO:0038190
	GO:BP	cell adhesion	0.020902314	GO:0007155
	GO:BP	tissue development	0.020902314	GO:0009888
	GO:BP	regulation of cell communication	0.020902314	GO:0010646
	GO:BP	epithelium development	0.021809296	GO:0060429
	GO:BP	positive regulation of cell-matrix adhesion	0.021809296	GO:0001954
	GO:BP	regulation of signaling	0.021809296	GO:0023051
	GO:BP	endoderm formation	0.02210452	GO:0001706
	GO:BP	morphogenesis of an epithelium	0.024019519	GO:0002009
	GO:BP	regulation of anatomical structure morphogenesis	0.024094475	GO:0022603
	GO:BP	formation of primary germ layer	0.024094475	GO:0001704
	GO:BP	endothelium development	0.026558932	GO:0003158
	GO:BP	branching involved in blood vessel morphogenesis	0.026585883	GO:0001569
	GO:BP	epithelial tube morphogenesis	0.027338061	GO:0060562
	GO:BP	fasciculation of sensory neuron axon	0.03223172	GO:0097155
	GO:BP	gastrulation	0.039835193	GO:0007369
	GO:BP	cell-substrate adhesion	0.043123421	GO:0031589

	GO:BP	endothelial cell differentiation	0.048035276	GO:0045446
	GO:BP	branching morphogenesis of an epithelial tube	0.048035276	GO:0048754
	GO:BP	neuropilin signaling pathway	0.048035276	GO:0038189
	GO:BP	positive regulation of mesenchymal stem cell differentiation	0.048035276	GO:2000741
	GO:BP	tube development	0.048035276	GO:0035295
	GO:BP	cell migration	0.048035276	GO:0016477
	GO:BP	multicellular organismal homeostasis	0.048035276	GO:0048871
	GO:BP	positive regulation of cellular process	0.048035276	GO:0048522
	GO:BP	Rho protein signal transduction	0.048035276	GO:0007266
	GO:BP	anatomical structure formation involved in morphogenesis	0.048035276	GO:0048646
	KEGG	Regulation of actin cytoskeleton	0.005476046	04810
	KEGG	Breast cancer	0.005539287	05224
	KEGG	Proteoglycans in cancer	0.014267891	05205
	KEGG	Human papillomavirus infection	0.014267891	05165
	KEGG	FoxO signaling pathway	0.01876374	04068
	KEGG	Pathways in cancer	0.01876374	05200
	KEGG	Phospholipase D signaling pathway	0.01876374	04072
	KEGG	Gastric cancer	0.01876374	05226
	KEGG	Renal cell carcinoma	0.020561804	05211
	KEGG	Hepatocellular carcinoma	0.023164748	05225
	KEGG	Axon guidance	0.027515425	04360
	KEGG	Focal adhesion	0.035352986	04510
	KEGG	Insulin resistance	0.044517181	04931
	KEGG	HIF-1 signaling pathway	0.044517181	04066
	KEGG	Circadian rhythm	0.044517181	04710
**Upregulated in the double-low group**	**Source**	**Terms**	**FDR**	**Term ID**
	GO:CC	intracellular	1.13859E-10	GO:0005622
	GO:CC	intracellular membrane-bounded organelle	6.41041E-10	GO:0043231
	GO:CC	intracellular organelle	1.64612E-07	GO:0043229
	GO:CC	nucleus	1.64612E-07	GO:0005634
	GO:CC	membrane-bounded organelle	3.88369E-07	GO:0043227
	GO:CC	organelle	7.55082E-06	GO:0043226
	GO:CC	nuclear lumen	0.000272054	GO:0031981
	GO:CC	organelle lumen	0.001168715	GO:0043233
	GO:CC	membrane-enclosed lumen	0.001168715	GO:0031974
	GO:CC	intracellular organelle lumen	0.001168715	GO:0070013
	GO:CC	cytosol	0.001622371	GO:0005829
	GO:CC	nucleoplasm	0.002414275	GO:0005654
	GO:CC	ribonucleoprotein complex	0.003519478	GO:1990904
	GO:CC	midbody	0.003629366	GO:0030496
	GO:CC	cyclin B1-CDK1 complex	0.003629366	GO:0097125
	GO:CC	cytoplasm	0.00455025	GO:0005737

	GO:BP	intracellular transport	0.007765366	GO:0046907
	GO:BP	cellular metabolic process	0.007765366	GO:0044237
	GO:BP	primary metabolic process	0.008266757	GO:0044238
	GO:BP	intrinsic apoptotic signaling pathway	0.010408129	GO:0097193
	GO:BP	metabolic process	0.010408129	GO:0008152
	GO:BP	organic substance metabolic process	0.014495254	GO:0071704
	GO:BP	cellular response to stress	0.015229771	GO:0033554
	GO:BP	macromolecule metabolic process	0.015229771	GO:0043170
	GO:BP	nitrogen compound metabolic process	0.015229771	GO:0006807
	GO:BP	cellular macromolecule metabolic process	0.015229771	GO:0044260
	GO:BP	nucleobase-containing compound metabolic process	0.015259809	GO:0006139
	GO:BP	regulation of intrinsic apoptotic signaling pathway	0.018772491	GO:2001242
	GO:BP	intrinsic apoptotic signaling pathway in response to DNA damage	0.028267316	GO:0008630
	GO:BP	regulation of RNA splicing	0.028583353	GO:0043484
	GO:BP	cellular nitrogen compound metabolic process	0.028583353	GO:0034641
	GO:BP	mitotic cell cycle process	0.029225861	GO:1903047
	GO:BP	heterocycle metabolic process	0.031592498	GO:0046483
	GO:BP	positive regulation of protein oligomerization	0.031592498	GO:0032461
	GO:BP	cellular aromatic compound metabolic process	0.037872095	GO:0006725
	GO:CC	organelle envelope	0.038785841	GO:0031967
	GO:CC	envelope	0.038785841	GO:0031975
	GO:BP	cell cycle	0.040809838	GO:0007049
	GO:BP	cell cycle process	0.040809838	GO:0022402
	GO:BP	bicellular tight junction assembly	0.040809838	GO:0070830
	GO:BP	nucleic acid metabolic process	0.040809838	GO:0090304
	GO:BP	organic cyclic compound metabolic process	0.040809838	GO:1901360
	GO:BP	cellular response to DNA damage stimulus	0.040809838	GO:0006974
	GO:BP	mitotic cell cycle	0.043265395	GO:0000278
	GO:BP	G2/M transition of mitotic cell cycle	0.044516062	GO:0000086
	GO:BP	cell cycle phase transition	0.044516062	GO:0044770
	GO:BP	tight junction assembly	0.04475717	GO:0120192
	GO:CC	neuron projection cytoplasm	0.048931405	GO:0120111
	KEGG	Metabolic pathways	2.56255E-05	01100
	KEGG	Cell cycle	0.012725543	04110
	KEGG	p53 signaling pathway	0.012725543	04115
	KEGG	Ubiquitin mediated proteolysis	0.012725543	04120
	KEGG	DNA replication	0.018514438	03030
	KEGG	mRNA surveillance pathway	0.018514438	03015
	KEGG	Herpes simplex virus 1 infection	0.026012066	05168
	KEGG	Sphingolipid signaling pathway	0.036284137	04071

Table S3. Class annotation to the UROMOL intrinsic subtypes by machine learning.

Sample	Sparse Partial Least Squares (accuracy = 0.944)	Regularized Logistic Regression (accuracy = 0.943)	GLMnet (accuracy = 0.941)
K5/6-only 1	class_3	class_3	class_3
K5/6-only 2	class_3	class_2	class_3
K5/6-only 3	class_3	class_3	class_3
K5/6-only 4	class_3	class_3	class_3
K20-only 1	class_2	class_2	class_2
K20-only 2	class_2	class_2	class_2
K20-only 3	class_2	class_2	class_2
K20-only 4	class_2	class_2	class_2
Double-high 1	class_3	class_3	class_3
Double-high 2	class_3	class_2	class_3
Double-high 3	class_3	class_3	class_3
Double-high 4	class_3	class_2	class_3
Double-low 1	class_3	class_2	class_2
Double-low 2	class_2	class_2	class_2
Double-low 3	class_3	class_3	class_3
Double-low 4	class_3	class_3	class_3

Table S4. Clinicopathological details of the Lund cohort groups.

Group	K5/6-only_Lund (n = 10)	K20-only_Lund (n = 3)	Double-high_Lund (n = 4)	Double-low_Lund (n = 6)	P[a]
Age, median (range)	78 years (63-87)	74 years (60-77)	69.5 years (66-75)	78 years (52-86)	0.350
Sex					0.153
Female	4 (40.0%)	1 (33.3%)	0 (0.0%)	0 (0.0%)	
Male	6 (60.0%)	2 (66.7%)	4 (100.0%)	6 (100.0%)	
Stromal invasion (T stage)					1.000
Present (T1)	3 (30.0%)	1 (33.3%)	1 (25.0%)	2 (33.3%)	
Absent (Ta)	7 (70.0%)	2 (66.7%)	3 (75.0%)	4 (66.7%)	
WHO grade					0.095
Grade 3	2 (20.0%)	2 (66.7%)	0 (0.0%)	0 (0.0%)	
Grade 2	8 (80.0%)	1 (33.3%)	4 (100.0%)	6 (100.0%)	
Molecular subtype					0.080
Urothelial-like A	8 (80.0%)	1 (33.3%)	4 (100.0%)	6 (100.0%)	
Urothelial-like B	2 (20.0%)	0 (0.0%)	0 (0.0%)	0 (0.0%)	
Genomically Unstable	0 (0.0%)	1 (33.3%)	0 (0.0%)	0 (0.0%)	
SCC-like	0 (0.0%)	1 (33.3%)	0 (0.0%)	0 (0.0%)	
Immunohistochemistry score					
K5/6[b] (mean ± S.D.)	2.9 ± 1.1	0.9 ± 0.3	2.4 ± 0.5	0.7 ± 0.5	<0.001
K20 (mean ± S.D.)	0.0 ± 0.0	2.0 ± 0.0	1.9 ± 0.1	0.0 ± 0.0	<0.001
K14 (mean ± S.D.)	0.4 ± 0.6	0.0 ± 0.0	0.7 ± 0.4	0.0 ± 0.0	0.145
Ki-67 (mean ± S.D.)	0.1 ± 0.1	0.3 ± 0.1	0.1 ± 0.1	0.2 ± 0.1	0.044
E2F3 (mean ± S.D.)	0.2 ± 0.1	0.9 ± 0.5	0.4 ± 0.2	0.2 ± 0.2	0.001

[a]: Pearson's chi-squared or Fisher's exact tests for categorical variables; Mann-Whitney U test for immunohistochemistry, b: K5 score + K6 score

Table S5. GO and KEGG analyses of the Lund cohort.

**K20-only_Lund vs K5/6-only_Lund**				
**Upregulated in the K20-only_Lund group**	**Source**	**Terms**	**FDR**	**Term ID**
	GO:BP	cell cycle	1.61326E-34	GO:0007049
	GO:BP	mitotic cell cycle process	3.79593E-34	GO:1903047
	GO:BP	cell cycle process	1.04742E-32	GO:0022402
	GO:BP	mitotic cell cycle	1.04742E-32	GO:0000278
	GO:BP	cell division	3.46222E-31	GO:0051301
	GO:BP	chromosome segregation	9.00562E-28	GO:0007059
	GO:BP	mitotic nuclear division	1.23047E-27	GO:0140014
	GO:BP	sister chromatid segregation	3.21127E-27	GO:0000819
	GO:BP	nuclear chromosome segregation	8.68222E-27	GO:0098813
	GO:BP	chromosome organization	2.40096E-26	GO:0051276
	GO:BP	mitotic sister chromatid segregation	4.3679E-26	GO:0000070
	GO:BP	nuclear division	7.03824E-26	GO:0000280
	GO:BP	organelle fission	4.72889E-24	GO:0048285
	GO:BP	regulation of cell cycle	3.18716E-19	GO:0051726
	GO:CC	spindle	5.93428E-19	GO:0005819
	GO:CC	chromosome, centromeric region	2.31636E-18	GO:0000775
	GO:BP	organelle organization	8.05338E-18	GO:0006996
	GO:CC	chromosomal region	8.99634E-18	GO:0098687
	GO:BP	cell cycle phase transition	1.46601E-17	GO:0044770
	GO:BP	mitotic cell cycle phase transition	4.88133E-17	GO:0044772
	GO:CC	chromosome	7.89841E-17	GO:0005694
	GO:BP	microtubule cytoskeleton organization involved in mitosis	4.62656E-16	GO:1902850
	GO:CC	microtubule cytoskeleton	1.38829E-15	GO:0015630
	GO:CC	kinetochore	1.47272E-15	GO:0000776
	GO:CC	condensed chromosome, centromeric region	1.47272E-15	GO:0000779
	GO:CC	condensed chromosome	1.47272E-15	GO:0000793
	GO:BP	regulation of mitotic nuclear division	2.67725E-15	GO:0007088
	GO:BP	regulation of cell cycle process	5.52675E-15	GO:0010564
	GO:BP	regulation of chromosome segregation	9.55387E-15	GO:0051983
	GO:BP	mitotic spindle organization	1.56841E-14	GO:0007052
	GO:BP	regulation of mitotic cell cycle	1.90864E-14	GO:0007346
	GO:BP	regulation of nuclear division	4.17662E-14	GO:0051783
	GO:CC	intracellular non-membrane-bounded organelle	1.70805E-13	GO:0043232
	GO:CC	non-membrane-bounded organelle	1.79513E-13	GO:0043228
	GO:CC	intracellular	2.05188E-13	GO:0005622
	GO:CC	cytosol	3.1872E-13	GO:0005829
	GO:CC	condensed chromosome kinetochore	4.64943E-13	GO:0000777
	GO:BP	spindle organization	6.58619E-13	GO:0007051
	GO:CC	nucleus	1.68089E-12	GO:0005634

	GO:BP	metaphase plate congression	1.95642E-12	GO:0051310
	GO:BP	DNA metabolic process	2.12178E-12	GO:0006259
	GO:BP	microtubule cytoskeleton organization	4.11351E-12	GO:0000226
	GO:BP	establishment of chromosome localization	4.24916E-12	GO:0051303
	GO:BP	chromosome localization	5.01546E-12	GO:0050000
	GO:CC	spindle pole	5.05189E-12	GO:0000922
	GO:CC	nucleoplasm	7.28151E-12	GO:0005654
	GO:BP	regulation of sister chromatid segregation	1.51688E-11	GO:0033045
	GO:CC	centrosome	2.41533E-11	GO:0005813
	GO:CC	organelle	2.41533E-11	GO:0043226
	GO:BP	microtubule-based process	3.6491E-11	GO:0007017
	GO:CC	nuclear lumen	6.11905E-11	GO:0031981
	GO:CC	mitotic spindle	6.11905E-11	GO:0072686
	GO:BP	regulation of mitotic sister chromatid separation	6.14942E-11	GO:0010965
	GO:CC	microtubule organizing center	6.46303E-11	GO:0005815
	GO:CC	intracellular organelle	1.13375E-10	GO:0043229
	GO:BP	mitotic sister chromatid separation	1.14189E-10	GO:0051306
	GO:BP	regulation of chromosome separation	1.6771E-10	GO:1905818
	GO:BP	negative regulation of mitotic sister chromatid separation	1.92437E-10	GO:2000816
	GO:CC	midbody	1.9918E-10	GO:0030496
	GO:BP	negative regulation of mitotic nuclear division	2.09296E-10	GO:0045839
	GO:CC	cytoskeleton	2.09463E-10	GO:0005856
	GO:CC	membrane-bounded organelle	2.13454E-10	GO:0043227
	GO:BP	regulation of cell cycle phase transition	2.16651E-10	GO:1901987
	GO:CC	intracellular membrane-bounded organelle	2.22786E-10	GO:0043231
	GO:BP	cellular response to DNA damage stimulus	2.26297E-10	GO:0006974
	GO:BP	negative regulation of chromosome separation	2.32127E-10	GO:1905819
	GO:BP	mitotic cell cycle checkpoint	2.79271E-10	GO:0007093
	GO:BP	positive regulation of cell cycle process	2.82667E-10	GO:0090068
	GO:BP	cell cycle checkpoint	3.16923E-10	GO:0000075
	GO:BP	negative regulation of mitotic sister chromatid segregation	3.70002E-10	GO:0033048
	GO:BP	regulation of mitotic sister chromatid segregation	4.15583E-10	GO:0033047
	GO:BP	regulation of mitotic metaphase/anaphase transition	4.19129E-10	GO:0030071
	GO:BP	nuclear DNA replication	5.09399E-10	GO:0033260
	GO:BP	mitotic metaphase plate congression	5.65585E-10	GO:0007080
	GO:BP	negative regulation of sister chromatid segregation	5.65585E-10	GO:0033046
	GO:BP	regulation of metaphase/anaphase transition of cell cycle	5.80485E-10	GO:1902099
	GO:BP	metaphase/anaphase transition of mitotic cell cycle	5.80485E-10	GO:0007091
	GO:BP	negative regulation of chromosome segregation	6.75337E-10	GO:0051985
	GO:BP	chromosome separation	6.75337E-10	GO:0051304
	GO:BP	regulation of mitotic cell cycle phase transition	7.4292E-10	GO:1901990
	GO:BP	metaphase/anaphase transition of cell cycle	8.13103E-10	GO:0044784

	GO:BP	DNA packaging	8.59452E-10	GO:0006323
	GO:BP	negative regulation of nuclear division	9.42208E-10	GO:0051784
	GO:BP	DNA conformation change	9.42208E-10	GO:0071103
	GO:BP	cell cycle DNA replication	1.06333E-09	GO:0044786
	GO:BP	spindle checkpoint	1.06333E-09	GO:0031577
	GO:BP	spindle assembly checkpoint	1.06333E-09	GO:0071173
	GO:BP	mitotic spindle assembly checkpoint	1.06333E-09	GO:0007094
	GO:BP	mitotic spindle checkpoint	1.06333E-09	GO:0071174
	GO:CC	membrane-enclosed lumen	1.08294E-09	GO:0031974
	GO:CC	organelle lumen	1.08294E-09	GO:0043233
	GO:CC	intracellular organelle lumen	1.08294E-09	GO:0070013
	GO:CC	replication fork	1.17608E-09	GO:0005657
	GO:BP	positive regulation of cell cycle	1.21353E-09	GO:0045787
	GO:BP	regulation of chromosome organization	1.74452E-09	GO:0033044
	GO:BP	negative regulation of mitotic metaphase/anaphase transition	1.81303E-09	GO:0045841
	GO:BP	DNA replication	2.02151E-09	GO:0006260
	GO:BP	cytokinesis	2.28752E-09	GO:0000910
	GO:BP	negative regulation of metaphase/anaphase transition of cell cycle	2.28752E-09	GO:1902100
	GO:BP	negative regulation of cell cycle	3.60357E-09	GO:0045786
	GO:BP	cellular component organization	4.34824E-09	GO:0016043
	GO:BP	cellular response to stress	4.82226E-09	GO:0033554
	GO:BP	DNA recombination	4.97437E-09	GO:0006310
	GO:BP	mitotic spindle assembly	5.61469E-09	GO:0090307
	GO:BP	cellular component organization or biogenesis	6.03072E-09	GO:0071840
	GO:BP	attachment of spindle microtubules to kinetochore	1.1466E-08	GO:0008608
	GO:BP	negative regulation of mitotic cell cycle	1.26042E-08	GO:0045930
	GO:CC	condensed nuclear chromosome, centromeric region	1.68102E-08	GO:0000780
	GO:BP	DNA repair	1.81296E-08	GO:0006281
	GO:BP	negative regulation of chromosome organization	2.19463E-08	GO:2001251
	GO:BP	spindle assembly	3.81731E-08	GO:0051225
	GO:CC	spindle microtubule	3.86241E-08	GO:0005876
	GO:BP	cell cycle G1/S phase transition	4.85197E-08	GO:0044843
	GO:BP	organelle localization	7.80016E-08	GO:0051640
	GO:CC	microtubule associated complex	7.87405E-08	GO:0005875
	GO:BP	DNA replication initiation	8.19305E-08	GO:0006270
	GO:BP	meiotic cell cycle	1.04934E-07	GO:0051321
	GO:BP	establishment of organelle localization	1.0807E-07	GO:0051656
	GO:BP	positive regulation of mitotic cell cycle	1.09411E-07	GO:0045931
	GO:CC	cytoplasm	1.25386E-07	GO:0005737
	GO:CC	nuclear chromosome	1.46898E-07	GO:0000228
	GO:BP	G2/M transition of mitotic cell cycle	1.56758E-07	GO:0000086
	GO:BP	protein localization to chromosome, centromeric region	1.74071E-07	GO:0071459

	GO:BP	DNA-dependent DNA replication	2.63874E-07	GO:0006261
	GO:CC	kinesin complex	3.08712E-07	GO:0005871
	GO:BP	regulation of cytokinesis	4.15172E-07	GO:0032465
	GO:BP	cell cycle G2/M phase transition	4.32119E-07	GO:0044839
	GO:CC	condensed nuclear chromosome kinetochore	4.51331E-07	GO:0000778
	GO:BP	G1/S transition of mitotic cell cycle	4.52189E-07	GO:0000082
	GO:CC	condensed nuclear chromosome	4.65382E-07	GO:0000794
	GO:CC	supramolecular complex	5.68662E-07	GO:0099080
	GO:CC	microtubule	7.19859E-07	GO:0005874
	GO:BP	meiotic cell cycle process	7.31043E-07	GO:1903046
	GO:BP	mitotic cytokinesis	8.53984E-07	GO:0000281
	GO:BP	cytokinetic process	8.53984E-07	GO:0032506
	GO:BP	protein-DNA complex assembly	8.66262E-07	GO:0065004
	GO:BP	establishment of localization in cell	1.0585E-06	GO:0051649
	GO:BP	telomere maintenance via semi-conservative replication	1.12087E-06	GO:0032201
	GO:BP	mitotic spindle elongation	1.28712E-06	GO:0000022
	GO:CC	chromosome passenger complex	1.30743E-06	GO:0032133
	GO:BP	protein localization to kinetochore	1.40344E-06	GO:0034501
	GO:BP	regulation of organelle organization	1.43186E-06	GO:0033043
	GO:BP	protein-DNA complex subunit organization	1.46825E-06	GO:0071824
	GO:CC	protein-containing complex	1.55812E-06	GO:0032991
	GO:BP	cytoskeleton-dependent cytokinesis	1.77024E-06	GO:0061640
	GO:BP	spindle elongation	2.42587E-06	GO:0051231
	GO:BP	chromosome condensation	2.52886E-06	GO:0030261
	GO:CC	protein-DNA complex	3.11002E-06	GO:0032993
	GO:BP	regulation of cell cycle G2/M phase transition	3.24437E-06	GO:1902749
	GO:BP	positive regulation of cell cycle phase transition	4.14818E-06	GO:1901989
	GO:BP	telomere maintenance	4.26161E-06	GO:0000723
	GO:BP	regulation of cell division	5.22851E-06	GO:0051302
	GO:CC	condensed chromosome outer kinetochore	5.33099E-06	GO:0000940
	GO:BP	double-strand break repair via homologous recombination	5.45012E-06	GO:0000724
	GO:BP	recombinational repair	5.84383E-06	GO:0000725
	GO:BP	nucleosome assembly	6.26234E-06	GO:0006334
	GO:BP	regulation of G2/M transition of mitotic cell cycle	7.12167E-06	GO:0010389
	GO:BP	positive regulation of mitotic cell cycle phase transition	7.46363E-06	GO:1901992
	GO:BP	negative regulation of organelle organization	7.46363E-06	GO:0010639
	GO:BP	cellular aromatic compound metabolic process	8.22322E-06	GO:0006725
	GO:BP	organic cyclic compound metabolic process	8.44106E-06	GO:1901360
	GO:CC	nuclear replication fork	8.75433E-06	GO:0043596
	GO:BP	cytoskeleton organization	8.7673E-06	GO:0007010
	GO:BP	negative regulation of cell cycle phase transition	8.7673E-06	GO:1901988
	GO:BP	telomere organization	9.37611E-06	GO:0032200

	GO:BP	antigen processing and presentation of exogenous peptide antigen via MHC class II	9.50811E-06	GO:0019886
	GO:BP	meiotic nuclear division	9.84621E-06	GO:0140013
	GO:BP	heterocycle metabolic process	1.01881E-05	GO:0046483
	GO:BP	actomyosin contractile ring assembly	1.02234E-05	GO:0000915
	GO:BP	assembly of actomyosin apparatus involved in cytokinesis	1.02234E-05	GO:0000912
	GO:BP	chromatin organization	1.06212E-05	GO:0006325
	GO:BP	histone phosphorylation	1.0953E-05	GO:0016572
	GO:BP	antigen processing and presentation of peptide antigen via MHC class II	1.32137E-05	GO:0002495
	GO:BP	nucleosome organization	1.45453E-05	GO:0034728
	GO:BP	regulation of attachment of spindle microtubules to kinetochore	1.48079E-05	GO:0051988
	GO:BP	antigen processing and presentation of peptide or polysaccharide antigen via MHC class II	1.55501E-05	GO:0002504
	GO:BP	negative regulation of cell cycle process	1.55719E-05	GO:0010948
	GO:BP	regulation of centrosome cycle	1.55719E-05	GO:0046605
	GO:BP	negative regulation of mitotic cell cycle phase transition	1.66948E-05	GO:1901991
	GO:BP	chromatin assembly or disassembly	1.77699E-05	GO:0006333
	GO:CC	cell	1.82698E-05	GO:0005623
	GO:CC	centralspindlin complex	1.93818E-05	GO:0097149
	GO:BP	actomyosin contractile ring organization	2.15542E-05	GO:0044837
	GO:BP	mitotic spindle midzone assembly	2.15542E-05	GO:0051256
	GO:BP	chromatin assembly	2.28466E-05	GO:0031497
	GO:BP	anaphase-promoting complex-dependent catabolic process	2.32739E-05	GO:0031145
	GO:BP	regulation of transferase activity	2.80619E-05	GO:0051338
	GO:BP	protein localization to chromosome	2.81335E-05	GO:0034502
	GO:CC	spindle midzone	3.18595E-05	GO:0051233
	GO:CC	condensed nuclear chromosome outer kinetochore	7.37003E-05	GO:0000942
	GO:BP	centrosome cycle	9.33431E-05	GO:0007098
	GO:BP	DNA replication-independent nucleosome assembly	0.000101549	GO:0006336
	GO:BP	nucleobase-containing compound metabolic process	0.000103638	GO:0006139
	GO:CC	cyclin-dependent protein kinase holoenzyme complex	0.000110085	GO:0000307
	GO:BP	DNA replication-independent nucleosome organization	0.000113867	GO:0034724
	GO:BP	spindle midzone assembly	0.000117862	GO:0051255
	GO:BP	Golgi vesicle transport	0.000123327	GO:0048193
	GO:BP	cellular localization	0.000125562	GO:0051641
	GO:BP	centromere complex assembly	0.00012565	GO:0034508
	GO:BP	double-strand break repair	0.000126298	GO:0006302
	GO:BP	kinetochore organization	0.000154706	GO:0051383
	GO:MF	adenyl nucleotide binding	0.000155109	GO:0030554
	GO:MF	histone kinase activity	0.000155109	GO:0035173
	GO:MF	tubulin binding	0.000155109	GO:0015631
	GO:MF	adenyl ribonucleotide binding	0.000155109	GO:0032559

	GO:MF	catalytic activity	0.000155109	GO:0003824
	GO:MF	ATP binding	0.000155109	GO:0005524
	GO:MF	microtubule motor activity	0.000155109	GO:0003777
	GO:MF	microtubule binding	0.000155109	GO:0008017
	GO:BP	organelle assembly	0.000155166	GO:0070925
	GO:BP	microtubule organizing center organization	0.000161733	GO:0031023
	GO:CC	DNA packaging complex	0.000165178	GO:0044815
	GO:CC	alpha DNA polymerase:primase complex	0.000168828	GO:0005658
	GO:CC	condensed chromosome inner kinetochore	0.000168828	GO:0000939
	GO:BP	double-strand break repair via break-induced replication	0.000173442	GO:0000727
	GO:BP	positive regulation of mitotic nuclear division	0.000173442	GO:0045840
	GO:BP	positive regulation of protein localization to kinetochore	0.000180103	GO:1905342
	GO:BP	regulation of protein localization to kinetochore	0.000180103	GO:1905340
	GO:CC	intercellular bridge	0.000248098	GO:0045171
	GO:BP	antigen processing and presentation of exogenous peptide antigen	0.000251537	GO:0002478
	GO:BP	CENP-A containing nucleosome assembly	0.000330521	GO:0034080
	GO:BP	CENP-A containing chromatin organization	0.000330521	GO:0061641
	GO:BP	antigen processing and presentation of exogenous antigen	0.000358783	GO:0019884
	GO:BP	cellular nitrogen compound metabolic process	0.000361292	GO:0034641
	GO:BP	positive regulation of G2/M transition of mitotic cell cycle	0.000417805	GO:0010971
	GO:BP	meiotic chromosome segregation	0.000425306	GO:0045132
	GO:MF	ATPase activity	0.000444043	GO:0016887
	GO:BP	meiosis I cell cycle process	0.000449572	GO:0061982
	GO:BP	regulation of spindle checkpoint	0.000461385	GO:0090231
	GO:BP	mitotic centrosome separation	0.000461385	GO:0007100
	GO:BP	regulation of mitotic cell cycle spindle assembly checkpoint	0.000461385	GO:0090266
	GO:BP	regulation of mitotic spindle checkpoint	0.000461385	GO:1903504
	GO:BP	chromatin remodeling at centromere	0.000461385	GO:0031055
	GO:BP	attachment of mitotic spindle microtubules to kinetochore	0.000461385	GO:0051315
	GO:BP	cellular process	0.000475769	GO:0009987
	GO:CC	cleavage furrow	0.000485474	GO:0032154
	GO:CC	cell division site	0.000515299	GO:0032153
	GO:BP	positive regulation of nuclear division	0.000600097	GO:0051785
	GO:BP	antigen processing and presentation of peptide antigen	0.000605399	GO:0048002
	GO:BP	centrosome separation	0.000608841	GO:0051299
	GO:BP	signal transduction by p53 class mediator	0.000654907	GO:0072331
	GO:BP	DNA integrity checkpoint	0.0006564	GO:0031570
	GO:BP	positive regulation of cell cycle G2/M phase transition	0.0006564	GO:1902751
	GO:BP	regulation of cyclin-dependent protein serine/threonine kinase activity	0.000665698	GO:0000079
	GO:BP	cellular component assembly	0.000692955	GO:0022607
	GO:BP	regulation of meiotic cell cycle	0.000708711	GO:0051445
	GO:BP	regulation of microtubule cytoskeleton organization	0.000733308	GO:0070507

	GO:CC	serine/threonine protein kinase complex	0.000743539	GO:1902554
	GO:BP	nucleoside salvage	0.000767009	GO:0043174
	GO:BP	anatomical structure homeostasis	0.000767009	GO:0060249
	GO:CC	replication fork protection complex	0.000841081	GO:0031298
	GO:BP	regulation of cyclin-dependent protein kinase activity	0.000857601	GO:1904029
	GO:BP	female meiotic nuclear division	0.000868577	GO:0007143
	GO:MF	drug binding	0.000876325	GO:0008144
	GO:BP	positive regulation of chromosome segregation	0.001008036	GO:0051984
	GO:MF	protein binding	0.001021848	GO:0005515
	GO:BP	RNA phosphodiester bond hydrolysis, endonucleolytic	0.001107362	GO:0090502
	GO:CC	DNA polymerase complex	0.001131414	GO:0042575
	GO:CC	cyclin B1-CDK1 complex	0.001231182	GO:0097125
	GO:CC	condensed nuclear chromosome inner kinetochore	0.001231182	GO:0000941
	GO:CC	polymeric cytoskeletal fiber	0.001231182	GO:0099513
	GO:BP	sister chromatid cohesion	0.001442497	GO:0007062
	GO:BP	reproductive process	0.001475539	GO:0022414
	GO:BP	reproduction	0.001529254	GO:0000003
	GO:CC	contractile ring	0.001629926	GO:0070938
	GO:BP	cellular component biogenesis	0.001814303	GO:0044085
	GO:MF	kinase binding	0.001818643	GO:0019900
	GO:BP	retrograde vesicle-mediated transport, Golgi to endoplasmic reticulum	0.001858909	GO:0006890
	GO:CC	protein kinase complex	0.001934846	GO:1902911
	GO:BP	regulation of meiotic cell cycle phase transition	0.001957039	GO:1901993
	GO:BP	meiotic cell cycle phase transition	0.001957039	GO:0044771
	GO:MF	nucleotide binding	0.001968657	GO:0000166
	GO:MF	motor activity	0.001968657	GO:0003774
	GO:MF	nucleoside phosphate binding	0.001968657	GO:1901265
	GO:MF	protein kinase binding	0.001968657	GO:0019901
	GO:CC	nuclear replisome	0.002021096	GO:0043601
	GO:BP	histone exchange	0.002042865	GO:0043486
	GO:BP	chromatin organization involved in regulation of transcription	0.002137858	GO:0034401
	GO:BP	positive regulation of cytokinesis	0.002163763	GO:0032467
	GO:BP	nucleoside biosynthetic process	0.002163763	GO:0009163
	GO:MF	catalytic activity, acting on DNA	0.002297337	GO:0140097
	GO:BP	cellular response to radiation	0.002370855	GO:0071478
	GO:BP	nucleic acid metabolic process	0.002465273	GO:0090304
	GO:BP	positive regulation of transferase activity	0.002661565	GO:0051347
	GO:CC	replisome	0.002675151	GO:0030894
	GO:BP	regulation of microtubule-based process	0.002731181	GO:0032886
	GO:BP	antigen processing and presentation	0.002731181	GO:0019882
	GO:BP	regulation of mitotic centrosome separation	0.002766932	GO:0046602
	GO:MF	transferase activity, transferring phosphorus-containing groups	0.002846128	GO:0016772

	GO:MF	RNA-DNA hybrid ribonuclease activity	0.002848092	GO:0004523
	GO:MF	purine ribonucleotide binding	0.002848092	GO:0032555
	GO:MF	purine ribonucleoside triphosphate binding	0.002848092	GO:0035639
	GO:BP	regulation of protein kinase activity	0.002877636	GO:0045859
	GO:BP	cellular metabolic process	0.002926853	GO:0044237
	GO:MF	small molecule binding	0.00298398	GO:0036094
	GO:MF	purine nucleotide binding	0.002988102	GO:0017076
	GO:MF	ribonucleotide binding	0.002988102	GO:0032553
	GO:BP	protein-containing complex subunit organization	0.003023207	GO:0043933
	GO:BP	glycosyl compound biosynthetic process	0.003309215	GO:1901659
	GO:MF	endoribonuclease activity	0.003400996	GO:0004521
	GO:CC	transferase complex, transferring phosphorus-containing groups	0.003406896	GO:0061695
	GO:CC	nucleosome	0.003406896	GO:0000786
	GO:BP	regulation of ubiquitin protein ligase activity	0.003619788	GO:1904666
	GO:CC	Flemming body	0.003822718	GO:0090543
	GO:MF	kinase activity	0.004132404	GO:0016301
	GO:MF	endonuclease activity	0.004140533	GO:0004519
	GO:BP	aging	0.004298133	GO:0007568
	GO:BP	protein-containing complex assembly	0.004541405	GO:0065003
	GO:BP	microtubule-based movement	0.004710615	GO:0007018
	GO:BP	phosphate-containing compound metabolic process	0.004931199	GO:0006796
	GO:BP	pyrimidine nucleoside salvage	0.005041483	GO:0043097
	GO:BP	pyrimidine-containing compound salvage	0.005041483	GO:0008655
	GO:BP	histone-serine phosphorylation	0.005041483	GO:0035404
	GO:CC	chromocenter	0.005319889	GO:0010369
	GO:BP	oocyte maturation	0.00559444	GO:0001556
	GO:MF	cyclin-dependent protein serine/threonine kinase regulator activity	0.005723766	GO:0016538
	GO:MF	ATPase activity, coupled	0.005742043	GO:0042623
	GO:BP	phosphorus metabolic process	0.005981778	GO:0006793
	GO:BP	nucleobase-containing small molecule biosynthetic process	0.006008441	GO:0034404
	GO:CC	Ndc80 complex	0.006222909	GO:0031262
	GO:CC	NMS complex	0.006222909	GO:0031617
	GO:CC	spindle pole centrosome	0.006222909	GO:0031616
	GO:BP	chromatin organization involved in negative regulation of transcription	0.006359712	GO:0097549
	GO:BP	chromatin silencing	0.006359712	GO:0006342
	GO:BP	protein localization to organelle	0.006359712	GO:0033365
	GO:BP	mitotic nuclear envelope disassembly	0.006452718	GO:0007077
	GO:BP	regulation of kinase activity	0.006623252	GO:0043549
	GO:BP	negative regulation of cellular component organization	0.006783536	GO:0051129
	GO:BP	female gamete generation	0.006859053	GO:0007292
	GO:CC	euchromatin	0.007071232	GO:0000791
	GO:BP	regulation of signal transduction by p53 class mediator	0.007155046	GO:1901796

	GO:CC	pronucleus	0.007289827	GO:0045120
	GO:BP	positive regulation of G2/MI transition of meiotic cell cycle	0.007384969	GO:0110032
	GO:BP	regulation of G2/MI transition of meiotic cell cycle	0.007384969	GO:0110030
	GO:BP	spindle localization	0.007384969	GO:0051653
	GO:BP	G2/MI transition of meiotic cell cycle	0.007384969	GO:0008315
	GO:BP	hexitol metabolic process	0.007384969	GO:0006059
	GO:BP	negative regulation of DNA duplex unwinding	0.007384969	GO:1905463
	GO:BP	DNA replication preinitiation complex assembly	0.007384969	GO:0071163
	GO:BP	nucleic acid phosphodiester bond hydrolysis	0.0075508	GO:0090305
	GO:BP	nitrogen compound metabolic process	0.00852246	GO:0006807
	GO:MF	enzyme binding	0.008663574	GO:0019899
	GO:BP	cell population proliferation	0.009273413	GO:0008283
	GO:BP	meiosis I	0.00954006	GO:0007127
	GO:MF	chromatin binding	0.009597038	GO:0003682
	GO:MF	ribonuclease activity	0.009597038	GO:0004540
	GO:MF	hydrolase activity	0.009689012	GO:0016787
	GO:MF	DNA-dependent ATPase activity	0.009689012	GO:0008094
	GO:MF	carbohydrate derivative binding	0.009689012	GO:0097367
	GO:BP	nucleotide salvage	0.009784375	GO:0043173
	GO:CC	DNA replication preinitiation complex	0.009806258	GO:0031261
	GO:BP	regulation of cell cycle checkpoint	0.009880454	GO:1901976
	GO:BP	DNA geometric change	0.009918106	GO:0032392
	GO:CC	germ cell nucleus	0.009940129	GO:0043073
	GO:BP	DNA biosynthetic process	0.010673536	GO:0071897
	GO:BP	RNA phosphodiester bond hydrolysis	0.010802376	GO:0090501
	GO:BP	regulation of catalytic activity	0.010802376	GO:0050790
	GO:BP	negative regulation of gene expression, epigenetic	0.010802376	GO:0045814
	GO:BP	cell cycle arrest	0.011260796	GO:0007050
	GO:MF	anion binding	0.011371412	GO:0043168
	GO:BP	protein localization to cytoskeleton	0.011428025	GO:0044380
	GO:BP	regulation of protein serine/threonine kinase activity	0.011518008	GO:0071900
	GO:BP	mitotic DNA replication	0.011634703	GO:1902969
	GO:BP	cellular protein-containing complex assembly	0.01256612	GO:0034622
	GO:BP	cellular metabolic compound salvage	0.01336645	GO:0043094
	GO:BP	cellular response to environmental stimulus	0.013503175	GO:0104004
	GO:BP	cellular response to abiotic stimulus	0.013503175	GO:0071214
	GO:BP	DNA replication, removal of RNA primer	0.013503175	GO:0043137
	GO:BP	nuclear envelope disassembly	0.013696685	GO:0051081
	GO:BP	membrane disassembly	0.013696685	GO:0030397
	GO:BP	ATP-dependent chromatin remodeling	0.013696685	GO:0043044
	GO:BP	pyrimidine nucleoside biosynthetic process	0.013696685	GO:0046134
	GO:MF	ribonuclease A activity	0.014124879	GO:0004522

	GO:MF	double-stranded DNA exodeoxyribonuclease activity	0.014124879	GO:0008309
	GO:MF	DNA primase activity	0.014124879	GO:0003896
	GO:BP	positive regulation of protein kinase activity	0.015775292	GO:0045860
	GO:BP	multi-organism reproductive process	0.015775292	GO:0044703
	GO:BP	cellular response to estradiol stimulus	0.015846668	GO:0071392
	GO:BP	establishment of mitotic spindle localization	0.015846668	GO:0040001
	GO:BP	DNA replication checkpoint	0.015927477	GO:0000076
	GO:BP	mitotic chromosome condensation	0.015927477	GO:0007076
	GO:BP	kinetochore assembly	0.015927477	GO:0051382
	GO:BP	midbody abscission	0.015927477	GO:0061952
	GO:BP	positive regulation of telomere capping	0.015927477	GO:1904355
	GO:BP	cellular macromolecule metabolic process	0.016617616	GO:0044260
	GO:BP	primary metabolic process	0.016955183	GO:0044238
	GO:BP	glycosyl compound metabolic process	0.017242975	GO:1901657
	GO:BP	small molecule metabolic process	0.017580119	GO:0044281
	GO:BP	gamete generation	0.017763328	GO:0007276
	GO:BP	regulation of cellular response to stress	0.018128955	GO:0080135
	GO:BP	cellular response to reactive oxygen species	0.018252629	GO:0034614
	GO:BP	regulation of exit from mitosis	0.018324463	GO:0007096
	GO:BP	heterocycle catabolic process	0.018324463	GO:0046700
	GO:BP	cellular nitrogen compound catabolic process	0.018548964	GO:0044270
	GO:MF	heterocyclic compound binding	0.018680431	GO:1901363
	GO:MF	phosphotransferase activity, alcohol group as acceptor	0.018680431	GO:0016773
	GO:MF	damaged DNA binding	0.018680431	GO:0003684
	GO:MF	single-stranded DNA helicase activity	0.018680431	GO:0017116
	GO:BP	response to radiation	0.018844435	GO:0009314
	GO:MF	organic cyclic compound binding	0.019158134	GO:0097159
	GO:BP	cofactor catabolic process	0.019505642	GO:0051187
	GO:BP	positive regulation of mitochondrial ATP synthesis coupled electron transport	0.019760656	GO:1905448
	GO:BP	positive regulation of exit from mitosis	0.019760656	GO:0031536
	GO:BP	positive regulation of meiotic cell cycle phase transition	0.019760656	GO:1901995
	GO:BP	UMP salvage	0.019760656	GO:0044206
	GO:BP	pyrimidine ribonucleotide salvage	0.019760656	GO:0010138
	GO:BP	vitamin B6 metabolic process	0.019760656	GO:0042816
	GO:BP	regulation of DNA duplex unwinding	0.019760656	GO:1905462
	GO:BP	pyrimidine nucleotide salvage	0.019760656	GO:0032262
	GO:BP	regulation of cellular component organization	0.019855105	GO:0051128
	GO:MF	3'-5' DNA helicase activity	0.020847031	GO:0043138
	GO:BP	phosphorylation	0.020945041	GO:0016310
	GO:MF	protein dimerization activity	0.022110187	GO:0046983
	GO:BP	cellular response to ionizing radiation	0.022829689	GO:0071479

	GO:BP	aromatic compound catabolic process	0.022934081	GO:0019439
	GO:BP	regulation of DNA metabolic process	0.023307815	GO:0051052
	GO:BP	response to UV	0.023307815	GO:0009411
	GO:BP	error-prone translesion synthesis	0.023562672	GO:0042276
	GO:BP	positive regulation of cellular process	0.023562672	GO:0048522
	GO:BP	organic substance metabolic process	0.023938175	GO:0071704
	GO:BP	chromatin remodeling	0.024132208	GO:0006338
	GO:BP	regulation of centrosome duplication	0.024503796	GO:0010824
	GO:BP	cortical actin cytoskeleton organization	0.024503796	GO:0030866
	GO:BP	nucleoside monophosphate metabolic process	0.025013432	GO:0009123
	GO:MF	protein serine/threonine/tyrosine kinase activity	0.025781292	GO:0004712
	GO:MF	kinesin binding	0.025781292	GO:0019894
	GO:CC	condensin complex	0.025791772	GO:0000796
	GO:CC	axon hillock	0.025791772	GO:0043203
	GO:MF	carbohydrate kinase activity	0.025814441	GO:0019200
	GO:BP	signal transduction involved in DNA damage checkpoint	0.026173203	GO:0072422
	GO:BP	nucleoside monophosphate biosynthetic process	0.026173203	GO:0009124
	GO:BP	multicellular organismal reproductive process	0.026173203	GO:0048609
	GO:BP	signal transduction involved in DNA integrity checkpoint	0.026173203	GO:0072401
	GO:BP	positive regulation of chromosome organization	0.026173203	GO:2001252
	GO:CC	mitotic spindle pole	0.026905607	GO:0097431
	GO:BP	nucleoside monophosphate catabolic process	0.026956101	GO:0009125
	GO:BP	regulation of DNA topoisomerase (ATP-hydrolyzing) activity	0.026956101	GO:2000371
	GO:BP	DNA replication, synthesis of RNA primer	0.026956101	GO:0006269
	GO:BP	nucleoside metabolic process	0.026956101	GO:0009116
	GO:BP	positive regulation of attachment of spindle microtubules to kinetochore	0.026956101	GO:0051987
	GO:BP	mitotic DNA integrity checkpoint	0.026956101	GO:0044774
	GO:BP	positive regulation of xenophagy	0.026956101	GO:1904417
	GO:BP	signal transduction involved in cell cycle checkpoint	0.026956101	GO:0072395
	GO:BP	regulation of xenophagy	0.026956101	GO:1904415
	GO:BP	galactose catabolic process via UDP-galactose	0.026956101	GO:0033499
	GO:BP	positive regulation of DNA topoisomerase (ATP-hydrolyzing) activity	0.026956101	GO:2000373
	GO:MF	histone serine kinase activity	0.027106614	GO:0035174
	GO:CC	chromatin	0.027544552	GO:0000785
	GO:MF	nucleoside-triphosphatase activity	0.02780557	GO:0017111
	GO:BP	DNA duplex unwinding	0.028149661	GO:0032508
	GO:CC	supramolecular fiber	0.028153353	GO:0099512
	GO:BP	regulation of sister chromatid cohesion	0.028599255	GO:0007063
	GO:BP	positive regulation of mitotic sister chromatid segregation	0.028599255	GO:0062033
	GO:BP	regulation of binding	0.028599255	GO:0051098
	GO:BP	pyrimidine ribonucleotide biosynthetic process	0.028599255	GO:0009220
	GO:BP	negative regulation of cellular senescence	0.028599255	GO:2000773

	GO:BP	organic substance biosynthetic process	0.028888628	GO:1901576
	GO:BP	multicellular organism reproduction	0.029428765	GO:0032504
	GO:BP	DNA damage checkpoint	0.029909246	GO:0000077
	GO:CC	supramolecular polymer	0.030536438	GO:0099081
	GO:BP	animal organ regeneration	0.030940287	GO:0031100
	GO:BP	oocyte development	0.031077937	GO:0048599
	GO:BP	establishment of spindle localization	0.031077937	GO:0051293
	GO:BP	regulation of DNA replication	0.031087199	GO:0006275
	GO:MF	endonuclease activity, active with either ribo- or deoxyribonucleic acids and producing 3'-phosphomonoesters	0.031188392	GO:0016894
	GO:MF	single-stranded DNA binding	0.031188392	GO:0003697
	GO:MF	DNA replication origin binding	0.031188392	GO:0003688
	GO:MF	DNA helicase activity	0.031344417	GO:0003678
	GO:CC	nuclear euchromatin	0.031450749	GO:0005719
	GO:BP	mitotic sister chromatid cohesion	0.031708585	GO:0007064
	GO:BP	mitotic cytokinetic process	0.031708585	GO:1902410
	GO:BP	response to reactive oxygen species	0.032135541	GO:0000302
	GO:BP	biosynthetic process	0.032135541	GO:0009058
	GO:MF	ATP-dependent microtubule motor activity	0.032908921	GO:1990939
	GO:BP	pre-replicative complex assembly	0.033786761	GO:0036388
	GO:BP	meiotic cell cycle checkpoint	0.033786761	GO:0033313
	GO:BP	organic cyclic compound catabolic process	0.033786761	GO:1901361
	GO:BP	response to organic cyclic compound	0.033786761	GO:0014070
	GO:BP	porphyrin-containing compound catabolic process	0.033786761	GO:0006787
	GO:BP	L-serine biosynthetic process	0.033786761	GO:0006564
	GO:BP	tetrapyrrole catabolic process	0.033786761	GO:0033015
	GO:BP	pigment catabolic process	0.033786761	GO:0046149
	GO:BP	pre-replicative complex assembly involved in cell cycle DNA replication	0.033786761	GO:1902299
	GO:BP	pre-replicative complex assembly involved in nuclear cell cycle DNA replication	0.033786761	GO:0006267
	GO:BP	spindle assembly involved in meiosis	0.033786761	GO:0090306
	GO:BP	positive regulation of spindle checkpoint	0.033786761	GO:0090232
	GO:BP	positive regulation of mitotic cell cycle spindle assembly checkpoint	0.033786761	GO:0090267
	GO:BP	heme catabolic process	0.033786761	GO:0042167
	GO:BP	cellular response to UV	0.03414214	GO:0034644
	GO:BP	pyrimidine ribonucleotide metabolic process	0.03414214	GO:0009218
	GO:MF	histone binding	0.034273527	GO:0042393
	GO:MF	catalytic activity, acting on RNA	0.034679327	GO:0140098
	GO:BP	activation of protein kinase activity	0.035198425	GO:0032147
	GO:BP	protein phosphorylation	0.035398862	GO:0006468
	GO:MF	protein kinase activity	0.03551753	GO:0004672
	GO:BP	regulation of molecular function	0.036168468	GO:0065009

	GO:BP	negative regulation of response to DNA damage stimulus	0.037456344	GO:2001021
	GO:BP	cell aging	0.037456344	GO:0007569
	GO:BP	response to stress	0.037589227	GO:0006950
	GO:BP	long-chain fatty-acyl-CoA metabolic process	0.037608228	GO:0035336
	GO:MF	oxidoreductase activity, acting on the CH-CH group of donors, NAD or NADP as acceptor	0.039423452	GO:0016628
	GO:BP	regulation of cell cycle G1/S phase transition	0.040682332	GO:1902806
	GO:MF	5'-flap endonuclease activity	0.040817999	GO:0017108
	GO:MF	identical protein binding	0.04089581	GO:0042802
	GO:BP	protein localization to microtubule cytoskeleton	0.041093257	GO:0072698
	GO:BP	oocyte differentiation	0.041093257	GO:0009994
	GO:BP	carbohydrate phosphorylation	0.041548472	GO:0046835
	GO:BP	regulation of telomere capping	0.041548472	GO:1904353
	GO:MF	nuclease activity	0.041663522	GO:0004518
	GO:BP	positive regulation of histone phosphorylation	0.042026621	GO:0033129
	GO:BP	positive regulation of kinase activity	0.042026621	GO:0033674
	GO:BP	meiotic sister chromatid cohesion	0.042026621	GO:0051177
	GO:BP	meiotic sister chromatid segregation	0.042026621	GO:0045144
	GO:BP	regulation of mitochondrial ATP synthesis coupled electron transport	0.042026621	GO:1905446
	GO:BP	positive regulation of centrosome duplication	0.042026621	GO:0010825
	GO:BP	female meiosis I	0.042026621	GO:0007144
	GO:BP	DNA damage induced protein phosphorylation	0.042026621	GO:0006975
	GO:BP	reciprocal meiotic recombination	0.042604586	GO:0007131
	GO:BP	interstrand cross-link repair	0.042604586	GO:0036297
	GO:BP	positive regulation of cell division	0.043659503	GO:0051781
	GO:BP	cofactor metabolic process	0.043659503	GO:0051186
	GO:MF	pyrophosphatase activity	0.043722298	GO:0016462
	GO:MF	cytoskeletal protein binding	0.043722298	GO:0008092
	GO:BP	regulation of reproductive process	0.044192401	GO:2000241
	GO:MF	hydrolase activity, acting on acid anhydrides, in phosphorus-containing anhydrides	0.044607088	GO:0016818
	GO:MF	hydrolase activity, acting on acid anhydrides	0.044607088	GO:0016817
	GO:BP	homologous recombination	0.044970722	GO:0035825
	GO:BP	regulation of ubiquitin-protein transferase activity	0.044970722	GO:0051438
	GO:MF	hydrolase activity, acting on ester bonds	0.045680829	GO:0016788
	GO:MF	cyclin-dependent protein serine/threonine kinase activator activity	0.045680829	GO:0061575
	GO:MF	flap endonuclease activity	0.045680829	GO:0048256
	GO:BP	male gamete generation	0.046016894	GO:0048232
	GO:BP	zymogen activation	0.047657366	GO:0031638
	GO:BP	pyrimidine-containing compound biosynthetic process	0.047657366	GO:0072528
	GO:CC	centriole	0.048324054	GO:0005814
	GO:BP	negative regulation of cell aging	0.048720539	GO:0090344

	GO:BP	catabolic process	0.048720539	GO:0009056
	GO:BP	oogenesis	0.049282914	GO:0048477
	GO:BP	sexual reproduction	0.049358026	GO:0019953
	GO:BP	cellular response to oxidative stress	0.049425648	GO:0034599
	KEGG	Cell cycle	1.17314E-16	04110
	KEGG	DNA replication	1.27094E-10	03030
	KEGG	Oocyte meiosis	8.6754E-09	04114
	KEGG	Metabolic pathways	7.32177E-08	01100
	KEGG	Progesterone-mediated oocyte maturation	1.72373E-07	04914
	KEGG	Human T-cell leukemia virus 1 infection	8.87876E-07	05166
	KEGG	Cellular senescence	6.08505E-06	04218
	KEGG	Human immunodeficiency virus 1 infection	5.04423E-05	05170
	KEGG	p53 signaling pathway	0.001075312	04115
	KEGG	Apoptosis	0.001849018	04210
	KEGG	Necroptosis	0.004217046	04217
	KEGG	Alcoholism	0.007007583	05034
	KEGG	Pathways in cancer	0.007007583	05200
	KEGG	Viral carcinogenesis	0.010067511	05203
	KEGG	Drug metabolism - other enzymes	0.012126955	00983
	KEGG	MicroRNAs in cancer	0.015619636	05206
	KEGG	Hepatitis B	0.020513148	05161
	KEGG	Fanconi anemia pathway	0.036550593	03460
	KEGG	Pyrimidine metabolism	0.040056525	00240
**Upregulated in the K5/6-only_Lund group**	**Source**	**Terms**	**FDR**	**Term ID**
	GO:BP	cellular developmental process	0.000177784	GO:0048869
	GO:BP	cell differentiation	0.000177784	GO:0030154
	GO:BP	animal organ morphogenesis	0.000188769	GO:0009887
	GO:BP	developmental process	0.001054526	GO:0032502
	GO:BP	anatomical structure development	0.001222627	GO:0048856
	GO:BP	regulation of developmental process	0.001222627	GO:0050793
	GO:BP	animal organ development	0.001523567	GO:0048513
	GO:BP	anatomical structure morphogenesis	0.001525066	GO:0009653
	GO:BP	system development	0.001592106	GO:0048731
	GO:CC	intracellular	0.002846084	GO:0005622
	GO:BP	epithelium development	0.004653434	GO:0060429
	GO:BP	multicellular organism development	0.006055579	GO:0007275
	GO:BP	tissue development	0.009369695	GO:0009888
	GO:BP	regulation of cellular metabolic process	0.009369695	GO:0031323
	GO:BP	positive regulation of cellular process	0.009369695	GO:0048522
	GO:BP	regulation of multicellular organismal development	0.012068113	GO:2000026
	GO:BP	positive regulation of biological process	0.012068113	GO:0048518
	GO:BP	positive regulation of metabolic process	0.012986386	GO:0009893

	GO:BP	morphogenesis of an epithelium	0.012986386	GO:0002009
	GO:BP	regulation of metabolic process	0.012986386	GO:0019222
	GO:BP	regulation of multicellular organismal process	0.012986386	GO:0051239
	GO:BP	regulation of cell differentiation	0.012986386	GO:0045595
	GO:MF	sequence-specific DNA binding	0.018892921	GO:0043565
	GO:BP	regulation of somitogenesis	0.023440867	GO:0014807
	GO:BP	metabolic process	0.023440867	GO:0008152
	GO:BP	negative regulation of Notch signaling pathway involved in somitogenesis	0.02836262	GO:1902367
	GO:BP	regulation of Notch signaling pathway involved in somitogenesis	0.02836262	GO:1902366
	GO:BP	Notch signaling pathway involved in somitogenesis	0.02836262	GO:1902359
	GO:BP	epidermis development	0.028715024	GO:0008544
	GO:BP	osteoblast differentiation	0.028715024	GO:0001649
	GO:BP	regulation of macromolecule metabolic process	0.028715024	GO:0060255
	GO:BP	positive regulation of cellular metabolic process	0.028715024	GO:0031325
	GO:BP	regulation of cell population proliferation	0.028715024	GO:0042127
	GO:BP	neuronal stem cell population maintenance	0.028715024	GO:0097150
	GO:BP	negative regulation of cell differentiation	0.029055458	GO:0045596
	GO:BP	epithelial cell differentiation	0.038959071	GO:0030855
	GO:BP	embryonic skeletal system development	0.038959071	GO:0048706
	GO:BP	prostate glandular acinus development	0.038978512	GO:0060525
	GO:BP	regulation of nitrogen compound metabolic process	0.038978512	GO:0051171
	GO:BP	ossification	0.038978512	GO:0001503
	GO:BP	organic substance metabolic process	0.040120487	GO:0071704
	GO:BP	epithelial cell differentiation involved in prostate gland development	0.040609741	GO:0060742
	GO:BP	prostate gland morphogenesis	0.040609741	GO:0060512
	GO:BP	regulation of RNA metabolic process	0.040609741	GO:0051252
	GO:BP	negative regulation of developmental process	0.040609741	GO:0051093
	GO:BP	sensory organ development	0.040609741	GO:0007423
	GO:BP	hemidesmosome assembly	0.040609741	GO:0031581
	GO:BP	primary metabolic process	0.040609741	GO:0044238
	GO:BP	striatal medium spiny neuron differentiation	0.040609741	GO:0021773
	GO:BP	prostate gland development	0.040609741	GO:0030850
	GO:BP	digestive tract morphogenesis	0.040609741	GO:0048546
	GO:BP	distal tubule development	0.040609741	GO:0072017
	GO:BP	skeletal system morphogenesis	0.040609741	GO:0048705
	GO:BP	regulation of primary metabolic process	0.040609741	GO:0080090
	GO:BP	skin development	0.040609741	GO:0043588
	GO:BP	postsynaptic membrane assembly	0.040609741	GO:0097104
	GO:BP	regulation of cellular biosynthetic process	0.041235653	GO:0031326
	GO:BP	cellular macromolecule metabolic process	0.041235653	GO:0044260
	GO:BP	negative regulation of epidermal cell differentiation	0.042091769	GO:0045605

	GO:BP	cellular metabolic process	0.042091769	GO:0044237
	GO:BP	regulation of biosynthetic process	0.042091769	GO:0009889
	GO:BP	negative regulation of biosynthetic process	0.042091769	GO:0009890
	GO:BP	macromolecule metabolic process	0.042091769	GO:0043170
	GO:MF	sequence-specific double-stranded DNA binding	0.044149101	GO:1990837
	GO:MF	double-stranded DNA binding	0.044149101	GO:0003690
	GO:BP	anterior/posterior pattern specification	0.048255585	GO:0009952
	GO:BP	positive regulation of Notch signaling pathway	0.048255585	GO:0045747
	GO:BP	negative regulation of cellular process	0.048255585	GO:0048523
	GO:BP	positive regulation of macromolecule metabolic process	0.048255585	GO:0010604
	KEGG	Pathways in cancer	7.79639E-05	05200
	KEGG	MicroRNAs in cancer	0.005338094	05206
	KEGG	Human papillomavirus infection	0.005446897	05165
	KEGG	Metabolic pathways	0.006973847	01100
**K20-only_Lund vs double-high_Lund**				
**Upregulated in the K20-only_Lund group**	**Source**	**Terms**	**FDR**	**Term ID**
	GO:BP	cell cycle	0.000165062	GO:0007049
	GO:BP	cell cycle process	0.000165062	GO:0022402
	GO:BP	mitotic cell cycle process	0.000327848	GO:1903047
	GO:CC	replication fork	0.000998714	GO:0005657
	GO:BP	DNA packaging	0.00155071	GO:0006323
	GO:BP	chromatin assembly	0.001755994	GO:0031497
	GO:BP	regulation of cell cycle	0.00213358	GO:0051726
	GO:BP	cell division	0.002280645	GO:0051301
	GO:BP	mitotic cell cycle	0.002280645	GO:0000278
	GO:BP	nucleosome organization	0.002498715	GO:0034728
	GO:BP	DNA metabolic process	0.002539175	GO:0006259
	GO:BP	telomere maintenance via semi-conservative replication	0.002539175	GO:0032201
	GO:BP	cell cycle phase transition	0.002539175	GO:0044770
	GO:BP	recombinational repair	0.002539175	GO:0000725
	GO:BP	double-strand break repair via homologous recombination	0.002539175	GO:0000724
	GO:BP	chromatin assembly or disassembly	0.002539175	GO:0006333
	GO:BP	nucleosome assembly	0.002539175	GO:0006334
	GO:BP	nuclear DNA replication	0.002961322	GO:0033260
	GO:BP	cellular response to DNA damage stimulus	0.003402611	GO:0006974
	GO:BP	cell cycle DNA replication	0.00394811	GO:0044786
	GO:CC	spindle pole	0.004037151	GO:0000922
	GO:CC	chromosomal region	0.004037151	GO:0098687
	GO:BP	DNA recombination	0.004721776	GO:0006310
	GO:BP	protein-DNA complex subunit organization	0.005694787	GO:0071824
	GO:BP	chromosome organization	0.00612741	GO:0051276
	GO:BP	guanosine-containing compound biosynthetic process	0.006776676	GO:1901070

	GO:CC	chromosome, centromeric region	0.007605086	GO:0000775
	GO:CC	organelle	0.007605086	GO:0043226
	GO:BP	DNA conformation change	0.008580142	GO:0071103
	GO:CC	chromosome	0.00898126	GO:0005694
	GO:BP	chromosome segregation	0.00926221	GO:0007059
	GO:BP	protein-DNA complex assembly	0.009416782	GO:0065004
	GO:CC	condensed chromosome	0.009813816	GO:0000793
	GO:BP	phospholipid biosynthetic process	0.010262108	GO:0008654
	GO:BP	double-strand break repair	0.010262108	GO:0006302
	GO:BP	mitotic cell cycle phase transition	0.010606909	GO:0044772
	GO:CC	membrane-bounded organelle	0.011174235	GO:0043227
	GO:CC	condensed chromosome inner kinetochore	0.011174235	GO:0000939
	GO:BP	mitotic nuclear division	0.011778534	GO:0140014
	GO:MF	damaged DNA binding	0.012222799	GO:0003684
	GO:BP	cellular response to stress	0.012834445	GO:0033554
	GO:BP	DNA replication	0.012834445	GO:0006260
	GO:CC	microtubule cytoskeleton	0.013272417	GO:0015630
	GO:BP	organic cyclic compound metabolic process	0.014384421	GO:1901360
	GO:BP	DNA replication, removal of RNA primer	0.014473751	GO:0043137
	GO:BP	DNA replication-independent nucleosome assembly	0.016312321	GO:0006336
	GO:BP	DNA replication-independent nucleosome organization	0.017055708	GO:0034724
	GO:CC	intracellular	0.01790843	GO:0005622
	GO:CC	nuclear chromosome	0.01790843	GO:0000228
	GO:BP	ribonucleoside biosynthetic process	0.018754255	GO:0042455
	GO:BP	purine ribonucleoside biosynthetic process	0.018754255	GO:0046129
	GO:BP	purine nucleoside biosynthetic process	0.018754255	GO:0042451
	GO:BP	DNA repair	0.018979795	GO:0006281
	GO:BP	telomere maintenance	0.019263357	GO:0000723
	GO:BP	positive regulation of exit from mitosis	0.019457602	GO:0031536
	GO:BP	positive regulation of cell cycle	0.020794255	GO:0045787
	GO:CC	spindle	0.022432495	GO:0005819
	GO:BP	organelle organization	0.025468114	GO:0006996
	GO:BP	telomere organization	0.026441686	GO:0032200
	GO:BP	galactose catabolic process via UDP-galactose	0.026552324	GO:0033499
	GO:BP	heterocycle metabolic process	0.027465191	GO:0046483
	GO:BP	organophosphate biosynthetic process	0.027465191	GO:0090407
	GO:BP	nuclear division	0.028543679	GO:0000280
	GO:BP	regulation of mitotic cell cycle	0.030637891	GO:0007346
	GO:BP	cellular aromatic compound metabolic process	0.031395231	GO:0006725
	GO:BP	sister chromatid segregation	0.033329575	GO:0000819
	GO:CC	centrosome	0.033801352	GO:0005813
	GO:MF	endoribonuclease activity	0.036075098	GO:0004521

	GO:MF	oxidoreductase activity, acting on the CH-CH group of donors, NAD or NADP as acceptor	0.036075098	GO:0016628
	GO:MF	endonuclease activity	0.036075098	GO:0004519
	GO:MF	carboxylic ester hydrolase activity	0.036075098	GO:0052689
	GO:MF	1-acyl-2-lysophosphatidylserine acylhydrolase activity	0.036075098	GO:0052740
	GO:MF	phospholipase A1 activity	0.036075098	GO:0008970
	GO:MF	nuclease activity	0.036075098	GO:0004518
	GO:MF	RNA-DNA hybrid ribonuclease activity	0.036075098	GO:0004523
	GO:MF	hydrolase activity, acting on ester bonds	0.036075098	GO:0016788
	GO:MF	phosphatidylserine 1-acylhydrolase activity	0.036075098	GO:0052739
	GO:MF	transferase activity, transferring phosphorus-containing groups	0.036075098	GO:0016772
	GO:BP	nuclear chromosome segregation	0.036162287	GO:0098813
	GO:CC	nucleus	0.036181238	GO:0005634
	GO:CC	nuclear replication fork	0.036181238	GO:0043596
	GO:CC	cytoplasm	0.036181238	GO:0005737
	GO:CC	nucleoplasm	0.036515017	GO:0005654
	GO:CC	cytosol	0.037903234	GO:0005829
	GO:CC	condensed nuclear chromosome	0.037903234	GO:0000794
	GO:MF	myristoyl-CoA hydrolase activity	0.04147854	GO:0102991
	GO:BP	DNA damage induced protein phosphorylation	0.041917591	GO:0006975
	GO:CC	condensed chromosome kinetochore	0.046147392	GO:0000777
	GO:MF	racemase and epimerase activity, acting on carbohydrates and derivatives	0.046909155	GO:0016857
	GO:BP	RNA phosphodiester bond hydrolysis, endonucleolytic	0.047730457	GO:0090502
	GO:MF	catalytic activity	0.048786203	GO:0003824
	GO:CC	microtubule organizing center	0.049057127	GO:0005815
	GO:CC	intracellular organelle	0.049057127	GO:0043229
	GO:MF	palmitoyl-CoA hydrolase activity	0.049081315	GO:0016290
	KEGG	Metabolic pathways	2.11839E-06	01100
	KEGG	DNA replication	0.000172832	03030
	KEGG	Apoptosis	0.01642322	04210
**Upregulated in the double-high_Lund group**	**Source**	**Terms**	**FDR**	**Term ID**
	GO:CC	intracellular	0.00314123	GO:0005622
	GO:CC	intracellular membrane-bounded organelle	0.037606381	GO:0043231
	KEGG	Metabolic pathways	0.002349007	01100
	KEGG	Ubiquitin mediated proteolysis	0.031477094	04120
**K5/6-only_Lund vs double-high_Lund**				
**Upregulated in the K5/6-only_Lund group**	**Source**	**Terms**	**FDR**	**Term ID**
	GO:MF	methylputrescine oxidase activity	0.044256869	GO:0052599
	GO:MF	propane-1,3-diamine oxidase activity	0.044256869	GO:0052600
	GO:MF	C-X3-C chemokine receptor activity	0.044256869	GO:0016495
	GO:MF	diamine oxidase activity	0.044256869	GO:0052597

	GO:MF	histamine oxidase activity	0.044256869	GO:0052598
**Upregulated in the double-high_Lund group**	**Source**	**Terms**	**FDR**	**Term ID**
	GO:BP	mitotic sister chromatid separation	0.001419225	GO:0051306
	GO:BP	metaphase/anaphase transition of cell cycle	0.001419225	GO:0044784
	GO:BP	regulation of metaphase/anaphase transition of cell cycle	0.001419225	GO:1902099
	GO:BP	regulation of mitotic metaphase/anaphase transition	0.001419225	GO:0030071
	GO:BP	regulation of mitotic sister chromatid separation	0.001419225	GO:0010965
	GO:BP	regulation of chromosome separation	0.001419225	GO:1905818
	GO:BP	metaphase/anaphase transition of mitotic cell cycle	0.001419225	GO:0007091
	GO:BP	regulation of mitotic sister chromatid segregation	0.001720476	GO:0033047
	GO:BP	negative regulation of meiotic cell cycle phase transition	0.001720476	GO:1901994
	GO:BP	regulation of sister chromatid segregation	0.002565928	GO:0033045
	GO:BP	chromosome separation	0.00343327	GO:0051304
	GO:BP	phosphagen biosynthetic process	0.00343327	GO:0042396
	GO:BP	phosphocreatine biosynthetic process	0.00343327	GO:0046314
	GO:BP	phosphagen metabolic process	0.00343327	GO:0006599
	GO:BP	phosphocreatine metabolic process	0.00343327	GO:0006603
	GO:BP	regulation of chromosome segregation	0.003976411	GO:0051983
	GO:BP	cell cycle	0.005868492	GO:0007049
	GO:BP	cleavage furrow formation	0.005994811	GO:0036089
	GO:BP	regulation of meiotic cell cycle phase transition	0.00624847	GO:1901993
	GO:BP	creatine metabolic process	0.00624847	GO:0006600
	GO:BP	nuclear division	0.00624847	GO:0000280
	GO:BP	mitotic cell cycle	0.00624847	GO:0000278
	GO:BP	meiotic cell cycle phase transition	0.00624847	GO:0044771
	GO:BP	regulation of meiotic cell cycle	0.006678506	GO:0051445
	GO:MF	creatine kinase activity	0.006778949	GO:0004111
	GO:MF	phosphotransferase activity, nitrogenous group as acceptor	0.006778949	GO:0016775
	GO:BP	female gamete generation	0.007873575	GO:0007292
	GO:BP	organelle fission	0.009255393	GO:0048285
	GO:BP	positive regulation of mitotic nuclear division	0.009255393	GO:0045840
	GO:BP	mitotic sister chromatid segregation	0.010075221	GO:0000070
	GO:BP	regulation of cell cycle phase transition	0.011097738	GO:1901987
	GO:BP	regulation of mitotic nuclear division	0.012756034	GO:0007088
	GO:BP	positive regulation of nuclear division	0.014698197	GO:0051785
	GO:BP	sister chromatid segregation	0.017905326	GO:0000819
	GO:BP	regulation of nuclear division	0.017996619	GO:0051783
	GO:BP	meiotic cell cycle process	0.017996619	GO:1903046
	GO:BP	microtubule-based process	0.017996619	GO:0007017
	GO:BP	cellular response to UV	0.017996619	GO:0034644
	GO:BP	negative regulation of meiotic cell cycle	0.018185885	GO:0051447
	GO:BP	protein localization to kinetochore	0.018185885	GO:0034501

	GO:BP	regulation of chromosome organization	0.019242557	GO:0033044
	GO:BP	negative regulation of cell division	0.019414485	GO:0051782
	GO:BP	microtubule cytoskeleton organization	0.019518915	GO:0000226
	GO:BP	positive regulation of transforming growth factor beta production	0.022910233	GO:0071636
	GO:BP	drug metabolic process	0.023091951	GO:0017144
	GO:BP	protein localization to chromosome, centromeric region	0.024143891	GO:0071459
	GO:BP	positive regulation of mitotic sister chromatid segregation	0.025939142	GO:0062033
	GO:BP	cell cycle phase transition	0.030773975	GO:0044770
	GO:BP	meiotic cell cycle	0.035372946	GO:0051321
	GO:BP	regulation of mitotic cell cycle phase transition	0.038450137	GO:1901990
	GO:BP	protein localization to meiotic spindle	0.042034852	GO:1905359
	GO:BP	thyroid gland epithelial cell proliferation	0.042034852	GO:1990789
	GO:BP	regulation of cell cycle	0.042034852	GO:0051726
	GO:BP	nuclear chromosome segregation	0.042034852	GO:0098813
	GO:BP	positive regulation of chromosome segregation	0.042034852	GO:0051984
	GO:BP	cellular response to light stimulus	0.042034852	GO:0071482
	GO:BP	female meiotic nuclear division	0.042034852	GO:0007143
	GO:BP	regulation of mitotic cell cycle	0.042034852	GO:0007346
	GO:BP	negative regulation of microvillus assembly	0.042034852	GO:1903697
	GO:BP	cellular response to non-ionic osmotic stress	0.042034852	GO:0071471
	GO:BP	protein localization to meiotic spindle midzone	0.042034852	GO:1903096
	GO:BP	regulation of thyroid gland epithelial cell proliferation	0.042034852	GO:1904441
	GO:BP	negative regulation of asymmetric cell division	0.042034852	GO:0045769
	GO:BP	meiotic spindle assembly checkpoint	0.042034852	GO:0033316
	GO:BP	negative regulation of thyroid gland epithelial cell proliferation	0.042034852	GO:1904442
	GO:BP	response to non-ionic osmotic stress	0.042034852	GO:0010335
	GO:BP	hepatic stellate cell proliferation	0.042034852	GO:1990922
	GO:BP	positive regulation of hepatic stellate cell proliferation	0.042034852	GO:1904899
	GO:BP	regulation of lateral attachment of mitotic spindle microtubules to kinetochore	0.042034852	GO:1905115
	GO:BP	positive regulation of lateral attachment of mitotic spindle microtubules to kinetochore	0.042034852	GO:1905116
	GO:BP	mitotic nuclear division	0.042034852	GO:0140014
	GO:BP	meiotic spindle checkpoint	0.042034852	GO:0044779
	GO:BP	regulation of hepatic stellate cell proliferation	0.042034852	GO:1904897
	GO:BP	spindle assembly checkpoint	0.042981141	GO:0071173
	GO:BP	mitotic spindle checkpoint	0.042981141	GO:0071174
	GO:BP	regulation of transforming growth factor beta production	0.042981141	GO:0071634
	GO:BP	response to UV	0.042981141	GO:0009411
	GO:BP	mitotic spindle assembly checkpoint	0.042981141	GO:0007094
	GO:BP	spindle checkpoint	0.042981141	GO:0031577
	GO:BP	transforming growth factor beta production	0.044737471	GO:0071604

	GO:BP	cytokinetic process	0.045925643	GO:0032506
	GO:BP	negative regulation of mitotic metaphase/anaphase transition	0.045925643	GO:0045841
	GO:BP	negative regulation of metaphase/anaphase transition of cell cycle	0.047111198	GO:1902100
	GO:BP	cytoskeleton organization	0.047111198	GO:0007010
	GO:CC	spindle pole centrosome	0.048163769	GO:0031616
	GO:BP	negative regulation of mitotic sister chromatid separation	0.048875991	GO:2000816
	GO:BP	regulation of cell cycle process	0.049996763	GO:0010564
	KEGG	Metabolic pathways	0.02988261	01100
**K20-only_Lund vs double-low_Lund**				
**Upregulated in the K20-only_Lund group**	**Source**	**Terms**	**FDR**	**Term ID**
	GO:BP	chromosome organization	1.18632E-12	GO:0051276
	GO:BP	sister chromatid segregation	9.82055E-12	GO:0000819
	GO:BP	mitotic cell cycle process	7.26174E-11	GO:1903047
	GO:BP	mitotic nuclear division	1.46858E-10	GO:0140014
	GO:BP	cell cycle	2.16164E-10	GO:0007049
	GO:BP	chromosome segregation	2.16164E-10	GO:0007059
	GO:BP	cell cycle process	2.16164E-10	GO:0022402
	GO:BP	cell division	5.19895E-10	GO:0051301
	GO:BP	mitotic sister chromatid segregation	6.1783E-10	GO:0000070
	GO:BP	nuclear chromosome segregation	6.1783E-10	GO:0098813
	GO:BP	nuclear division	9.84112E-10	GO:0000280
	GO:BP	mitotic cell cycle	2.51029E-09	GO:0000278
	GO:CC	chromosomal region	4.59666E-09	GO:0098687
	GO:BP	organelle fission	5.30111E-09	GO:0048285
	GO:CC	chromosome, centromeric region	7.22222E-09	GO:0000775
	GO:BP	organelle organization	1.91813E-08	GO:0006996
	GO:CC	spindle	2.15245E-08	GO:0005819
	GO:BP	regulation of chromosome segregation	2.26188E-08	GO:0051983
	GO:BP	microtubule cytoskeleton organization involved in mitosis	2.30223E-08	GO:1902850
	GO:BP	cellular response to DNA damage stimulus	6.74293E-08	GO:0006974
	GO:BP	DNA repair	1.20872E-07	GO:0006281
	GO:BP	DNA metabolic process	2.95524E-07	GO:0006259
	GO:BP	mitotic cell cycle phase transition	3.07524E-07	GO:0044772
	GO:BP	mitotic spindle organization	3.96266E-07	GO:0007052
	GO:BP	regulation of sister chromatid segregation	6.22574E-07	GO:0033045
	GO:CC	chromosome	8.55706E-07	GO:0005694
	GO:BP	cell cycle phase transition	8.93584E-07	GO:0044770
	GO:BP	DNA recombination	9.64973E-07	GO:0006310
	GO:CC	condensed chromosome	1.3109E-06	GO:0000793
	GO:BP	cellular response to stress	1.3182E-06	GO:0033554
	GO:BP	DNA packaging	1.44162E-06	GO:0006323
	GO:BP	regulation of mitotic nuclear division	1.76911E-06	GO:0007088

	GO:BP	spindle organization	2.30501E-06	GO:0007051
	GO:CC	condensed chromosome, centromeric region	3.79403E-06	GO:0000779
	GO:CC	intracellular organelle lumen	3.79403E-06	GO:0070013
	GO:CC	organelle lumen	3.79403E-06	GO:0043233
	GO:CC	membrane-enclosed lumen	3.79403E-06	GO:0031974
	GO:BP	regulation of cell cycle	4.15199E-06	GO:0051726
	GO:CC	spindle pole	4.44671E-06	GO:0000922
	GO:BP	regulation of nuclear division	6.01642E-06	GO:0051783
	GO:BP	negative regulation of mitotic nuclear division	6.01642E-06	GO:0045839
	GO:CC	kinetochore	9.66268E-06	GO:0000776
	GO:CC	condensed chromosome kinetochore	1.2837E-05	GO:0000777
	GO:CC	intracellular membrane-bounded organelle	1.2837E-05	GO:0043231
	GO:CC	intracellular	1.45493E-05	GO:0005622
	GO:CC	nucleus	1.45493E-05	GO:0005634
	GO:BP	regulation of mitotic sister chromatid separation	1.46781E-05	GO:0010965
	GO:BP	mitotic cell cycle checkpoint	1.46781E-05	GO:0007093
	GO:BP	negative regulation of nuclear division	1.59061E-05	GO:0051784
	GO:BP	chromosome separation	1.63249E-05	GO:0051304
	GO:BP	DNA conformation change	1.74839E-05	GO:0071103
	GO:BP	mitotic sister chromatid separation	1.81935E-05	GO:0051306
	GO:BP	negative regulation of mitotic sister chromatid separation	2.07662E-05	GO:2000816
	GO:BP	regulation of chromosome separation	2.13281E-05	GO:1905818
	GO:BP	negative regulation of chromosome separation	2.28927E-05	GO:1905819
	GO:CC	nucleoplasm	2.80065E-05	GO:0005654
	GO:CC	membrane-bounded organelle	2.80065E-05	GO:0043227
	GO:BP	negative regulation of mitotic sister chromatid segregation	2.97337E-05	GO:0033048
	GO:BP	regulation of mitotic sister chromatid segregation	3.59167E-05	GO:0033047
	GO:BP	negative regulation of sister chromatid segregation	3.72179E-05	GO:0033046
	GO:BP	negative regulation of chromosome segregation	4.14717E-05	GO:0051985
	GO:CC	organelle	4.81491E-05	GO:0043226
	GO:CC	chromosome passenger complex	5.13402E-05	GO:0032133
	GO:MF	endonuclease activity	5.14866E-05	GO:0004519
	GO:MF	endoribonuclease activity	5.14866E-05	GO:0004521
	GO:BP	RNA phosphodiester bond hydrolysis, endonucleolytic	5.77286E-05	GO:0090502
	GO:BP	regulation of mitotic cell cycle	6.13954E-05	GO:0007346
	GO:BP	regulation of cell cycle process	6.46357E-05	GO:0010564
	GO:CC	microtubule cytoskeleton	6.83871E-05	GO:0015630
	GO:BP	microtubule cytoskeleton organization	7.31782E-05	GO:0000226
	GO:BP	anaphase-promoting complex-dependent catabolic process	7.43056E-05	GO:0031145
	GO:BP	DNA replication-independent nucleosome assembly	7.55581E-05	GO:0006336
	GO:BP	DNA replication-independent nucleosome organization	8.28906E-05	GO:0034724
	GO:BP	organic cyclic compound metabolic process	8.49715E-05	GO:1901360

	GO:BP	heterocycle metabolic process	8.53912E-05	GO:0046483
	GO:BP	cell cycle checkpoint	0.000102399	GO:0000075
	GO:BP	positive regulation of cell cycle	0.000104214	GO:0045787
	GO:BP	female meiotic nuclear division	0.000104661	GO:0007143
	GO:BP	cellular aromatic compound metabolic process	0.000107015	GO:0006725
	GO:BP	regulation of mitotic metaphase/anaphase transition	0.000109907	GO:0030071
	GO:BP	mitotic spindle assembly	0.000109907	GO:0090307
	GO:CC	cytosol	0.000111434	GO:0005829
	GO:CC	cyclin-dependent protein kinase holoenzyme complex	0.000121549	GO:0000307
	GO:CC	nuclear lumen	0.000121549	GO:0031981
	GO:MF	ribonuclease activity	0.000127965	GO:0004540
	GO:BP	regulation of metaphase/anaphase transition of cell cycle	0.000130085	GO:1902099
	GO:BP	metaphase/anaphase transition of mitotic cell cycle	0.000130085	GO:0007091
	GO:BP	metaphase/anaphase transition of cell cycle	0.000155483	GO:0044784
	GO:BP	regulation of cell cycle phase transition	0.000180223	GO:1901987
	GO:BP	mitotic spindle assembly checkpoint	0.00018444	GO:0007094
	GO:BP	spindle assembly checkpoint	0.00018444	GO:0071173
	GO:BP	spindle checkpoint	0.00018444	GO:0031577
	GO:BP	mitotic spindle checkpoint	0.00018444	GO:0071174
	GO:BP	nucleosome assembly	0.000184814	GO:0006334
	GO:BP	cellular nitrogen compound metabolic process	0.000198428	GO:0034641
	GO:BP	positive regulation of cell cycle process	0.000199264	GO:0090068
	GO:BP	histone phosphorylation	0.000199369	GO:0016572
	GO:MF	catalytic activity	0.000200519	GO:0003824
	GO:BP	negative regulation of mitotic metaphase/anaphase transition	0.00022433	GO:0045841
	GO:BP	regulation of chromosome organization	0.000224404	GO:0033044
	GO:CC	intracellular organelle	0.000227585	GO:0043229
	GO:BP	nucleic acid phosphodiester bond hydrolysis	0.000242102	GO:0090305
	GO:BP	negative regulation of metaphase/anaphase transition of cell cycle	0.000244832	GO:1902100
	GO:BP	meiotic cell cycle	0.000245139	GO:0051321
	GO:BP	chromatin organization	0.000269803	GO:0006325
	GO:BP	spindle assembly	0.000273752	GO:0051225
	GO:BP	cellular metabolic process	0.000286587	GO:0044237
	GO:CC	serine/threonine protein kinase complex	0.000291322	GO:1902554
	GO:BP	G2/M transition of mitotic cell cycle	0.000294161	GO:0000086
	GO:BP	regulation of mitotic cell cycle phase transition	0.000294161	GO:1901990
	GO:CC	nuclear chromosome	0.000304656	GO:0000228
	GO:CC	intracellular non-membrane-bounded organelle	0.000304656	GO:0043232
	GO:CC	midbody	0.000304656	GO:0030496
	GO:CC	non-membrane-bounded organelle	0.000304656	GO:0043228
	GO:CC	centrosome	0.000304656	GO:0005813
	GO:BP	RNA phosphodiester bond hydrolysis	0.000308459	GO:0090501

	GO:BP	CENP-A containing nucleosome assembly	0.000314789	GO:0034080
	GO:BP	CENP-A containing chromatin organization	0.000314789	GO:0061641
	GO:BP	double-strand break repair	0.000316381	GO:0006302
	GO:BP	cellular component organization	0.000316381	GO:0016043
	GO:CC	condensed nuclear chromosome, centromeric region	0.000361311	GO:0000780
	GO:MF	histone kinase activity	0.000371304	GO:0035173
	GO:BP	chromatin assembly	0.000397103	GO:0031497
	GO:BP	cellular component organization or biogenesis	0.000397103	GO:0071840
	GO:CC	microtubule organizing center	0.000398983	GO:0005815
	GO:BP	chromatin remodeling at centromere	0.000411839	GO:0031055
	GO:BP	mitotic metaphase plate congression	0.000411839	GO:0007080
	GO:BP	microtubule-based process	0.000433864	GO:0007017
	GO:BP	cell cycle G2/M phase transition	0.000463769	GO:0044839
	GO:CC	cyclin B1-CDK1 complex	0.000472759	GO:0097125
	GO:BP	negative regulation of mitotic cell cycle	0.000487379	GO:0045930
	GO:CC	condensed nuclear chromosome	0.000504894	GO:0000794
	GO:CC	protein kinase complex	0.000548295	GO:1902911
	GO:CC	cytoplasm	0.00059097	GO:0005737
	GO:BP	negative regulation of cell cycle	0.000597667	GO:0045786
	GO:CC	microtubule associated complex	0.000607375	GO:0005875
	GO:MF	nuclease activity	0.000774002	GO:0004518
	GO:BP	negative regulation of cell cycle process	0.000788386	GO:0010948
	GO:CC	endonuclease complex	0.000815031	GO:1905348
	GO:BP	oocyte maturation	0.000847824	GO:0001556
	GO:BP	organic substance metabolic process	0.000847824	GO:0071704
	GO:BP	nucleosome organization	0.000894749	GO:0034728
	GO:BP	centromere complex assembly	0.000906326	GO:0034508
	GO:BP	chromatin assembly or disassembly	0.001022397	GO:0006333
	GO:MF	RNA-DNA hybrid ribonuclease activity	0.001025294	GO:0004523
	GO:BP	double-strand break repair via homologous recombination	0.001093739	GO:0000724
	GO:BP	recombinational repair	0.001133867	GO:0000725
	GO:BP	meiotic cell cycle process	0.001196353	GO:1903046
	GO:BP	female gamete generation	0.00127335	GO:0007292
	GO:BP	metaphase plate congression	0.001306863	GO:0051310
	GO:BP	histone exchange	0.001402744	GO:0043486
	GO:CC	mitotic spindle	0.001520724	GO:0072686
	GO:BP	negative regulation of chromosome organization	0.001540123	GO:2001251
	GO:CC	condensed nuclear chromosome kinetochore	0.001567432	GO:0000778
	GO:BP	mitotic nuclear envelope disassembly	0.001599906	GO:0007077
	GO:BP	gamete generation	0.001703127	GO:0007276
	GO:BP	primary metabolic process	0.001820878	GO:0044238
	GO:BP	nitrogen compound metabolic process	0.001982098	GO:0006807

	GO:BP	nucleobase-containing compound metabolic process	0.002028999	GO:0006139
	GO:BP	negative regulation of cell cycle phase transition	0.002028999	GO:1901988
	GO:BP	metabolic process	0.002187254	GO:0008152
	GO:CC	peroxisome	0.002324588	GO:0005777
	GO:CC	microbody	0.002324588	GO:0042579
	GO:BP	DNA replication	0.002335666	GO:0006260
	GO:BP	negative regulation of organelle organization	0.002335666	GO:0010639
	GO:BP	mitotic centrosome separation	0.002420067	GO:0007100
	GO:MF	endoribonuclease activity, producing 5'-phosphomonoesters	0.00285947	GO:0016891
	GO:BP	positive regulation of mitotic cell cycle	0.002913546	GO:0045931
	GO:BP	signal transduction involved in DNA integrity checkpoint	0.002913546	GO:0072401
	GO:BP	centrosome separation	0.002913546	GO:0051299
	GO:BP	signal transduction involved in DNA damage checkpoint	0.002913546	GO:0072422
	GO:BP	signal transduction involved in cell cycle checkpoint	0.00308288	GO:0072395
	GO:BP	nuclear envelope disassembly	0.003479342	GO:0051081
	GO:BP	membrane disassembly	0.003479342	GO:0030397
	GO:BP	meiosis I cell cycle process	0.003517254	GO:0061982
	GO:CC	condensed chromosome inner kinetochore	0.003582567	GO:0000939
	GO:BP	sexual reproduction	0.003782959	GO:0019953
	GO:BP	establishment of chromosome localization	0.003800757	GO:0051303
	GO:BP	meiotic nuclear division	0.003944932	GO:0140013
	GO:BP	chromosome localization	0.003973813	GO:0050000
	GO:BP	chromosome condensation	0.00490138	GO:0030261
	GO:BP	oocyte development	0.005720334	GO:0048599
	GO:BP	DNA replication, removal of RNA primer	0.005720334	GO:0043137
	GO:BP	nucleus organization	0.005760527	GO:0006997
	GO:BP	negative regulation of mitotic cell cycle phase transition	0.005832908	GO:1901991
	GO:BP	protein-DNA complex assembly	0.006607885	GO:0065004
	GO:BP	protein localization to chromosome, centromeric region	0.007301626	GO:0071459
	GO:BP	ATP-dependent chromatin remodeling	0.007301626	GO:0043044
	GO:BP	meiotic chromosome segregation	0.00757291	GO:0045132
	GO:BP	positive regulation of mitotic cell cycle phase transition	0.007904222	GO:1901992
	GO:BP	oocyte differentiation	0.007981453	GO:0009994
	GO:CC	endoribonuclease complex	0.008005619	GO:1902555
	GO:BP	interstrand cross-link repair	0.008528469	GO:0036297
	GO:BP	positive regulation of exit from mitosis	0.008739152	GO:0031536
	GO:BP	positive regulation of mitochondrial ATP synthesis coupled electron transport	0.008739152	GO:1905448
	GO:MF	endonuclease activity, active with either ribo- or deoxyribonucleic acids and producing 5'-phosphomonoesters	0.00924625	GO:0016893
	GO:MF	four-way junction DNA binding	0.00924625	GO:0000400
	GO:MF	ribonuclease A activity	0.009384311	GO:0004522

	GO:MF	cyclin-dependent protein serine/threonine kinase regulator activity	0.009384311	GO:0016538
	GO:MF	double-stranded DNA exodeoxyribonuclease activity	0.009384311	GO:0008309
	GO:CC	axon hillock	0.009465101	GO:0043203
	GO:BP	multicellular organismal reproductive process	0.009882639	GO:0048609
	GO:BP	regulation of cyclin-dependent protein serine/threonine kinase activity	0.010106142	GO:0000079
	GO:BP	DNA damage response, signal transduction by p53 class mediator resulting in cell cycle arrest	0.010175176	GO:0006977
	GO:BP	regulation of ubiquitin protein ligase activity	0.010207597	GO:1904666
	GO:CC	replication fork	0.010364576	GO:0005657
	GO:BP	signal transduction involved in mitotic G1 DNA damage checkpoint	0.010597526	GO:0072431
	GO:BP	intracellular signal transduction involved in G1 DNA damage checkpoint	0.010597526	GO:1902400
	GO:BP	sister chromatid cohesion	0.010597526	GO:0007062
	GO:BP	multicellular organism reproduction	0.011026649	GO:0032504
	GO:MF	hydrolase activity	0.011170154	GO:0016787
	GO:BP	regulation of centrosome cycle	0.011176062	GO:0046605
	GO:BP	regulation of cyclin-dependent protein kinase activity	0.011480599	GO:1904029
	GO:BP	signal transduction involved in mitotic DNA damage checkpoint	0.011544944	GO:1902402
	GO:BP	signal transduction involved in mitotic DNA integrity checkpoint	0.011544944	GO:1902403
	GO:BP	regulation of DNA recombination	0.011544944	GO:0000018
	GO:BP	signal transduction involved in mitotic cell cycle checkpoint	0.011544944	GO:0072413
	GO:BP	regulation of cell cycle G2/M phase transition	0.011544944	GO:1902749
	GO:BP	cellular process involved in reproduction in multicellular organism	0.011544944	GO:0022412
	GO:CC	protein-containing complex	0.011588775	GO:0032991
	GO:BP	DNA integrity checkpoint	0.012311113	GO:0031570
	GO:BP	positive regulation of G2/M transition of mitotic cell cycle	0.013276315	GO:0010971
	GO:BP	mitotic DNA integrity checkpoint	0.013368646	GO:0044774
	GO:BP	telomere maintenance	0.013368646	GO:0000723
	GO:CC	microtubule	0.013755776	GO:0005874
	GO:CC	transferase complex, transferring phosphorus-containing groups	0.013755776	GO:0061695
	GO:MF	endonuclease activity, active with either ribo- or deoxyribonucleic acids and producing 3'-phosphomonoesters	0.014090039	GO:0016894
	GO:BP	mitotic G1/S transition checkpoint	0.014093744	GO:0044819
	GO:BP	mitotic G1 DNA damage checkpoint	0.014093744	GO:0031571
	GO:BP	positive regulation of cell cycle phase transition	0.014263833	GO:1901989
	GO:BP	G1 DNA damage checkpoint	0.0147784	GO:0044783
	GO:BP	pigment catabolic process	0.014952787	GO:0046149
	GO:BP	L-serine biosynthetic process	0.014952787	GO:0006564
	GO:BP	heme catabolic process	0.014952787	GO:0042167
	GO:BP	tetrapyrrole catabolic process	0.014952787	GO:0033015
	GO:BP	meiotic cell cycle checkpoint	0.014952787	GO:0033313
	GO:BP	porphyrin-containing compound catabolic process	0.014952787	GO:0006787
	GO:BP	protein-DNA complex subunit organization	0.014952787	GO:0071824

	GO:BP	cellular catabolic process	0.015425916	GO:0044248
	GO:CC	cell	0.015798619	GO:0005623
	GO:BP	spermatogenesis	0.016145495	GO:0007283
	GO:BP	positive regulation of cell cycle G2/M phase transition	0.016522774	GO:1902751
	GO:MF	magnesium ion binding	0.016627611	GO:0000287
	GO:MF	DNA secondary structure binding	0.016627611	GO:0000217
	GO:BP	meiosis I	0.016895619	GO:0007127
	GO:BP	regulation of transferase activity	0.016972435	GO:0051338
	GO:BP	catabolic process	0.017236327	GO:0009056
	GO:CC	protein-DNA complex	0.017275673	GO:0032993
	GO:BP	telomere organization	0.017788178	GO:0032200
	GO:BP	organelle localization	0.017788178	GO:0051640
	GO:BP	establishment of localization in cell	0.018238785	GO:0051649
	GO:BP	fatty acid alpha-oxidation	0.018570431	GO:0001561
	GO:BP	female meiosis I	0.018570431	GO:0007144
	GO:BP	regulation of mitochondrial ATP synthesis coupled electron transport	0.018570431	GO:1905446
	GO:BP	regulation of meiotic nuclear division	0.018723551	GO:0040020
	GO:BP	DNA replication-dependent nucleosome assembly	0.018723551	GO:0006335
	GO:BP	DNA replication-dependent nucleosome organization	0.018723551	GO:0034723
	GO:BP	organic substance catabolic process	0.018877934	GO:1901575
	GO:BP	chromatin remodeling	0.01948724	GO:0006338
	GO:BP	multi-organism reproductive process	0.01948724	GO:0044703
	GO:BP	male gamete generation	0.01948724	GO:0048232
	GO:BP	nucleic acid metabolic process	0.020740775	GO:0090304
	GO:MF	transferase activity, transferring phosphorus-containing groups	0.021048831	GO:0016772
	GO:BP	mismatch repair	0.021465168	GO:0006298
	GO:BP	attachment of spindle microtubules to kinetochore	0.021465168	GO:0008608
	GO:BP	centrosome cycle	0.021465168	GO:0007098
	GO:BP	regulation of mitotic centrosome separation	0.022220411	GO:0046602
	GO:BP	DNA topological change	0.022220411	GO:0006265
	GO:BP	cellular component assembly	0.022389035	GO:0022607
	GO:MF	damaged DNA binding	0.023144616	GO:0003684
	GO:BP	reproductive process	0.024438202	GO:0022414
	GO:BP	animal organ regeneration	0.024438202	GO:0031100
	GO:BP	apoptotic nuclear changes	0.024618077	GO:0030262
	GO:CC	catalytic complex	0.024679278	GO:1902494
	GO:BP	reproduction	0.024786241	GO:0000003
	GO:BP	establishment of organelle localization	0.026196063	GO:0051656
	GO:BP	positive regulation of cellular respiration	0.026587134	GO:1901857
	GO:BP	negative regulation of membrane permeability	0.026587134	GO:1905709
	GO:BP	microtubule organizing center organization	0.02727559	GO:0031023
	GO:BP	signal transduction in response to DNA damage	0.02727559	GO:0042770

	GO:BP	cellular component disassembly involved in execution phase of apoptosis	0.027849327	GO:0006921
	GO:BP	signal transduction by p53 class mediator	0.027873073	GO:0072331
	GO:MF	5'-flap endonuclease activity	0.027983834	GO:0017108
	GO:CC	supramolecular complex	0.029230741	GO:0099080
	GO:CC	chromocenter	0.029230741	GO:0010369
	GO:BP	positive regulation of cell cycle arrest	0.029264413	GO:0071158
	GO:BP	regulation of G2/M transition of mitotic cell cycle	0.029819089	GO:0010389
	GO:CC	cytoskeleton	0.03036371	GO:0005856
	GO:CC	peroxisomal matrix	0.03036371	GO:0005782
	GO:CC	microbody lumen	0.03036371	GO:0031907
	GO:MF	hydrolase activity, acting on ester bonds	0.030593117	GO:0016788
	GO:BP	histone-serine phosphorylation	0.031007442	GO:0035404
	GO:BP	cellular response to iron ion	0.031007442	GO:0071281
	GO:BP	positive regulation of oxidative phosphorylation	0.031007442	GO:1903862
	GO:MF	flap endonuclease activity	0.031153176	GO:0048256
	GO:MF	cyclin-dependent protein serine/threonine kinase activator activity	0.031153176	GO:0061575
	GO:BP	small molecule metabolic process	0.031877877	GO:0044281
	GO:BP	protein localization to chromosome	0.032303176	GO:0034502
	GO:BP	regulation of double-strand break repair	0.032303176	GO:2000779
	GO:CC	pronucleus	0.034341765	GO:0045120
	GO:CC	tertiary granule lumen	0.034341765	GO:1904724
	GO:BP	regulation of organelle organization	0.034537065	GO:0033043
	GO:BP	water-soluble vitamin metabolic process	0.034837842	GO:0006767
	GO:BP	regulation of DNA metabolic process	0.03515706	GO:0051052
	GO:BP	ventricular cardiac muscle cell development	0.035409606	GO:0055015
	GO:BP	negative regulation of meiotic nuclear division	0.035409606	GO:0045835
	GO:BP	L-serine metabolic process	0.035409606	GO:0006563
	GO:BP	positive regulation of ubiquitin protein ligase activity	0.035409606	GO:1904668
	GO:CC	kinesin complex	0.035515841	GO:0005871
	GO:BP	response to stress	0.036150006	GO:0006950
	GO:BP	oogenesis	0.036552324	GO:0048477
	GO:BP	cellular component biogenesis	0.036552324	GO:0044085
	GO:BP	cellular response to hypoxia	0.036857167	GO:0071456
	GO:CC	microbody membrane	0.037425132	GO:0031903
	GO:CC	DNA topoisomerase type II (double strand cut, ATP-hydrolyzing) complex	0.037425132	GO:0009330
	GO:CC	Ire1 complex	0.037425132	GO:1990332
	GO:CC	peroxisomal membrane	0.037425132	GO:0005778
	GO:CC	nucleosome	0.037425132	GO:0000786
	GO:CC	BAX complex	0.037425132	GO:0097144
	GO:CC	germ cell nucleus	0.037425132	GO:0043073
	GO:CC	spindle microtubule	0.037425132	GO:0005876

	GO:CC	Dcp1-Dcp2 complex	0.037425132	GO:0098745
	GO:BP	DNA damage checkpoint	0.039105571	GO:0000077
	GO:BP	oxidation-reduction process	0.039157174	GO:0055114
	GO:BP	somatic hypermutation of immunoglobulin genes	0.040243329	GO:0016446
	GO:BP	alpha-linolenic acid metabolic process	0.040243329	GO:0036109
	GO:MF	protein heterodimerization activity	0.040624138	GO:0046982
	GO:CC	DNA packaging complex	0.042027616	GO:0044815
	GO:CC	mitochondrion	0.042202252	GO:0005739
	GO:MF	endoribonuclease activity, producing 3'-phosphomonoesters	0.043416027	GO:0016892
	GO:BP	cellular component disassembly	0.044974411	GO:0022411
	GO:BP	cellular response to decreased oxygen levels	0.045011332	GO:0036294
	GO:BP	positive regulation of endoplasmic reticulum unfolded protein response	0.045017438	GO:1900103
	GO:BP	somatic diversification of immune receptors via somatic mutation	0.045017438	GO:0002566
	GO:BP	regulation of mitotic cell cycle spindle assembly checkpoint	0.045017438	GO:0090266
	GO:BP	regulation of spindle checkpoint	0.045017438	GO:0090231
	GO:BP	cytoskeleton organization	0.045017438	GO:0007010
	GO:BP	regulation of mitotic spindle checkpoint	0.045017438	GO:1903504
	GO:BP	regulation of attachment of spindle microtubules to kinetochore	0.045017438	GO:0051988
	GO:BP	cell cycle G1/S phase transition	0.045097223	GO:0044843
	GO:BP	organelle assembly	0.046138789	GO:0070925
	GO:BP	regulation of double-strand break repair via homologous recombination	0.04646997	GO:0010569
	GO:MF	Hsp70 protein binding	0.048162635	GO:0030544
	GO:MF	kinase activity	0.048440634	GO:0016301
	GO:MF	catalytic activity, acting on DNA	0.048440634	GO:0140097
	GO:MF	endodeoxyribonuclease activity	0.049347553	GO:0004520
	KEGG	Cell cycle	6.18227E-07	04110
	KEGG	Metabolic pathways	1.95561E-06	01100
	KEGG	Oocyte meiosis	0.000118625	04114
	KEGG	Human T-cell leukemia virus 1 infection	0.000143411	05166
	KEGG	Progesterone-mediated oocyte maturation	0.000367074	04914
	KEGG	p53 signaling pathway	0.001683134	04115
	KEGG	Viral carcinogenesis	0.006700643	05203
	KEGG	Human immunodeficiency virus 1 infection	0.007441179	05170
	KEGG	Cellular senescence	0.020259929	04218
	KEGG	Platinum drug resistance	0.020259929	01524
	KEGG	Peroxisome	0.026269595	04146
**Upregulated in the double-low_Lund group**	**Source**	**Terms**	**FDR**	**Term ID**
	GO:MF	ubiquitin-dependent protein binding	0.00057133	GO:0140036
	GO:MF	ubiquitination-like modification-dependent protein binding	0.00057133	GO:0140035
	KEGG	Metabolic pathways	0.000614264	01100
**K5/6-only_Lund vs double-low_Lund**				
**Upregulated in the K5/6-only_Lund group**	**Source**	**Terms**	**FDR**	**Term ID**

	GO:BP	regulation of somitogenesis	0.013655613	GO:0014807
	GO:BP	Notch signaling pathway involved in somitogenesis	0.013702988	GO:1902359
	GO:BP	negative regulation of Notch signaling pathway involved in somitogenesis	0.013702988	GO:1902367
	GO:BP	regulation of Notch signaling pathway involved in somitogenesis	0.013702988	GO:1902366
	GO:BP	epithelium development	0.014033531	GO:0060429
	GO:BP	cellular response to nutrient levels	0.046975249	GO:0031669
**Upregulated in the double-low_Lund group**	**Source**	**Terms**	**FDR**	**Term ID**
	GO:CC	intracellular	4.40172E-05	GO:0005622
	GO:BP	small molecule metabolic process	0.021708795	GO:0044281
	GO:MF	catalytic activity	0.022959617	GO:0003824
	GO:MF	phosphotransferase activity, nitrogenous group as acceptor	0.022959617	GO:0016775
	GO:MF	deoxycytidine deaminase activity	0.022959617	GO:0047844
	GO:MF	creatine kinase activity	0.022959617	GO:0004111
	GO:BP	phosphagen biosynthetic process	0.024830604	GO:0042396
	GO:BP	oxoacid metabolic process	0.024830604	GO:0043436
	GO:BP	positive regulation of exit from mitosis	0.024830604	GO:0031536
	GO:BP	phosphocreatine metabolic process	0.024830604	GO:0006603
	GO:BP	phosphocreatine biosynthetic process	0.024830604	GO:0046314
	GO:BP	phosphagen metabolic process	0.024830604	GO:0006599
	GO:BP	organic acid metabolic process	0.024830604	GO:0006082
	GO:BP	threonine metabolic process	0.029733526	GO:0006566
	GO:BP	carboxylic acid metabolic process	0.029733526	GO:0019752
	GO:MF	NADP-retinol dehydrogenase activity	0.035783519	GO:0052650
	GO:MF	cytidine deaminase activity	0.035783519	GO:0004126
	GO:MF	calcium-transporting ATPase activity involved in regulation of cardiac muscle cell membrane potential	0.036411784	GO:0086039
	GO:MF	squalene monooxygenase activity	0.036411784	GO:0004506
	GO:MF	propionate CoA-transferase activity	0.036411784	GO:0018729
	GO:MF	lutropin-choriogonadotropic hormone receptor binding	0.036411784	GO:0031775
	GO:MF	glycine C-acetyltransferase activity	0.036411784	GO:0008890
	GO:MF	aldo-keto reductase (NADP) activity	0.036411784	GO:0004033
	GO:MF	C-C chemokine binding	0.036411784	GO:0019957
	GO:MF	phosphoglycerate dehydrogenase activity	0.036411784	GO:0004617
	GO:MF	chemokine receptor activity	0.036411784	GO:0004950
	GO:MF	palmitoyl-CoA 9-desaturase activity	0.036411784	GO:0032896
	GO:MF	chemokine (C-C motif) ligand 21 binding	0.036411784	GO:0035758
	GO:MF	C-acyltransferase activity	0.036411784	GO:0016408
	GO:MF	chemokine (C-C motif) ligand 19 binding	0.036411784	GO:0035757
	GO:MF	G protein-coupled chemoattractant receptor activity	0.036411784	GO:0001637
	GO:MF	C-C motif chemokine 19 receptor activity	0.036411784	GO:0038117
	GO:MF	alkyl hydroperoxide reductase activity	0.036411784	GO:0008785

	GO:MF	alcohol dehydrogenase (NADP+) activity	0.036411784	GO:0008106
	GO:MF	glyceraldehyde oxidoreductase activity	0.036411784	GO:0043795
	GO:MF	C-C chemokine receptor activity	0.036411784	GO:0016493
	GO:MF	C-C motif chemokine 21 receptor activity	0.036411784	GO:0038121
	GO:MF	protein-glucosylgalactosylhydroxylysine glucosidase activity	0.036411784	GO:0047402
	GO:MF	N-acetylgalactosamine-6-phosphate deacetylase activity	0.036411784	GO:0047419
	GO:MF	oxidoreductase activity	0.036411784	GO:0016491
	GO:BP	pyrimidine nucleoside metabolic process	0.041512751	GO:0006213
	GO:BP	creatine metabolic process	0.042513438	GO:0006600
	GO:BP	monocarboxylic acid metabolic process	0.042513438	GO:0032787
	GO:MF	deaminase activity	0.045457353	GO:0019239
	GO:MF	hydrolase activity, acting on carbon-nitrogen (but not peptide) bonds, in cyclic amidines	0.04995469	GO:0016814
	KEGG	Metabolic pathways	2.15142E-07	01100

© 2020 by the authors. Submitted for possible open access publication under the terms and conditions of the Creative Commons Attribution (CC BY) license (http://creativecommons.org/licenses/by/4.0/).
